# Supplementary material for: Safety and pharmacokinetics of VRC07-523LS administered via different routes and doses (HVTN 127/HPTN 087): A Phase I randomized clinical trial
Source: PLoS Med. 2024 Jun 24;21(6):e1004329. doi: 10.1371/journal.pmed.1004329 (PMC11251612; doi:10.1371/journal.pmed.1004329)
Supplement: S1 Table — The env sequences are derived from isolates sequenced from incident acquisition events from the AMP studies. (PDF) [file pmed.1004329.s002.pdf]

	Isolate	VRC07-523LS	VRC01
IC_{50} (µg/mL)	H703_0646_051sN	0.98	7.38
	H703_1471_190s	0.03	0.17
	H703_1750_140Es	0.32	0.99
	H704_0726_080sN	0.91	4.7
	H704_1535_030sN	0.06	0.1
	H704_2544_140eN01	0.16	0.79
	PVO.4	0.17	0.54
IC_{80} (µg/mL)	H703_0646_051sN	2.75	20.45
	H703_1471_190s	0.09	0.41
	H703_1750_140Es	1.14	2.66
	H704_0726_080sN	2.76	15.5
	H704_1535_030sN	0.19	0.27
	H704_2544_140eN01	0.53	2.48
	PVO.4	0.51	1.32

Supplemental Table 1. In vitro IC_{50} and IC_{80} of the clinical lot of two bnAbs (VRC07-523LS and VRC01) against seven Env-pseudotyped viruses. The *env* sequences are derived from isolates sequenced from incident acquisition events from the AMP studies.
