# Supplementary material for: Safety and pharmacokinetics of VRC07-523LS administered via different routes and doses (HVTN 127/HPTN 087): A Phase I randomized clinical trial
Source: PLoS Med. 2024 Jun 24;21(6):e1004329. doi: 10.1371/journal.pmed.1004329 (PMC11251612; doi:10.1371/journal.pmed.1004329)
Supplement: S2 Table — S2A Table. Discomfort acceptability of IV, SC, and IM infusion. S2B Table. Pain acceptability of IV, SC, and IM infusion. S2C Table. Anxiety acceptability of IV, SC, and IM infusion. S2D Table. Amount of time acceptability of IV, SC, and IM infusion. S2E Table. Willingness to use of IV, SC, and IM infusion in general population. S2F Table. Recommendations for use of IV, SC, and IM infusion in general population. (PDF) [file pmed.1004329.s003.pdf]

Supplemental Table 2A: Discomfort acceptability of IV, SC, and IM infusion

Question	Route	Visit	Visit description	No discomfort	Discomfort acceptable	Discomfort not acceptable
Level of discomfort from [IV infusion/SC injection(s)/IM injection(s)]	IV	Day 0	1st SPA	52/59 88.1%	6/59 10.2%	1/59 1.7%
		Month 4	2nd SPA	46/55 83.6%	9/55 16.4%	0/55 0.0%
		Month 8	3rd SPA	43/51 84.3%	8/51 15.7%	0/51 0.0%
		Month 12	4th SPA	41/49 83.7%	8/49 16.3%	0/49 0.0%
		Month 16	5th SPA	38/44 86.4%	6/44 13.6%	0/44 0.0%
	SC	Day 0	1st SPA	22/41 53.7%	18/41 43.9%	1/41 2.4%
		Month 4	2nd SPA	13/40 32.5%	26/40 65.0%	1/40 2.5%
		Month 8	3rd SPA	19/37 51.4%	18/37 48.6%	0/37 0.0%
		Month 12	4th SPA	14/33 42.4%	18/33 54.5%	1/33 3.0%
		Month 16	5th SPA	15/31 48.4%	15/31 48.4%	1/31 3.2%
	IM	Day 0	1st SPA	16/24 66.7%	8/24 33.3%	0/24 0.0%
		Month 4	2nd SPA	12/23 52.2%	11/23 47.8%	0/23 0.0%
		Month 8	3rd SPA	15/23 65.2%	8/23 34.8%	0/23 0.0%
		Month 12	4th SPA	14/20 70.0%	6/20 30.0%	0/20 0.0%
		Month 16	5th SPA	14/20 70.0%	6/20 30.0%	0/20 0.0%

Supplemental Table 2B: Pain acceptability of IV, SC, and IM infusion

Question	Route	Visit	Visit description	No pain	Pain acceptable	Pain not acceptable
Level of pain from [IV infusion/SC injection(s)/IM injection(s)]	IV	Day 0	1st SPA	54/59 91.5%	5/59 8.5%	0/59 0.0%
		Month 4	2nd SPA	50/55 90.9%	4/55 7.3%	1/55 1.8%
		Month 8	3rd SPA	46/51 90.2%	5/51 9.8%	0/51 0.0%
		Month 12	4th SPA	45/49 91.8%	4/49 8.2%	0/49 0.0%
		Month 16	5th SPA	40/44 90.9%	4/44 9.1%	0/44 0.0%
	SC	Day 0	1st SPA	20/41 48.8%	20/41 48.8%	1/41 2.4%
		Month 4	2nd SPA	21/40 52.5%	18/40 45.0%	1/40 2.5%
		Month 8	3rd SPA	20/37 54.1%	16/37 43.2%	1/37 2.7%
		Month 12	4th SPA	14/33 42.4%	17/33 51.5%	2/33 6.1%
		Month 16	5th SPA	17/31 54.8%	13/31 41.9%	1/31 3.2%
	IM	Day 0	1st SPA	12/24 50.0%	12/24 50.0%	0/24 0.0%
		Month 4	2nd SPA	12/23 52.2%	10/23 43.5%	1/23 4.3%
		Month 8	3rd SPA	12/23 52.2%	11/23 47.8%	0/23 0.0%
		Month 12	4th SPA	11/20 55.0%	9/20 45.0%	0/20 0.0%
		Month 16	5th SPA	14/20 70.0%	6/20 30.0%	0/20 0.0%

Supplemental Table 2C: Anxiety acceptability of IV, SC, and IM infusion

Question	Route	Visit	Visit description	No anxiety	Anxiety acceptable	Anxiety not acceptable
Level of anxiety from [IV infusion/SC injection(s)/IM injection(s)]	IV	Day 0	1st SPA	56/59 94.9%	3/59 5.1%	0/59 0.0%
		Month 4	2nd SPA	51/55 92.7%	4/55 7.3%	0/55 0.0%
		Month 8	3rd SPA	46/51 90.2%	5/51 9.8%	0/51 0.0%
		Month 12	4th SPA	45/49 91.8%	4/49 8.2%	0/49 0.0%
		Month 16	5th SPA	43/44 97.7%	1/44 2.3%	0/44 0.0%
	SC	Day 0	1st SPA	30/41 73.2%	11/41 26.8%	0/41 0.0%
		Month 4	2nd SPA	35/40 87.5%	5/40 12.5%	0/40 0.0%
		Month 8	3rd SPA	35/37 94.6%	2/37 5.4%	0/37 0.0%
		Month 12	4th SPA	29/33 87.9%	3/33 9.1%	1/33 3.0%
		Month 16	5th SPA	27/31 87.1%	4/31 12.9%	0/31 0.0%
	IM	Day 0	1st SPA	19/24 79.2%	5/24 20.8%	0/24 0.0%
		Month 4	2nd SPA	20/23 87.0%	3/23 13.0%	0/23 0.0%
		Month 8	3rd SPA	23/23 100.0%	0/23 0.0%	0/23 0.0%
		Month 12	4th SPA	18/20 90.0%	2/20 10.0%	0/20 0.0%
		Month 16	5th SPA	17/20 85.0%	3/20 15.0%	0/20 0.0%

Supplemental Table 2D: Amount of time acceptability of IV, SC, and IM infusion

Question	Route	Visit	Visit description	Amount of time acceptable	Amount of time not acceptable
The amount of time required to spend in the clinic during the [IV infusion/SC injection(s)/IM injection(s)]	IV	Day 0	1st SPA	54/59 91.5%	5/59 8.5%
		Month 4	2nd SPA	54/55 98.2%	1/55 1.8%
		Month 8	3rd SPA	48/51 94.1%	3/51 5.9%
		Month 12	4th SPA	47/49 95.9%	2/49 4.1%
		Month 16	5th SPA	43/44 97.7%	1/44 2.3%
	SC	Day 0	1st SPA	41/41 100.0%	0/41 0.0%
		Month 4	2nd SPA	39/40 97.5%	1/40 2.5%
		Month 8	3rd SPA	35/37 94.6%	2/37 5.4%
		Month 12	4th SPA	29/33 87.9%	4/33 12.1%
		Month 16	5th SPA	27/31 87.1%	4/31 12.9%
	IM	Day 0	1st SPA	22/24 91.7%	2/24 8.3%
		Month 4	2nd SPA	23/23 100.0%	0/23 0.0%
		Month 8	3rd SPA	23/23 100.0%	0/23 0.0%
		Month 12	4th SPA	20/20 100.0%	0/20 0.0%
		Month 16	5th SPA	20/20 100.0%	0/20 0.0%

Supplemental Table 2E: Willingness to use of IV, SC, and IM infusion in general population

Question	Route	Visit	Visit description	Very willing	Somewhat willing	Not willing
How willing would you be to get [IV infusions/SC injections/IM injection(s)] to prevent you from getting a serious disease, such as HIV, if you were at risk for that disease?	IV	Day 0	1st SPA	47/59 79.7%	12/59 20.3%	0/59 0.0%
		Month 4	2nd SPA	46/55 83.6%	9/55 16.4%	0/55 0.0%
		Month 8	3rd SPA	41/51 80.4%	10/51 19.6%	0/51 0.0%
		Month 12	4th SPA	41/49 83.7%	8/49 16.3%	0/49 0.0%
		Month 16	5th SPA	37/44 84.1%	7/44 15.9%	0/44 0.0%
	SC	Day 0	1st SPA	35/41 85.4%	5/41 12.2%	1/41 2.4%
		Month 4	2nd SPA	32/40 80.0%	8/40 20.0%	0/40 0.0%
		Month 8	3rd SPA	32/37 86.5%	5/37 13.5%	0/37 0.0%
		Month 12	4th SPA	27/33 81.8%	6/33 18.2%	0/33 0.0%
		Month 16	5th SPA	24/31 77.4%	6/31 19.4%	1/31 3.2%
	IM	Day 0	1st SPA	22/24 91.7%	2/24 8.3%	0/24 0.0%
		Month 4	2nd SPA	22/23 95.7%	1/23 4.3%	0/23 0.0%
		Month 8	3rd SPA	22/23 95.7%	1/23 4.3%	0/23 0.0%
		Month 12	4th SPA	20/20 100.0%	0/20 0.0%	0/20 0.0%
		Month 16	5th SPA	20/20 100.0%	0/20 0.0%	0/20 0.0%

Supplemental Table 2F: Recommendations for use of IV, SC, and IM infusion in general population

Question	Route	Visit	Visit description	Yes	No	Don't know
Would you recommend receiving the [IV infusion/SC injection(s)/IM injection(s)] to a friend who is at risk for HIV?	IV	Day 0	1st SPA	54/59 91.5%	1/59 1.7%	4/59 6.8%
		Month 4	2nd SPA	52/55 94.5%	0/55 0.0%	3/55 5.5%
		Month 8	3rd SPA	49/51 96.1%	0/51 0.0%	2/51 3.9%
		Month 12	4th SPA	47/49 95.9%	1/49 2.0%	1/49 2.0%
		Month 16	5th SPA	43/44 97.7%	0/44 0.0%	1/44 2.3%
	SC	Day 0	1st SPA	40/41 97.6%	1/41 2.4%	0/41 0.0%
		Month 4	2nd SPA	38/40 95.0%	1/40 2.5%	1/40 2.5%
		Month 8	3rd SPA	34/37 91.9%	0/37 0.0%	3/37 8.1%
		Month 12	4th SPA	30/33 90.9%	1/33 3.0%	2/33 6.1%
		Month 16	5th SPA	24/31 77.4%	3/31 9.7%	4/31 12.9%
	IM	Day 0	1st SPA	24/24 100.0%	0/24 0.0%	0/24 0.0%
		Month 4	2nd SPA	23/23 100.0%	0/23 0.0%	0/23 0.0%
		Month 8	3rd SPA	23/23 100.0%	0/23 0.0%	0/23 0.0%
		Month 12	4th SPA	20/20 100.0%	0/20 0.0%	0/20 0.0%
		Month 16	5th SPA	19/20 95.0%	0/20 0.0%	1/20 5.0%
