# Supplementary material for: Safety and pharmacokinetics of VRC07-523LS administered via different routes and doses (HVTN 127/HPTN 087): A Phase I randomized clinical trial
Source: PLoS Med. 2024 Jun 24;21(6):e1004329. doi: 10.1371/journal.pmed.1004329 (PMC11251612; doi:10.1371/journal.pmed.1004329)
Supplement: S3 Table — The model assumed a linearly combined error variance and a fully unstructured random effects variance-covariance matrix. SE, standard error; %RSE, relative SE, calculated as (SE/Estimate) × 100. (PDF) [file pmed.1004329.s004.pdf]

Parameter	Units	Estimate	SE	%RSE
Bioavailability for SC administration (F_{SC})	-	0.40	1.3e-03	0.32
Bioavailability for IM administration (F_{IM})	-	0.57	0.03	4.67
Absorption rate constant for SC administration (Ka_{SC})	1/day	0.30	0.01	4.92
Absorption rate constant for IM administration (Ka_{IM})	1/day	0.39	0.02	4.04
ELISA elimination clearance (Cl)	L/day	0.11	3.1e-03	2.75
Increase in elimination clearance from BAMA relative to ELISA (β_{Cl})	L/day	0.04	1.2e-03	3.21
ELISA central compartment volume (V1)	L	2.72	0.12	4.25
Increase in central compartment volume from BAMA relative to ELISA (β_{V1})	L	0.46	0.03	6.04
ELISA intercompartmental clearance (Q)	L/day	0.49	0.03	6.68
Increase in intercompartmental clearance volume from BAMA relative to ELISA (β_{Q})	L/day	0.69	0.04	6.08
ELISA peripheral compartment volume (V2)	L	3.59	0.12	3.35
Increase in peripheral compartment volume from BAMA relative to ELISA (β_{V2})	L	0.15	2.9e-03	1.93
Standard deviation for random effect Cl	-	0.28	0.02	6.83
Standard deviation for random effect V1	-	0.33	0.03	9.72
Standard deviation for random effect Q	-	0.54	0.05	9.95
Standard deviation for random effect V2	-	0.23	0.03	11.08
Correlation between Q and Cl	-	0.15	0.12	82.95
Correlation between V1 and Cl	-	0.43	0.10	22.97
Correlation between V2 and Cl	-	1.00	0.02	1.84
Correlation between V1 and Q	-	-0.33	0.13	38.27
Correlation between V2 and Q	-	0.24	0.15	60.14
Correlation between V2 and V1	-	0.39	0.14	36.54
Error model (intercept)	-	0.19	0.02	9.65
Error model (slope)	-	0.14	4.6e-03	3.21
ELISA distribution half-life	day	1.17	0.13	10.72
Increase in distribution half-life from BAMA relative to ELISA (β_{DHL})	day	3.44	0.03	0.92
ELISA elimination half-life	day	42.43	0.75	1.77
Increase in elimination half-life from BAMA relative to ELISA (β_{EHL})	day	0.24	2.2e-03	0.90

T1: IV 2.5 mg/kg VRC07-523LS
T2: IV 5 mg/kg VRC07-523LS
T3: IV 20 mg/kg VRC07-523LS
T4: SC 2.5 mg/kg VRC07-523LS
T5: SC 5 mg/kg VRC07-523LS
T6: IM 2.5 mg/kg VRC0-7523LS

Supplemental Table 3: Parameter estimates based on the two-compartment population PK model fitted to VRC07-523LS serum concentrations in participants who received the study product via the IV (T1-T3), SC (T4, T5), or IM (T6) route. The model assumed a linearly combined error variance and a fully unstructured random effects variance-covariance matrix. SE: standard error; %RSE: relative SE, calculated as (SE/Estimate) × 100.
