# Supplementary material for: Safety and pharmacokinetics of VRC07-523LS administered via different routes and doses (HVTN 127/HPTN 087): A Phase I randomized clinical trial
Source: PLoS Med. 2024 Jun 24;21(6):e1004329. doi: 10.1371/journal.pmed.1004329 (PMC11251612; doi:10.1371/journal.pmed.1004329)
Supplement: S4 Table — (PDF) [file pmed.1004329.s005.pdf]

Supplemental Table 4. Pharmacokinetic parameter covariate analysis

PK parameter	covariate	Spearman rho	p-value	BH adjusted p-value
Cl	weight	0.55	<0.001	<0.001
Cl	BMI	0.44	<0.001	<0.001
Cl	age	0.15	0.092	0.14
V1	weight	0.34	<0.001	<0.001
V1	BMI	0.28	0.0018	0.0035
V1	age	0.03	0.76	0.91
Q	weight	-0.01	0.87	0.95
Q	BMI	-0.05	0.62	0.83
Q	age	0	0.99	0.99
V2	weight	0.54	<0.001	<0.001
V2	BMI	0.43	<0.001	<0.001
V2	age	0.16	0.087	0.14
