# Supplementary material for: Safety and pharmacokinetics of VRC07-523LS administered via different routes and doses (HVTN 127/HPTN 087): A Phase I randomized clinical trial
Source: PLoS Med. 2024 Jun 24;21(6):e1004329. doi: 10.1371/journal.pmed.1004329 (PMC11251612; doi:10.1371/journal.pmed.1004329)
Supplement: S1 Fig — Individual measurements are shown in Panel A; note X-axis is not to scale. Numbers at the top of the panels indicate the sample size at each time point. Longitudinal per-participant levels are shown in Panel B. (PDF) [file pmed.1004329.s006.pdf]

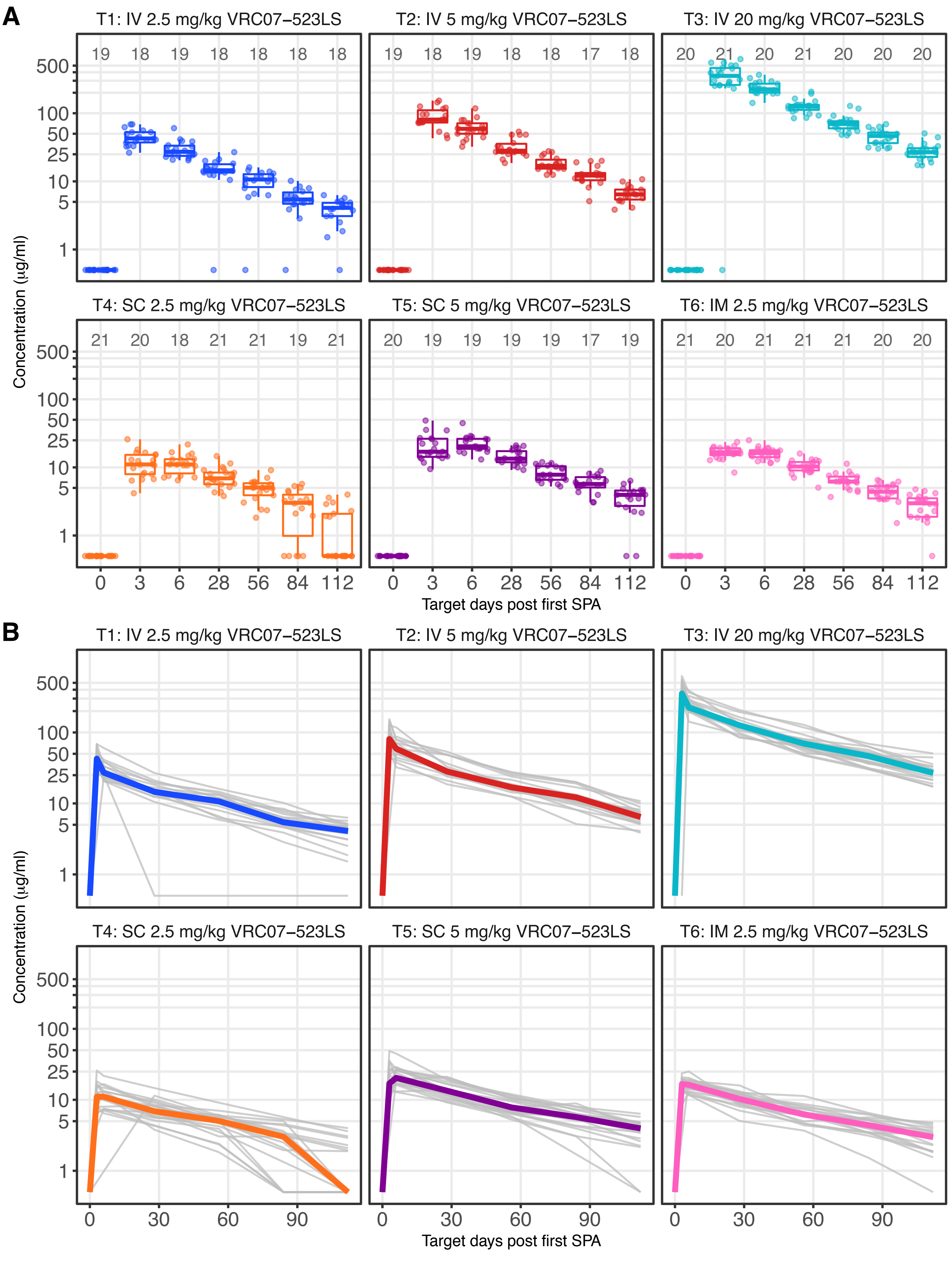

Supplemental Figure 1. VRC07-523LS levels following first study product administration (SPA). Individual measurements are shown in Panel **A**; note X-axis is not to scale. Numbers at the top of the panels indicate the sample size at each time point. Longitudinal per-participant levels are shown in Panel **B**.
