# Supplementary material for: Safety and pharmacokinetics of VRC07-523LS administered via different routes and doses (HVTN 127/HPTN 087): A Phase I randomized clinical trial
Source: PLoS Med. 2024 Jun 24;21(6):e1004329. doi: 10.1371/journal.pmed.1004329 (PMC11251612; doi:10.1371/journal.pmed.1004329)
Supplement: S2 Fig — Numbers at the top of the panels indicate the sample size at each time point. (PDF) [file pmed.1004329.s007.pdf]

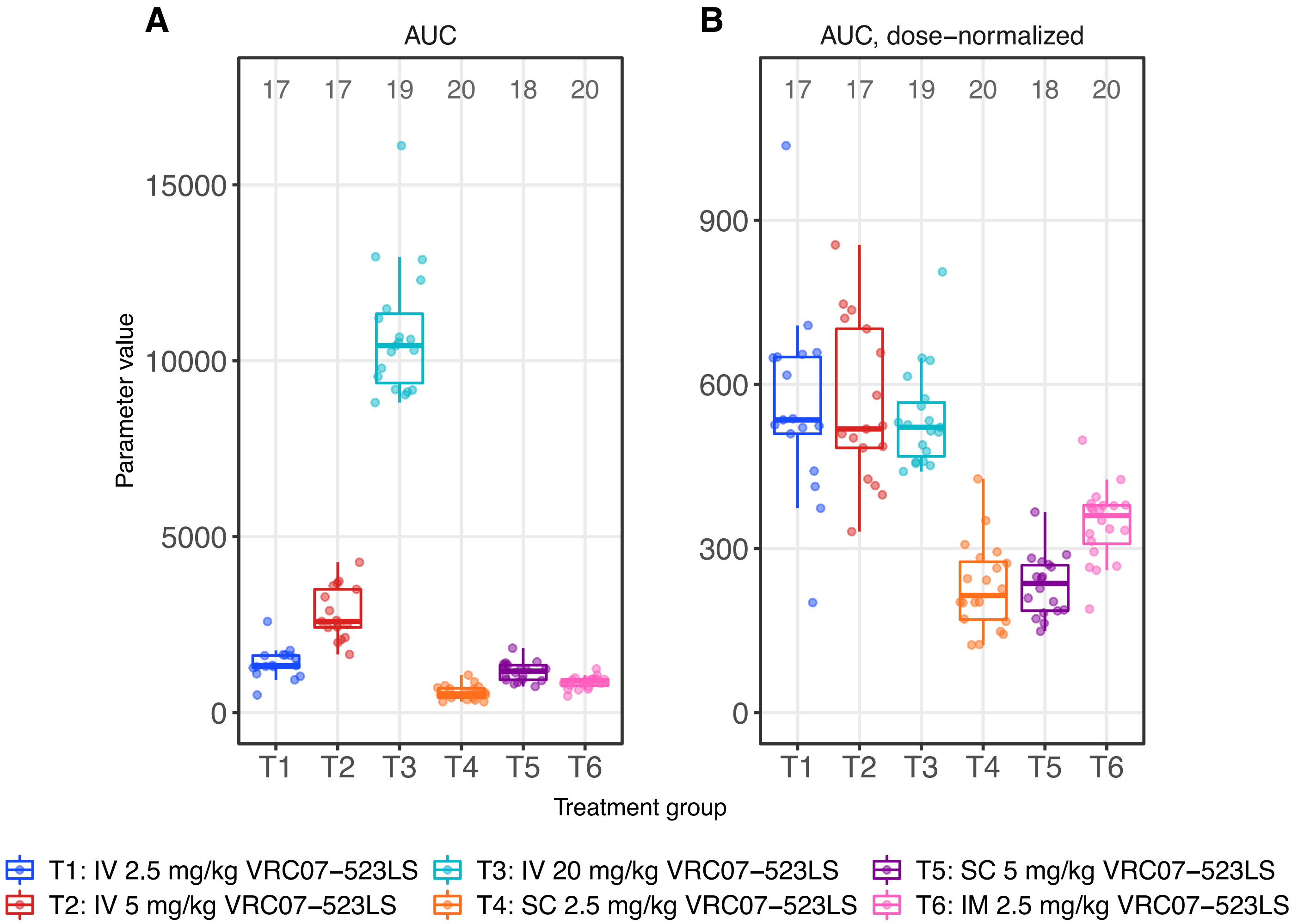

Supplemental Figure 2. VRC07-523LS serum concentrations were measured following the first study product administration and the area under the curve (AUC, panel **A**) and AUC corrected for dose (**B**) were computed to characterize the pharmacokinetics of VRC07-523LS. Numbers at the top of the panels indicate the sample size at each time point.
