# Supplementary material for: Safety and pharmacokinetics of VRC07-523LS administered via different routes and doses (HVTN 127/HPTN 087): A Phase I randomized clinical trial
Source: PLoS Med. 2024 Jun 24;21(6):e1004329. doi: 10.1371/journal.pmed.1004329 (PMC11251612; doi:10.1371/journal.pmed.1004329)
Supplement: S3 Fig — Peak levels were only assessed after the first dose. Levels following the second and subsequent doses were assessed by binding antigen multiplex assay (BAMA). Individual-level data are shown in gray, and the group median is shown in color. (PDF) [file pmed.1004329.s008.pdf]

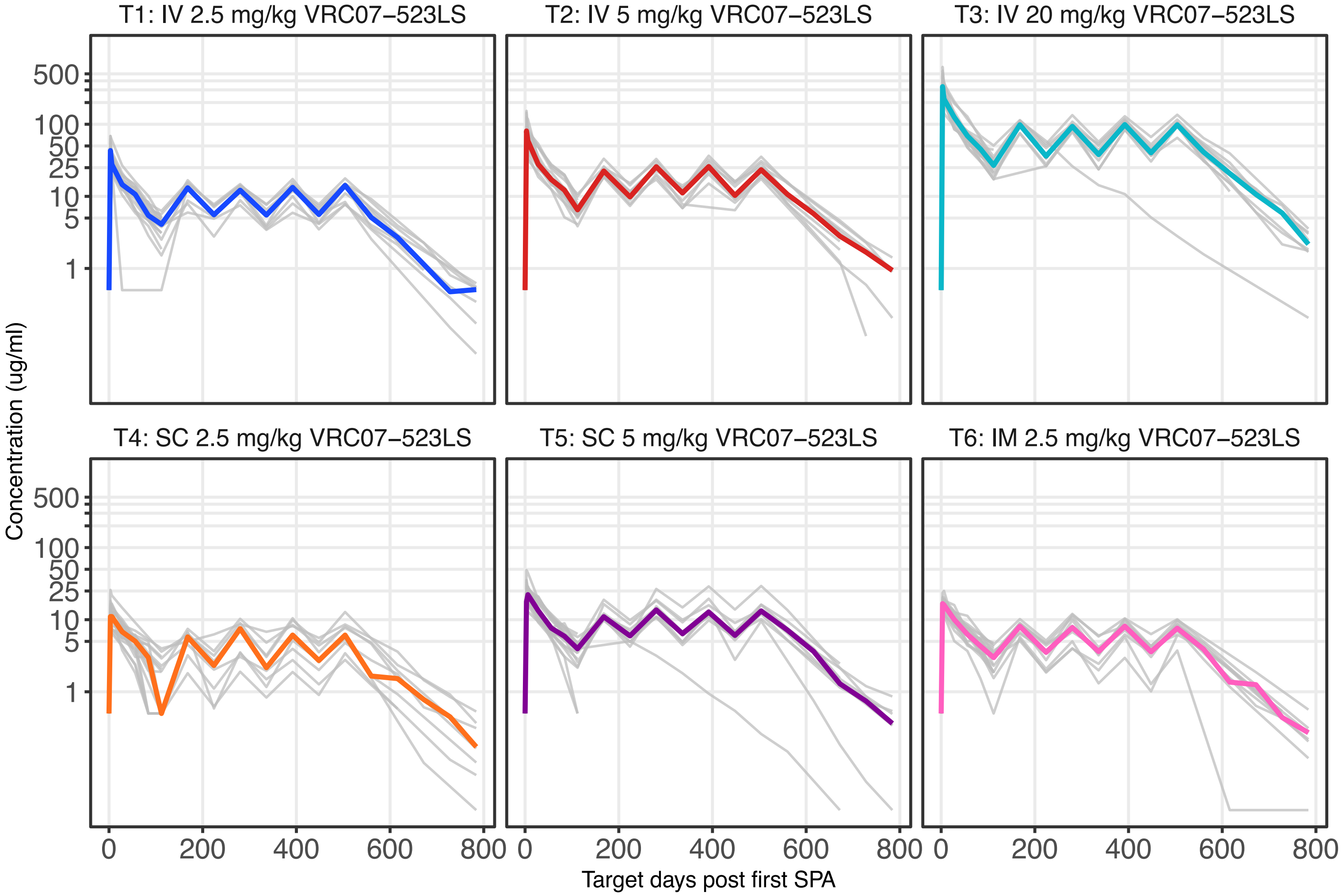

Supplemental Figure 3. VRC07-523LS concentrations measured after product administration every four months at specified doses and routes. Peak levels were only assessed after the first dose. Levels following the the second and subsequent doses were assessed by binding antigen multiplex assay (BAMA). Individual-level data are shown in grey and the group median is shown in colour.
