# Supplementary material for: Safety and pharmacokinetics of VRC07-523LS administered via different routes and doses (HVTN 127/HPTN 087): A Phase I randomized clinical trial
Source: PLoS Med. 2024 Jun 24;21(6):e1004329. doi: 10.1371/journal.pmed.1004329 (PMC11251612; doi:10.1371/journal.pmed.1004329)
Supplement: S4 Fig — S4A Fig. Group 1 (IV 2.5 mg/kg) is shown. S4B Fig. Group 2 (IV 5 mg/kg) is shown. S4C Fig. Group 3 (IV 20 mg/kg) is shown. S4D Fig. Group 4 (SC 2.5 mg/kg) is shown. S4E Fig. Group 5 (SC 5 mg/kg) is shown. S4F Fig. Group 6 (IM 2.5 mg/kg) is shown. The 2-compartment population PK model with fully unstructured random effects variance−covariance matrix was fitted to VRC07−523LS concentrations. (PDF) [file pmed.1004329.s009.pdf]

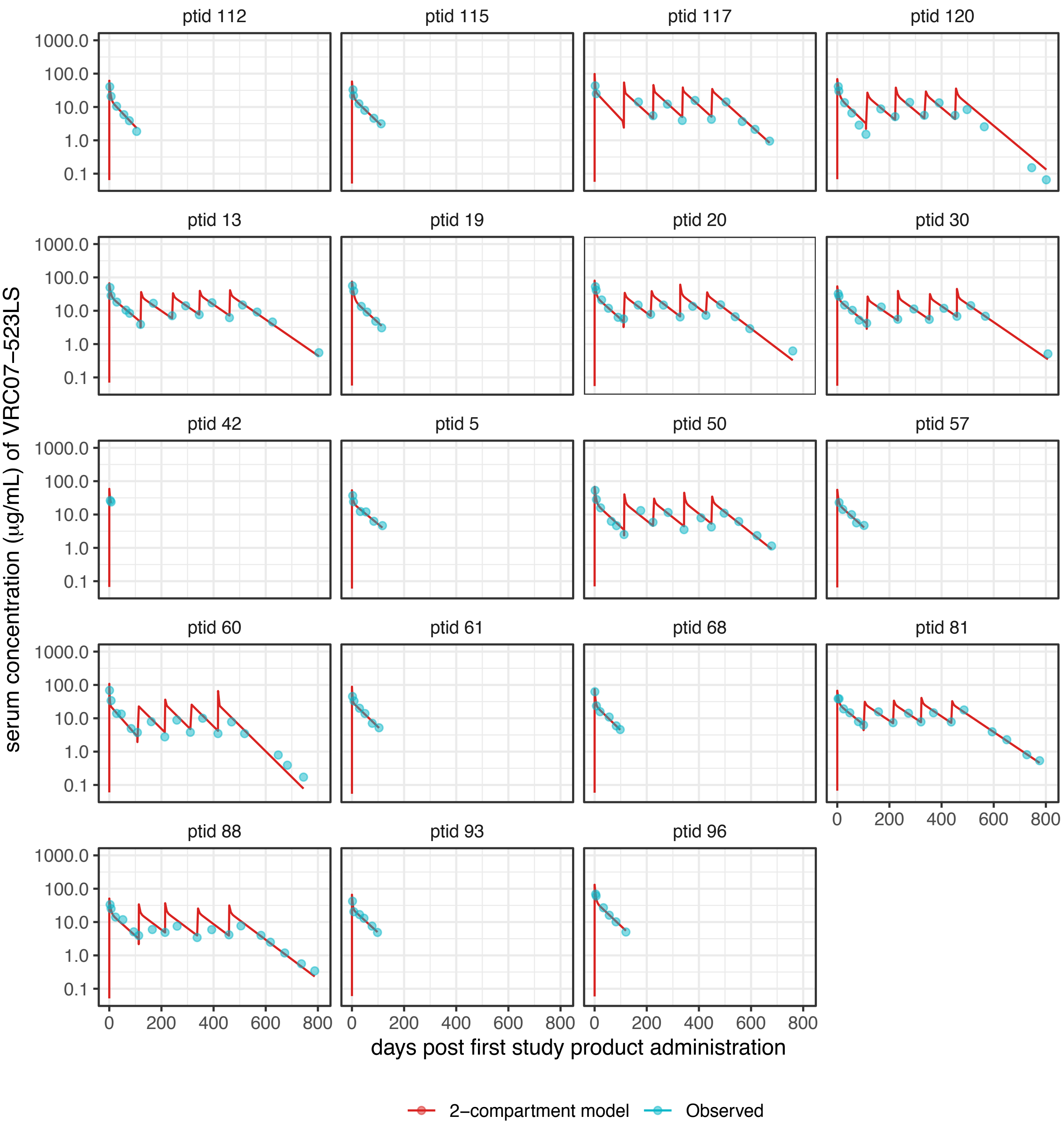

Supplemental Figure 4A. Observed (·) and predicted (–) serum concentrations of VRC07−523LS as a function of time in individual participants (one per plot); Group 1 (IV 2.5 mg/kg) is shown. The two−compartment population PK model with fully unstructured random effects variance−covariance matrix was fitted to VRC07−523LS concentrations.

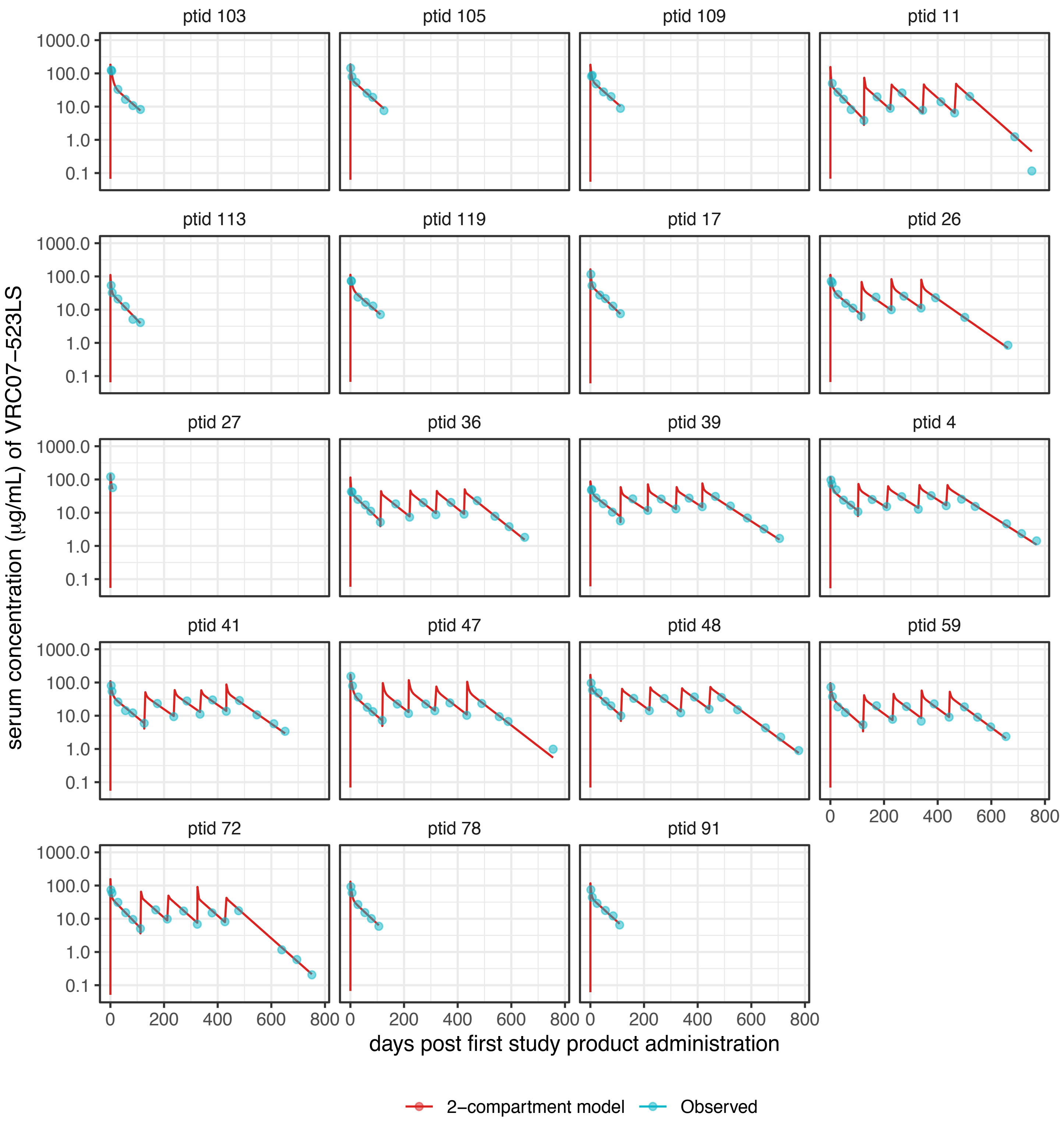

Supplemental Figure 4B. Observed (·) and predicted (–) serum concentrations of VRC07−523LS as a function of time in individual participants (one per plot); Group 2 (IV 5 mg/kg) is shown. The two−compartment population PK model with fully unstructured random effects variance−covariance matrix was fitted to VRC07−523LS concentrations.

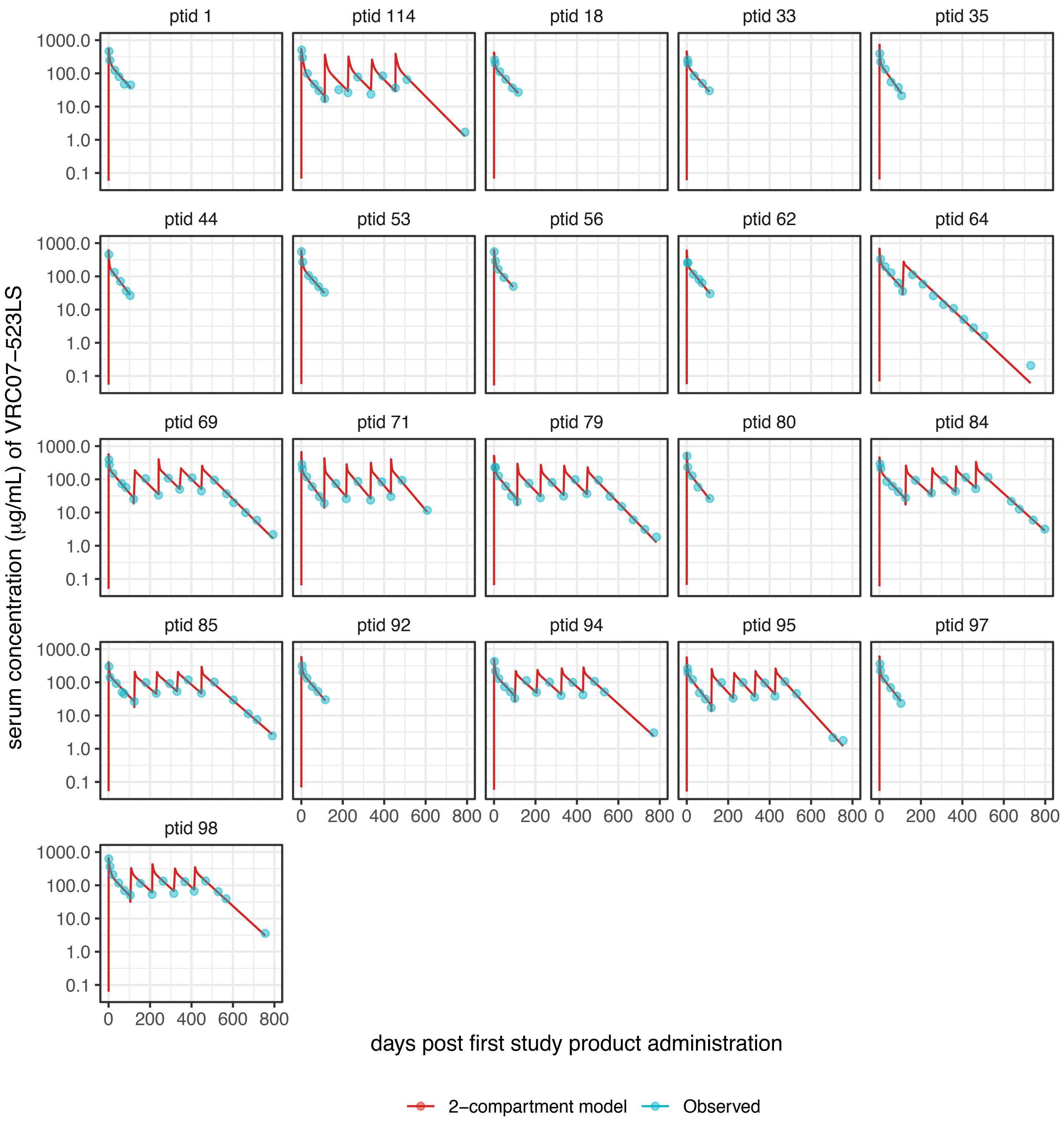

Supplemental Figure 4C. Observed (·) and predicted (–) serum concentrations of VRC07−523LS as a function of time in individual participants (one per plot); Group 3 (IV 20 mg/kg) is shown. The two−compartment population PK model with fully unstructured random effects variance−covariance matrix was fitted to VRC07−523LS concentrations.

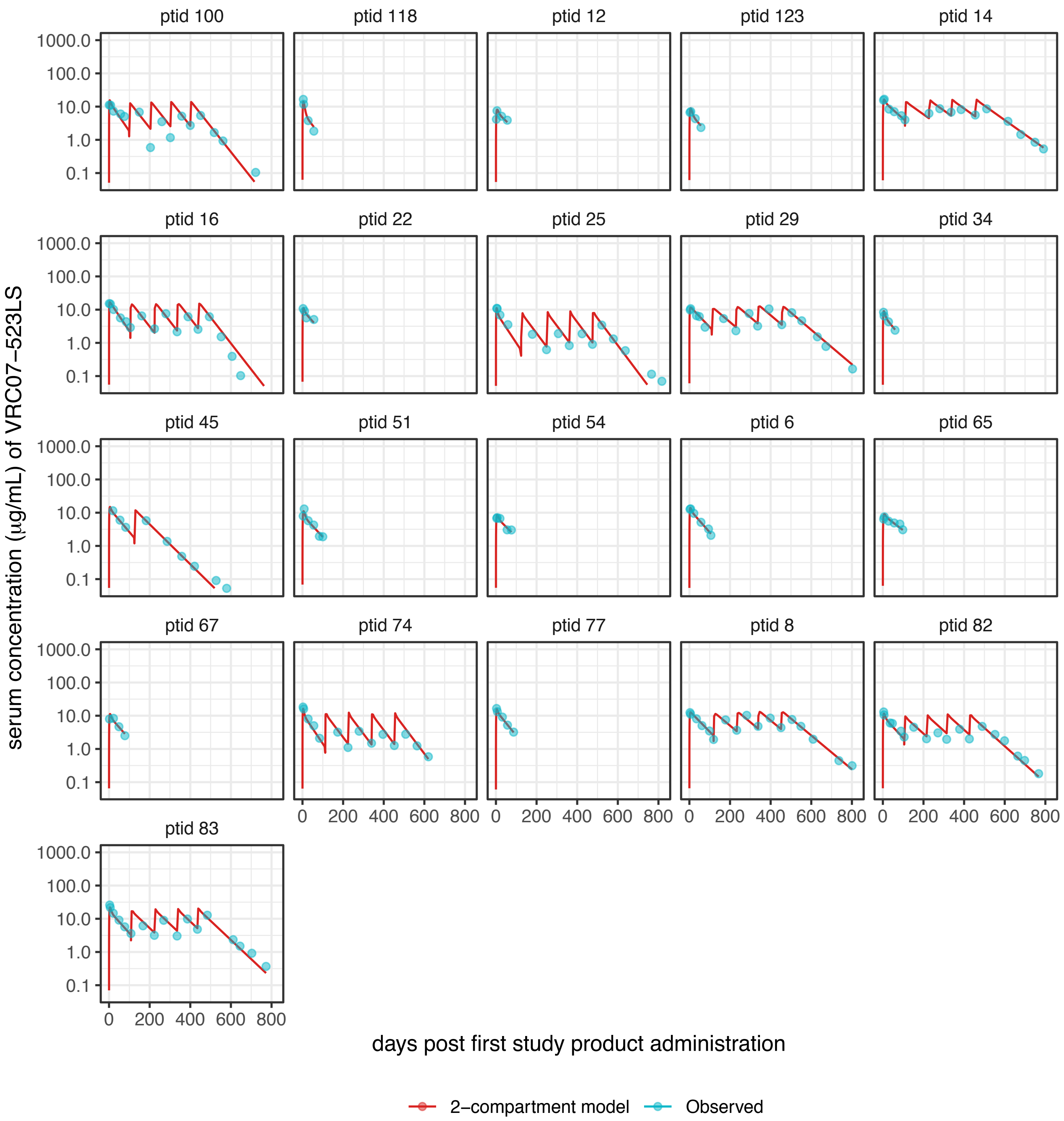

Supplemental Figure 4D. Observed (·) and predicted (–) serum concentrations of VRC07−523LS as a function of time in individual participants (one per plot); Group 4 (SC 2.5 mg/kg) is shown. The two−compartment population PK model with fully unstructured random effects variance−covariance matrix was fitted to VRC07−523LS concentrations.

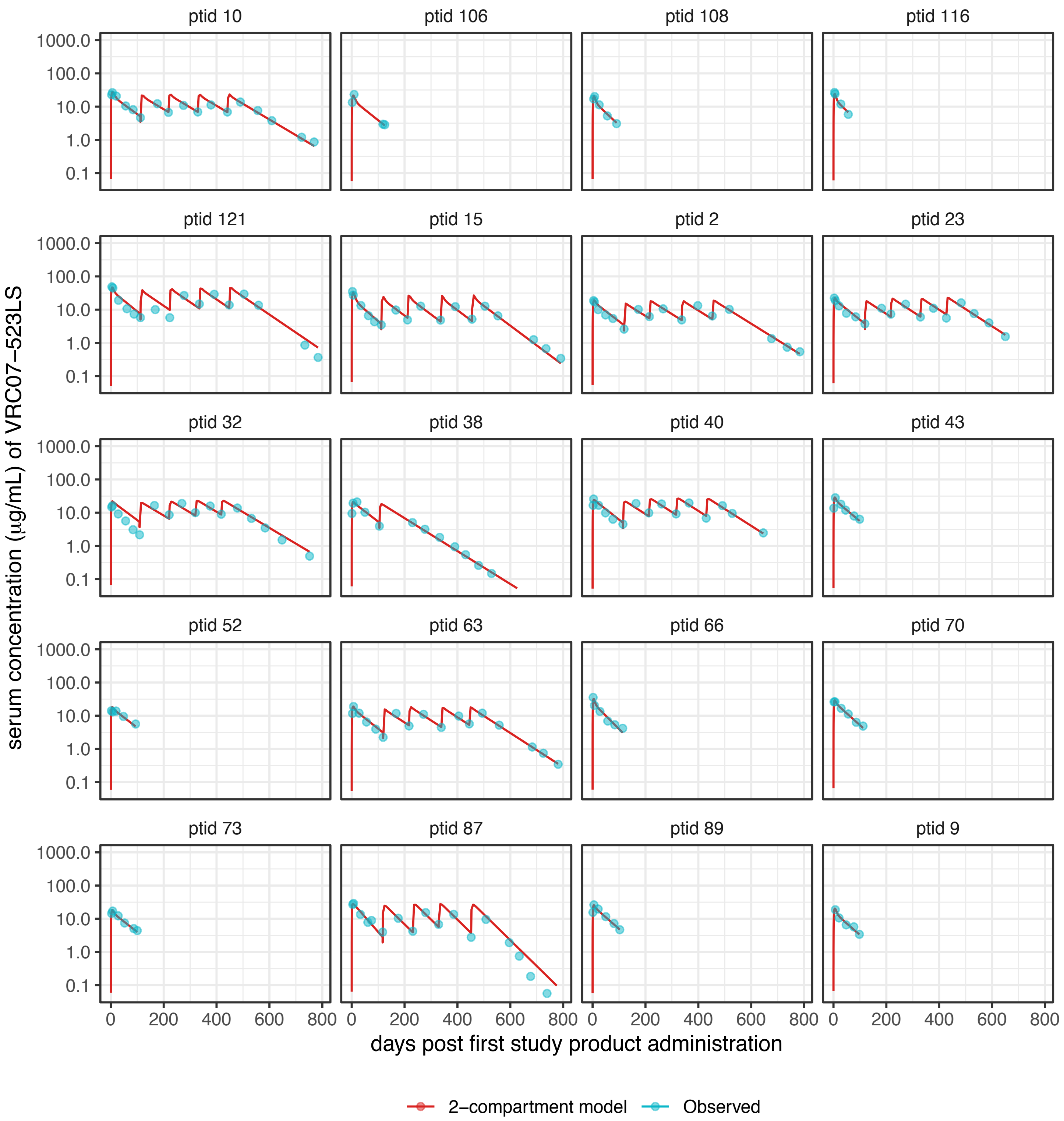

Supplemental Figure 4E. Observed (·) and predicted (–) serum concentrations of VRC07−523LS as a function of time in individual participants (one per plot); Group 5 (SC 5 mg/kg) is shown. The two−compartment population PK model with fully unstructured random effects variance−covariance matrix was fitted to VRC07−523LS concentrations.

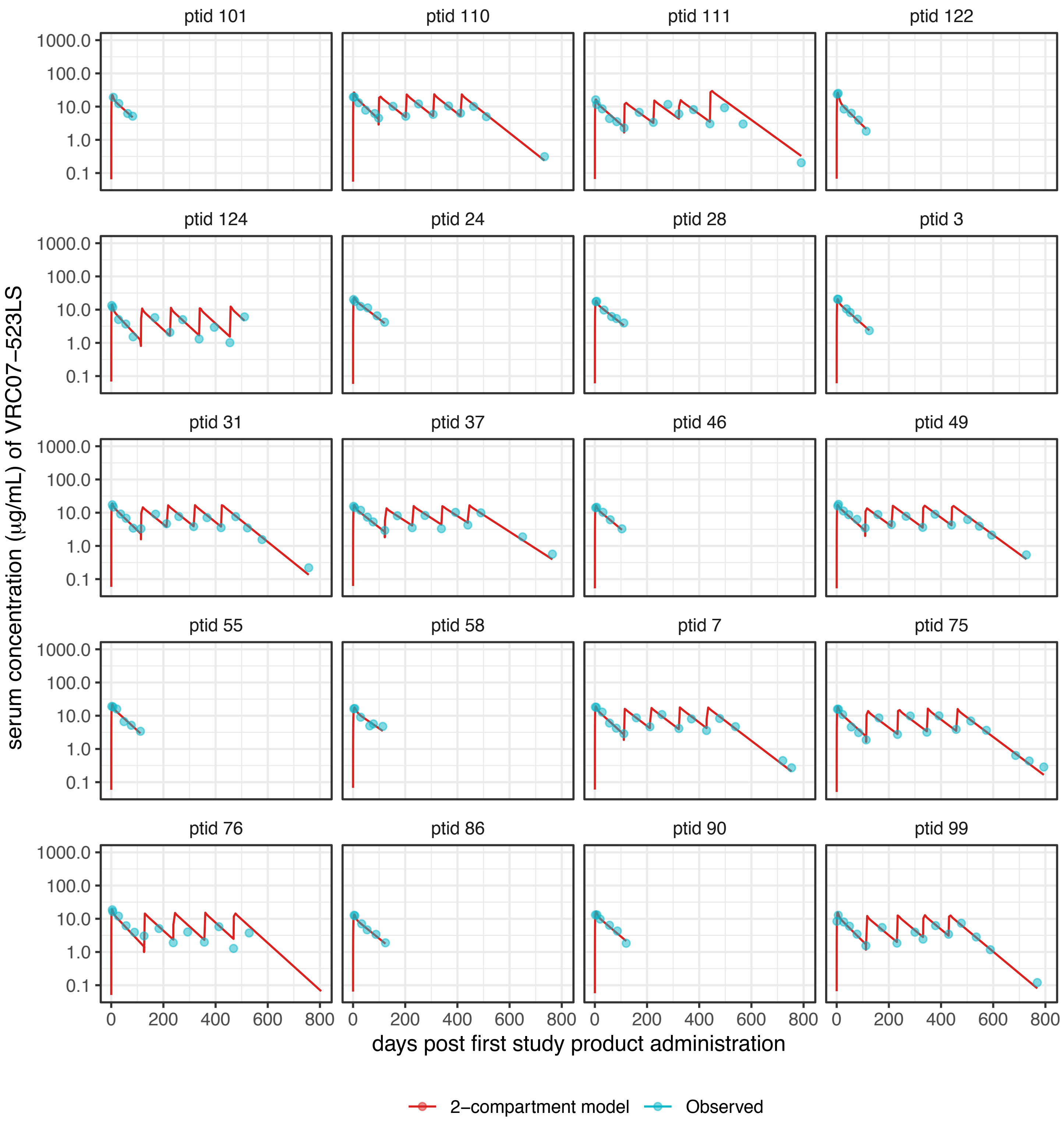

Supplemental Figure 4F. Observed (·) and predicted (–) serum concentrations of VRC07–523LS as a function of time in individual participants (one per plot); Group 6 (IM 2.5 mg/kg) is shown. The two–compartment population PK model with fully unstructured random effects variance–covariance matrix was fitted to VRC07–523LS concentrations.
