# Supplementary material for: Safety and pharmacokinetics of VRC07-523LS administered via different routes and doses (HVTN 127/HPTN 087): A Phase I randomized clinical trial
Source: PLoS Med. 2024 Jun 24;21(6):e1004329. doi: 10.1371/journal.pmed.1004329 (PMC11251612; doi:10.1371/journal.pmed.1004329)
Supplement: S5 Fig — Values were predicted from the 2-compartment model on a per-participant basis for each dose and route group. Box and whisker plots indicate the quartiles and median of the data set. (PDF) [file pmed.1004329.s010.pdf]

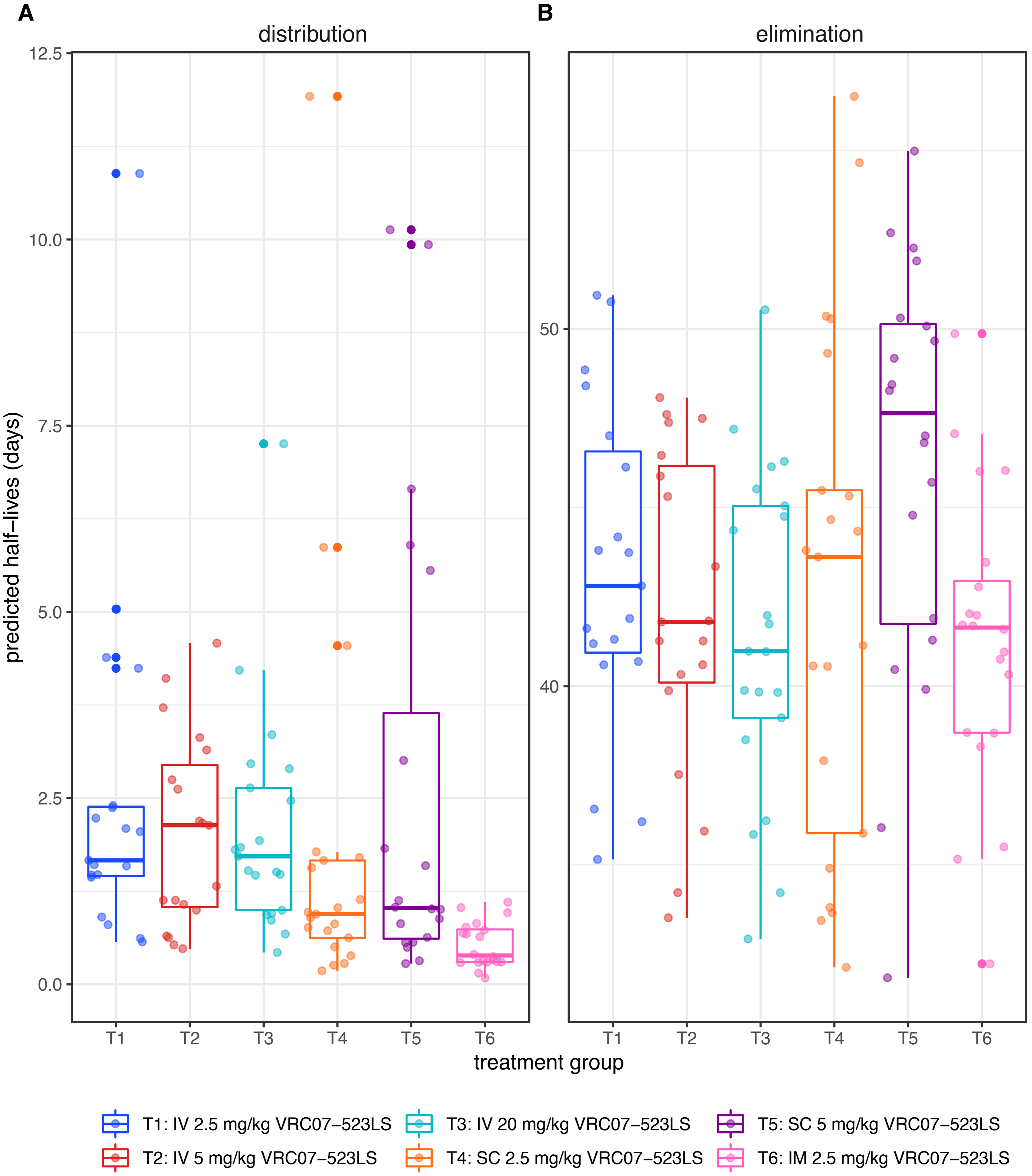

Supplemental Figure 5. Distribution (A) and elimination (B) half-life of VRC07-523LS. Values were predicted from the two-compartment model on a per-participant basis for each dose and route group. Box and whisker plots indicate the quartiles and median of the data set.
