# Supplementary material for: Safety and pharmacokinetics of VRC07-523LS administered via different routes and doses (HVTN 127/HPTN 087): A Phase I randomized clinical trial
Source: PLoS Med. 2024 Jun 24;21(6):e1004329. doi: 10.1371/journal.pmed.1004329 (PMC11251612; doi:10.1371/journal.pmed.1004329)
Supplement: S6 Fig — ID50 titer is shown. (PDF) [file pmed.1004329.s011.pdf]

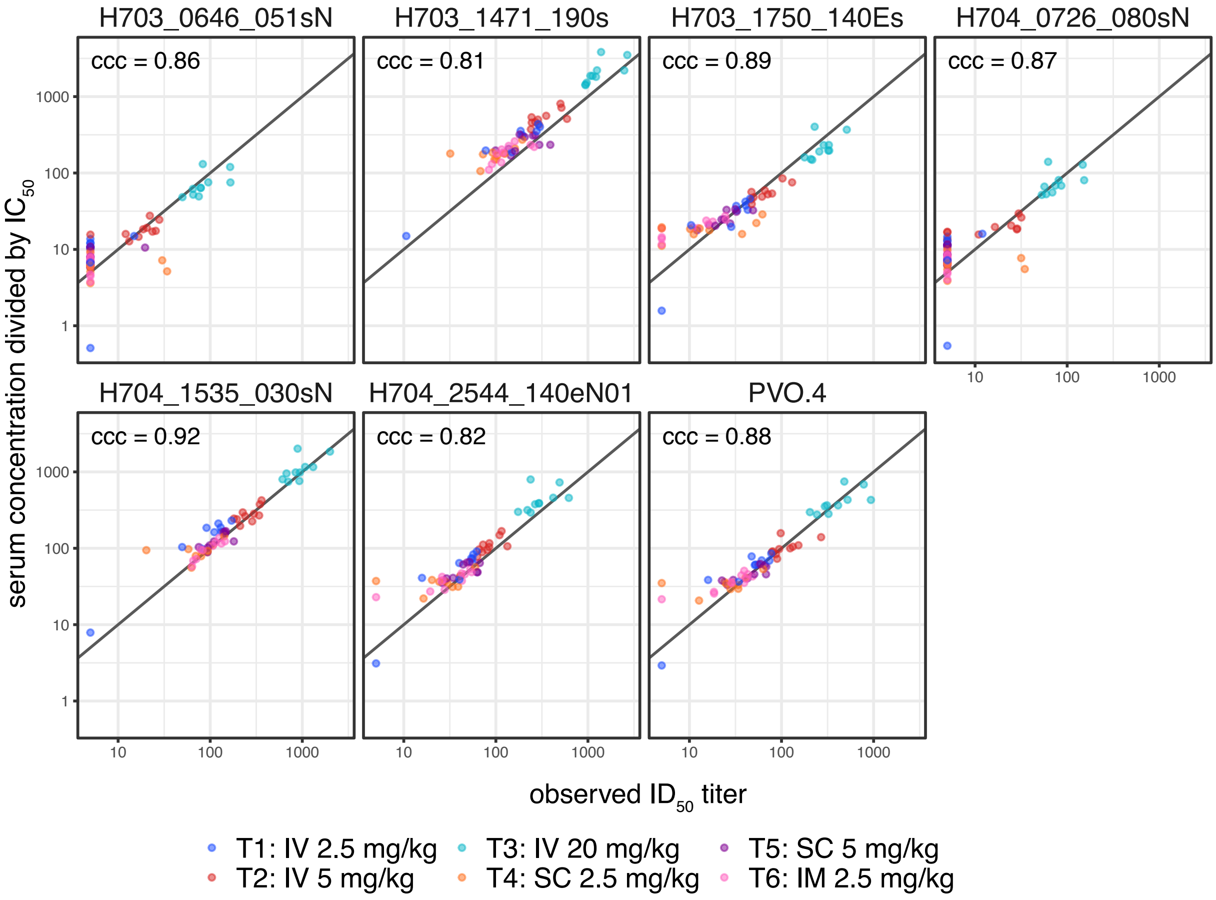

Supplemental Figure 6. Neutralization activity of participant serum 8 weeks following their first VRC07-523LS administration against seven HIV-1 isolates collected from incident HIV-1 acquisition events in placebo recipients in the AMP trials. ID_{50} titre is shown.
