# Supplementary material for: Safety and pharmacokinetics of VRC07-523LS administered via different routes and doses (HVTN 127/HPTN 087): A Phase I randomized clinical trial
Source: PLoS Med. 2024 Jun 24;21(6):e1004329. doi: 10.1371/journal.pmed.1004329 (PMC11251612; doi:10.1371/journal.pmed.1004329)
Supplement: S7 Fig — ID50 titer is shown at Week 8 (A) and Week 72 (B) following first study product administration. (PDF) [file pmed.1004329.s012.pdf]

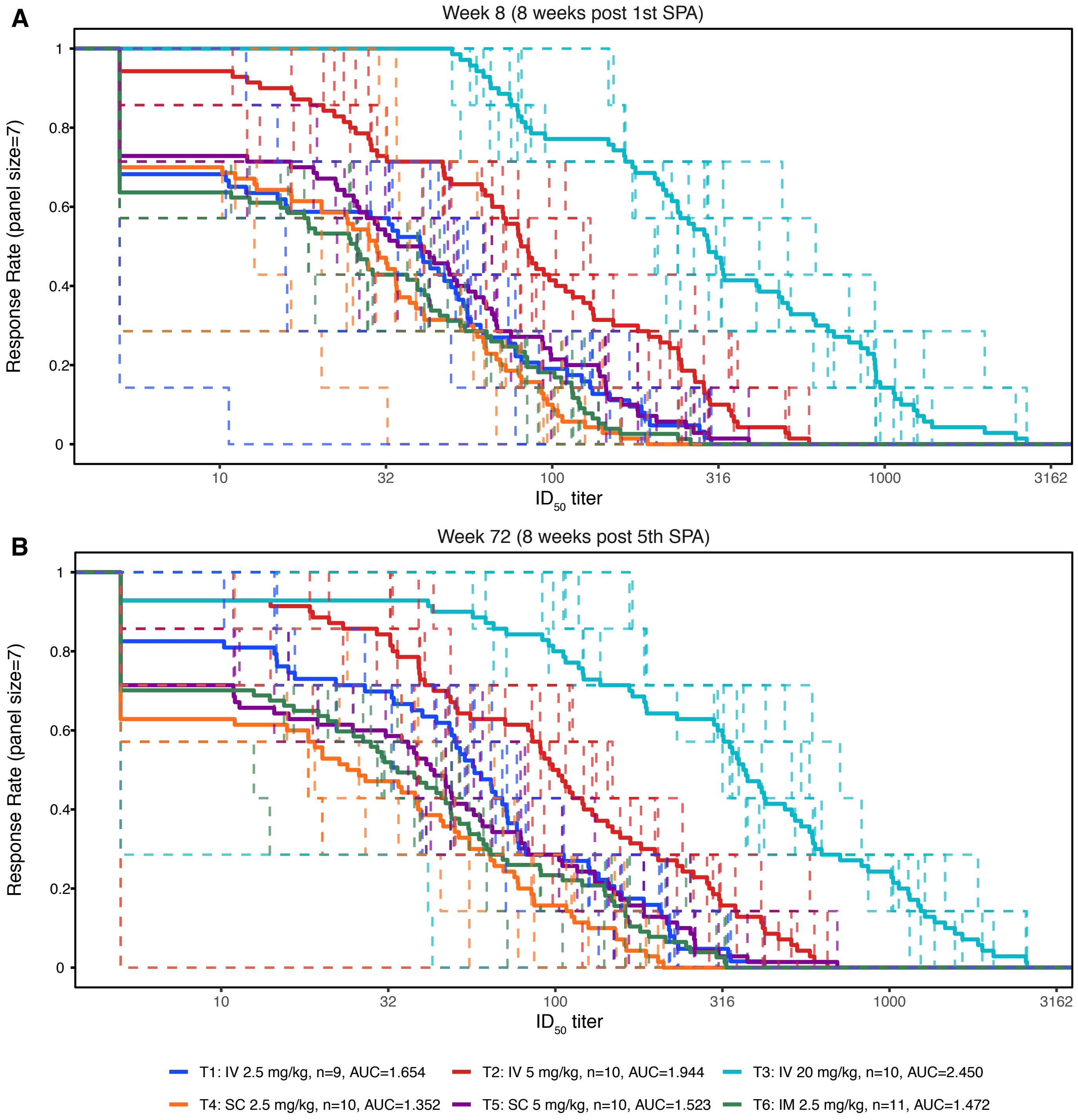

Supplemental Figure 7. Magnitude-breadth curves for participant serum following VRC07-523LS administration against a panel of HIV-1 isolates collected from incident HIV-1 acquisition events in placebo recipients in the AMP trials. ID_{50} titre is shown at Week 8 (**A**) and Week 72 (**B**) following first study product administration.
