# Supplementary material for: Safety and pharmacokinetics of VRC07-523LS administered via different routes and doses (HVTN 127/HPTN 087): A Phase I randomized clinical trial
Source: PLoS Med. 2024 Jun 24;21(6):e1004329. doi: 10.1371/journal.pmed.1004329 (PMC11251612; doi:10.1371/journal.pmed.1004329)
Supplement: S1 Protocol — (PDF) [file pmed.1004329.s013.pdf]

PROTOCOL

HVTN 127/HPTN 087

A multicenter, randomized, partially blinded phase 1 clinical trial to evaluate the safety and serum concentrations of a human monoclonal antibody, VRC-HIVMAB075-00-AB (VRC07-523LS), administered in multiple doses and routes to healthy, HIV-uninfected adults

DAIDS DOCUMENT ID 38458

IND 137719 HELD BY DAIDS

CLINICAL TRIAL SPONSORED BY

Division of AIDS (DAIDS)
National Institute of Allergy and Infectious Diseases (NIAID)
National Institutes of Health (NIH)
Department of Health and Human Services (DHHS)
Bethesda, Maryland, USA

STUDY PRODUCT PROVIDED BY

Dale and Betty Bumpers Vaccine Research Center (VRC), NIAID, NIH, DHHS
Bethesda, Maryland, USA

March 8, 2018
HVTN 127/HPTN 087
Version 2.0

Contents

1 Ethical considerations 5

2 IRB/EC review considerations 7
2.1 Minimized risks to participants 7
2.2 Reasonable risk/benefit balance 8
2.3 Equitable participant selection 8
2.4 Appropriate informed consent 8
2.5 Adequate safety monitoring 9
2.6 Protect privacy/confidentiality 9

3 Overview 10
3.1 Protocol Team 13

4 Background 15
4.1 Rationale for trial concept 15
4.2 VRC01 15
4.3 VRC01LS 17
4.4 VRC07-523LS 18
4.5 Trial design rationale 20
4.6 Plans for future product development and testing 22
4.7 Preclinical safety studies of VRC07-523LS 22
4.8 Preclinical PK and challenge studies of VRC07-523LS 24
4.9 Clinical studies 25
4.10 Potential risks of study products and administration 37

5 Objectives and endpoints 41
5.1 Primary objectives and endpoints 41
5.2 Secondary objectives and endpoints 41
5.3 Exploratory objectives 42

6 Statistical considerations 43
6.1 Accrual and sample size calculations 43
6.2 Randomization 48
6.3 Blinding 49
6.4 Statistical analyses 49

7 Selection and withdrawal of participants 55
7.1 Inclusion criteria 55
7.2 Exclusion criteria 57
7.3 Participant departure from study product administration schedule or withdrawal 60

8 Study product preparation and administration 64
8.1 Study product regimen 64
8.2 Study product formulation 65
8.3 Preparation of study products 65
8.4 Administration 70
8.5 Acquisition of study products 72
8.6 Pharmacy records 72
8.7 Final disposition of study products 72

9 Clinical procedures 73
- 9.1 Informed consent 73
- 9.2 Pre-enrollment procedures 75
- 9.3 Enrollment and study product administration visits 76
- 9.4 Follow-up visits 78
- 9.5 HIV counseling and testing 79
- 9.6 Contraception status 80
- 9.7 Urinalysis 80
- 9.8 Assessments of Solicited AEs 81
- 9.9 Visit windows and missed visits 82
- 9.10 Early termination visit 83
- 9.11 Pregnancy 83

10 Laboratory 84
- 10.1 CRS laboratory procedures 84
- 10.2 Total blood volume 84
- 10.3 VRC07-523LS concentrations 84
- 10.4 Endpoint assays: humoral 85
- 10.5 Exploratory studies 85
- 10.6 Specimen storage and other use of specimens 85
- 10.7 Biohazard containment 86

11 Safety monitoring and safety review 87
- 11.1 Safety monitoring and oversight 87
- 11.2 Safety reporting 88
- 11.3 Safety pause and prompt PSRT AE review 91
- 11.4 Review of cumulative safety data 93
- 11.5 Study termination 93

12 Protocol conduct 94
- 12.1 Social impacts 95
- 12.2 Emergency communication with study participants 95

13 Version history 96

14 Document references (other than literature citations) 98

15 Acronyms and abbreviations 100

16 Literature cited 103

Appendix A Sample informed consent form 110

Appendix B Approved birth control methods (for sample informed consent form) 127

Appendix C Sample consent form for use of samples and information in other studies 129

Appendix D Table of procedures (for sample informed consent form) 133

Appendix E Schema graphic (for sample informed consent form) 134

Appendix F Laboratory procedures 135

Appendix G Procedures at CRS 137

Appendix H Adverse events of special interest (AESI) 139

Appendix I Low risk guidelines for the US and Switzerland..140
Appendix J Protocol Signature Page 142

1 Ethical considerations

It is critical that universally accepted ethical guidelines are followed at all sites involved in the conduct of clinical trials. The HVTN and HPTN (hereafter, referred to as the "Networks") have addressed ethical concerns in the following ways:

- Network trials are designed and conducted to enhance the knowledge base necessary to find new methods for preventing HIV infection, using methods that are scientifically rigorous and valid, and in accordance with International Council for Harmonisation of Technical Requirements for Pharmaceuticals for Human Use (ICH) Good Clinical Practice (GCP) guidelines.

- Network scientists and operational staff incorporate the philosophies underlying major codes (1-3), declarations, and other guidance documents relevant to human subjects research into the design and conduct of HIV vaccine clinical trials.

- Network scientists and operational staff are committed to substantive community input—into the planning, conduct, and follow-up of its research—to help ensure that locally appropriate cultural and linguistic needs of study populations are met. Community Advisory Boards (CAB) are required by DAIDS and supported at all Network research sites to ensure community input.

- Network clinical trial staff counsel study participants routinely on how to reduce HIV risk. Participants who become HIV-infected during the trial are provided counseling on notifying their partners and about HIV infection according to local guidelines. Staff members will also counsel them about reducing their risk of transmitting HIV to others.

- The Networks require that all international Network sites lacking national plans for providing ART develop plans for the care and treatment of participants who acquire HIV infection during a trial. Each plan is developed in consultation with representatives of host countries, communities from which potential trial participants will be drawn, sponsors, and the Networks. Participants who become HIV-infected during the trial will be referred to programs for ART provision when the appropriate criteria for starting ART are met. If a program for antiretroviral therapy (ART) provision is not available at a site and ART is needed, a privately established fund will be used to pay for access to treatment to the fullest extent possible.

- The Networks provide training so that all participating sites similarly ensure fair participant selection, protect the privacy of research participants, and obtain meaningful informed consent. During the study, participants will have their wellbeing monitored, and to the fullest extent possible, their privacy protected. Participants may withdraw from the study at any time.

- Prior to implementation, Network trials are rigorously reviewed by scientists who are not involved in the conduct of the trials under consideration.

- Network trials are reviewed by local and national regulatory bodies and are conducted in compliance with all applicable national and local regulations.

- The Networks design their research to minimize risk and maximize benefit to both study participants and their local communities. For example, Network protocols provide enhancement of participants' knowledge of HIV and HIV prevention, as well as counseling, guidance, and assistance with any social impacts that may result from research participation. Network protocols also include careful medical review of each research participant's health conditions and reactions to study products while in the study.

- Network research aims to benefit local communities by directly addressing the health and HIV prevention needs of those communities and by strengthening the capacity of the communities through training, support, shared knowledge, and equipment. Researchers involved in Network trials are able to conduct other critical research in their local research settings.

- The Networks recognize the importance of institutional review and value the role of in country Institutional Review Boards (IRBs), Ethics Committees (ECs), and other Regulatory Entities (REs) as custodians responsible for ensuring the ethical conduct of research in each setting.

2 IRB/EC review considerations

US Food and Drug Administration (FDA) and other US federal regulations require IRBs/ECs/REs to ensure that certain requirements are satisfied on initial and continuing review of research (Title 45, Code of Federal Regulations (CFR), Part 46.111(a) 1-7; 21 CFR 56.111(a) 1-7). The following section highlights how this protocol addresses each of these research requirements. Each Network Investigator welcomes IRB/EC/RE questions or concerns regarding these research requirements.

This trial is being conducted in the US and Switzerland with funding from the US NIH. Due to this, the trial is subject to both US and local regulations and guidelines on the protection of human research subjects and ethical research conduct. Where there is a conflict in regulations or guidelines, the regulation or guideline providing the maximum protection of human research subjects will be followed.

In compliance with international and local (as appropriate) GCP guidelines, each research location has a locally based Principal Investigator (PI) who is qualified to conduct (and supervise the conduct of) the research; and the research addresses an important local health need for an HIV prevention method. In addition, the investigators take responsibility for the conduct of the study and the control of the study products, including obtaining all appropriate regulatory and ethical reviews of the research. Each participating clinical research site (CRS) has a standard operating procedure for ensuring that participants receive and understand the necessary information to make a decision whether or not to consent to the research.

The sections below address each of the review concerns by IRBs/ECs and any applicable REs regarding how the research will be conducted.

2.1 Minimized risks to participants

45 CFR 46.111 (a) 1 and 21 CFR 56.111 (a) 1: Risks to subjects are minimized.

This protocol minimizes risks to participants by (a) correctly and promptly informing participants about risks so that they can join in partnership with the researcher in recognizing and reporting harms; (b) respecting local/national blood draw limits; (c) performing direct observation of participants post-product administration and collecting information regarding side effects for several days post-product administration; (d) having staff properly trained in administering study procedures that may cause physical harm or psychological distress, such as blood draws, infusions, injections, HIV testing and counseling and HIV risk reduction counseling; (e) providing HIV risk reduction counseling and checking on contraception use (for persons assigned female at birth); and (f) providing safety monitoring.

2.2 Reasonable risk/benefit balance

45 CFR 46.111(a) 2 and 21 CFR 56.111(a) 2: Risks to subjects are reasonable in relation to anticipated benefits, if any, to subjects, and the importance of the knowledge that may reasonably be expected to result.

In all public health research, the risk-benefit ratio may be difficult to assess because the benefits to a healthy participant are not as apparent as they would be in treatment protocols, where a study participant may be ill and may have exhausted all conventional treatment options. However, this protocol is designed to minimize the risks to participants while maximizing the potential value of the knowledge it is designed to generate.

2.3 Equitable participant selection

45 CFR 46.111 (a) 3 and 21 CFR 56.111 (a) 3: Subject selection is equitable

This protocol has specific inclusion and exclusion criteria for investigators to follow in admitting participants into the protocol. Participants are selected because of these criteria and not because of positions of vulnerability or privilege. Investigators are required to maintain screening and enrollment logs to document volunteers who screened into and out of the protocol and for what reasons.

2.4 Appropriate informed consent

45 CFR 46.111 (a) 4 and 5 and 21 CFR 56.111 (a) 4 and 5: Informed consent is sought from each prospective subject or the subject's legally authorized representative as required by 45 CFR 46.116 and 21 CFR Part 50; informed consent is appropriately documented as required by 45 CFR 46.117 and 21 CFR 50.27

The protocol specifies that informed consent must be obtained before any study procedures are initiated and assessed throughout the trial (see Section 9.1). Each site is provided training in informed consent by the Networks as part of its entering the Network. The Networks require a signed consent document for documentation, in addition to chart notes or a consent checklist.

2.5 Adequate safety monitoring

45 CFR 46.111 (a) 6 and 21 CFR 56.111 (a) 6: There is adequate provision for monitoring the data collected to ensure the safety of subjects.

This protocol has extensive safety monitoring in place (see Section 11). Safety is monitored daily by the HVTN Clinical Safety Specialist (CSS) or HVTN Core designee and routinely by the HVTN 127/HPTN 087 Protocol Safety Review Team (PSRT). In addition, the HVTN Safety Monitoring Board (SMB) periodically reviews study data.

2.6 Protect privacy/confidentiality

45 CFR 46.111 (a) 7 and 21 CFR 56.111 (a) 7: There are adequate provisions to protect the privacy of subjects and maintain the confidentiality of data.

Privacy refers to an individual's right to be free from unauthorized or unreasonable intrusion into his/her private life and the right to control access to individually identifiable information about him/her. The term "privacy" concerns research participants or potential research participants as individuals whereas the term "confidentiality" is used to refer to the treatment of information about those individuals. This protocol respects the privacy of participants by informing them about who will have access to their personal information and study data (see Appendix A). The privacy of participants is protected by assigning unique identifiers in place of the participant's name on study data and specimens. In the United States, research participants in Network protocols are protected by a Certificate of Confidentiality from the US NIH, which can prevent disclosure of study participation even when that information is requested by subpoena. Participants are told of the use and limits of the certificate in the study consent form. In addition, each staff member at each study site in this protocol signs an Agreement on Confidentiality and Use of Data and Specimens with the Networks and each study site participating in the protocol is required to have a standard operating procedure on how the staff members will protect the confidentiality of study participants.

3 Overview

Title

A multicenter, randomized, partially blinded phase 1 clinical trial to evaluate the safety and serum concentrations of a human monoclonal antibody, VRC-HIVMAB075-00-AB (VRC07-523LS), administered in multiple doses and routes to healthy, HIV-uninfected adults

Primary objective(s)

Primary objective 1:

To evaluate the safety and tolerability of different doses of VRC07-523LS administered **IV, SC, and IM** by repeat dosing every 16 weeks for a total of 5 administrations to healthy adults

Primary objective 2:

To evaluate the serum concentrations of VRC07-523LS administered IV, SC, and IM over a total of 6 regimens

Study products and routes of administration

- VRC07-523LS: VRC-HIVMAB075-00-AB (VRC07-523LS) is a human monoclonal antibody (mAb) targeted to the HIV-1 CD4 binding site. It was developed by the VRC/NIAID/NIH and manufactured under current Good Manufacturing Practice regulations at the VRC Pilot Plant operated under contract by the Vaccine Clinical Materials Program, Leidos Biomedical Research, Inc., Frederick, MD. Product is provided at 100 ± 10 mg/mL as 10 mL glass vials with a 6.25 ± 0.1 mL fill volume and 3 mL glass vials with a 2.25 mL ± 0.1 mL fill volume.

- Placebo: Sodium Chloride Injection, USP 0.9%

Table 3-1 Schema

Group	N	Route	Dose	Product administration schedule				
				W0	W16	W32	W48	W64
1	20	IV	2.5 mg/kg	X	X	X	X	X
2	20	IV	5 mg/kg	X	X	X	X	X
3	20	IV	20 mg/kg	X	X	X	X	X
4	20	SC	2.5 mg/kg	X	X	X	X	X
5	20	SC	5 mg/kg	X	X	X	X	X
6	20	IM	2.5 mg/kg	X	X	X	X	X
	4		Placebo	X	X	X	X	X
**Total**	**124**							

IV = intravenous infusion
SC = subcutaneous injection
IM = intramuscular injection
All groups to enroll simultaneously.

Participants

124 healthy, HIV-1–uninfected volunteers aged 18 to 50 years

Design

Multicenter, randomized clinical trial

Duration per participant

26 months of scheduled clinic visits

Estimated total study duration

32 months (includes enrollment and follow-up)

Investigational New Drug (IND) sponsor

DAIDS, NIAID, NIH, DHHS (Bethesda, Maryland, USA)

Study product providers

- VRC07-523LS: Dale and Betty Bumpers Vaccine Research Center (VRC), NIAID, NIH, DHHS (Bethesda, Maryland, USA)

Core operations

HVTN Vaccine Leadership Group/Core Operations Center, Fred Hutchinson Cancer Research Center (Fred Hutch) (Seattle, Washington, USA)

HPTN Leadership Operations Center, FHI360 (Durham, North Carolina, USA)

Statistical and data management center (SDMC)

Statistical Center for HIV/AIDS Research and Prevention (SCHARP), Fred Hutch (Seattle, Washington, USA)

HIV diagnostic laboratory

University of Washington Virology Specialty Laboratory (UW-VSL) (Seattle, Washington, USA)

Endpoint assay laboratories

- Duke University Medical Center (Durham, North Carolina, USA)
- Fred Hutch/University of Washington (Seattle, Washington, USA)
- Vaccine Research Center – Immunology Testing Laboratory (Gaithersburg, MD)

Study sites

HVTN and HPTN CRSs to be specified in the Site Announcement Memo

Safety monitoring

HVTN 127/HPTN 087 PSRT; HVTN Safety Monitoring Board (SMB)

3.1 Protocol Team

Protocol leadership

HVTN Chair	Stephen Walsh<br>Brigham & Women's Hospital<br>617-525-8418<br>swalsh@bwh.harvard.edu	*Statisticians*	Ollivier Hyrien<br>SCHARP, Fred Hutch<br>206-667-5780<br>ohyrien@fhcrc.org
HPTN Chair	Cynthia Gay<br>University of North Carolina, Chapel Hill<br>919-843-2726<br>cynthia_gay@med.unc.edu		Timothy Skalland<br>SCHARP, Fred Hutch<br>206-667-1186<br>tskallan@fredhutch.org
Protocol Team leader	Shelly Karuna<br>HVTN Core, Fred Hutch<br>206-667-4355<br>skaruna@fredhutch.org	*Laboratory leads*	David Montefiori<br>HVTN Laboratory Program, Duke Univ.<br>919-684-5278<br>monte@duke.edu
Medical officers	Jane Baumblatt<br>DAIDS, NIAID<br>301-761-7754<br>jane.baumblatt@nih.gov		Estelle Piwowar-Manning<br>HPTN Laboratory Program, JHU<br>410-614-6736<br>epiwowa@jhmi.edu
	Wairimu Chege<br>DAIDS, NIAID<br>240-292-4786<br>wairimu.chege@nih.gov		

Other contributors to the original protocol

Core medical monitor	Shelly Karuna<br>HVTN Core, Fred Hutch	*DAIDS protocol pharmacist*	Oladapo Alli<br>DAIDS, NIAID<br>240-627-3593
HPTN Pharmacologist	Julie Dumond<br>University of North Carolina, Chapel Hill	*Statistician*	Yunda Huang<br>SCHARP, Fred Hutch
Product developer representatives	Julie Ledgerwood<br>VRC	*Statistical research associate*	Xue Han<br>SCHARP, Fred Hutch
	Barney Graham<br>VRC	*Clinical safety specialist*	Lauryn Takisaki<br>HVTN Core, Fred Hutch
	Martin Gaudinski<br>VRC	*Clinical research manager*	Phil Andrew<br>HPTN LOC, FHI360

	Lucio Gama<br>VRC	*Clinical trials manager*	Carissa Karg<br>HVTN Core, Fred Hutch
HVTN Laboratory program reps	On Ho<br>HVTN Laboratory Program, Fred Hutch	*Clinical data manager*	Ingrid Durenberger<br>SCHARP, Fred Hutch
	John Hural<br>HVTN Laboratory Program, Fred Hutch	*Senior clinical data manager*	Gina Escamilla<br>SCHARP, Fred Hutch
HPTN Laboratory program rep	Vanessa Cummings<br>HPTN Laboratory Program, JHU	*SDMC Associate director of lab science*	April Randhawa<br>SCHARP, Fred Hutch
HVTN regulatory affairs associate	Meg Brandon<br>HVTN Core, Fred Hutch	*Protocol development manager*	Carter Bentley<br>HVTN Core, Fred Hutch
Clinic coordinators	Rotrease Regan<br>The Ponce de Leon Center CRS	*HVTN Community engagement unit representative*	Gail Broder<br>HVTN Core, Fred Hutch
	Elvin Fontana-Martinez<br>Boston-Brigham CRS	*HPTN Community program associate*	Jontraye Davis<br>HPTN LOC, FHI360
Community Advisory Board (CAB) members	Jamel Young<br>The Ponce de Leon Center CAB	*Technical editor*	Richa Chaturvedi<br>HVTN Core, Fred Hutch
	Gary Daffin<br>Fenway and Brigham & Women’s CAB		
Community educator/ recruiters	Jorge Benitez<br>Columbia Physicians and Surgeons		
	Noshima Darden-Tabb<br>University of North Carolina, Chapel Hill		

4 Background

4.1 Rationale for trial concept

Effective biomedical interventions are urgently needed to reduce the acquisition of HIV. The global HIV-1 epidemic continues and while many countries have made progress toward reducing HIV incidence, micro-epidemics of infection continue to occur in nearly all regions, even in countries possessing the full toolkit of proven prevention approaches (4-7). UNAIDS estimates that there were 2.1 million new HIV infections worldwide in 2015 (8). Multiple advances in prevention, including treatment as prevention (TasP) (9) and pre- or postexposure prophylaxis (PrEP or PEP) (10, 11), have been made in recent years. However, these are hampered by side effects, cost, access, and significant adherence challenges, and thus remain imperfect tools to end the ongoing HIV epidemic. A biomedical HIV prevention approach that exhibits sustained activity over an extended time period, that has a safety profile acceptable for healthy persons, and whose effectiveness is less dependent upon consistent adherence, is still needed. Ultimately, an effective vaccine will be necessary to better control the HIV pandemic (12, 13).

An alternative approach to prevention and treatment of infectious diseases is passive administration of antibodies, a strategy that has been employed for more than 100 years against diverse disease targets (14, 15). Hyperimmune serum is still used for hepatitis A and B prophylaxis (16, 17) and for PEP for rabies (18), measles (19), varicella zoster (20), and other infectious diseases (15). Furthermore, palivizumab, a monoclonal antibody directed against the F protein of respiratory syncytial virus (RSV), has been used for more than 15 years to prevent RSV infection in high-risk infants (21).

Over the past several years, there has been a concerted and notably successful effort to isolate broadly neutralizing antibodies (bNAbs) to HIV-1 from chronically infected individuals (22-38). Subsequent studies have provided considerable insight into the sites these antibodies target on HIV-1 and the mechanisms by which they neutralize the virus (25, 26, 35, 39). This research has informed efforts to design recombinant protein immunogens that can elicit such antibodies (40-43), prompting optimism that vaccines that elicit bNAbs against HIV-1 can be developed (41, 44). In addition, the availability of bNAbs against HIV opens the exciting possibility of antibody mediated prevention (AMP) of HIV infection (45).

4.2 VRC01

The Vaccine Research Center (VRC) at the National Institute of Allergy and Infectious Diseases (NIAID) at the National Institutes of Health (NIH) developed VRC01, a potent and broadly neutralizing HIV-1 human mAb targeted against the HIV-1 CD4 binding site. VRC01 is currently in clinical trials under IND 113,611

[prevention indication] and IND 126,001 and IND 126,664 [therapeutic indication]. VRC01 was originally discovered in an individual infected with HIV-1 for more than 15 years and whose immune system controlled the virus without ART (24, 46).

In in vitro studies, 90% of 190 HIV-1 pseudoviruses across all clades tested demonstrated sensitivity to neutralization by VRC01. VRC01 has a 50% inhibitory concentration (IC_{50}) of < 50 mcg/mL against 91% of primary HIV-1 isolates and IC_{50} < 1 mcg/mL against 72% of HIV-1 isolates.

Clinical studies of VRC01 started in September 2013. VRC01 has been tested in HIV-infected adults under VRC 601 (47), in healthy adults under VRC 602 (48) and HVTN 104 (49), and has been found to be safe and well tolerated at 5-40 mg/kg administered intravenously (IV) and at 5 mg/kg subcutaneously (SC) in both HIV-infected (47) and HIV-uninfected adult populations (48). A study in infants born from HIV-1 infected mothers is ongoing under IMPAACT P1112 [NCT02256631].

The efficacy of VRC01 in the prevention of HIV-1 infection is currently being evaluated in 2 phase 2b studies. HVTN 704/HPTN 085, a phase 2b clinical trial of VRC01 administered every 8 weeks for 72 weeks (10 infusions) IV to HIV-uninfected men and transgender persons who have sex with men, started in April 2016 [NCT02716675] while HVTN 703/HPTN 081, a phase 2b clinical trial of VRC01 administered every 8 weeks for 72 weeks (10 infusions) IV to HIV-uninfected women in sub-Saharan Africa, began in May 2016 [NCT02568215].

Studies evaluating potential treatment indications for VRC01 were initiated in 2015 in HIV-1 infected adults. The safety and virological effect of VRC01 administration during a brief analytical treatment interruption (ATI) was assessed in ACTG A5340 (50). A study evaluating the effect of VRC01 on the HIV-1 proviral reservoir (ACTG A5342; NCT02411539) was recently completed. Two additional studies evaluating the safety and therapeutic efficacy of VRC01 during acute HIV infection (RV398; NCT02591420) and during an ATI in patients who initiated ART during acute HIV infection (RV397; NCT02664415) were launched in 2016.

Cumulatively, as of July 2017, across all phase 1 trials and in the two phase 2b AMP studies, VRC01 has been administered by either IV or SC routes to more than 2300 HIV-uninfected and HIV-infected adults and 33 HIV-uninfected infants.

The majority of unsolicited adverse events (AEs) in all studies have been no greater than Grade 2 in severity. Overall, VRC01 administration in the dose range from 1 to 40 mg/kg IV and at 5 and 40 mg/kg SC has been assessed as well-tolerated and safe for further evaluation.

However, the relatively short half-life of VRC01 limits the feasibility of wider implementation of this mAb to treat HIV infection, or as part of a global public health strategy to prevent HIV-1 acquisition.

4.3 VRC01LS

Recently, the VRC developed a VRC01 variant, designated VRC01LS, which was designed to have a longer half-life by exploiting the functions of the neonatal Fc receptor (FcRn). The FcRn regulates the half-life of IgG antibodies through pH-dependent binding to IgG Fc regions, which favors binding at low pH (eg, lysosomal compartments and vaginal lumen) and release at neutral pH (eg, peripheral blood) (51, 52). The FcRn is expressed in endothelial cells of the vasculature and some myeloid cells, where it can rescue IgG from lysosomal degradation by binding these molecules within this low pH compartment, then releasing them back into circulation (53, 54). In the intestinal epithelium, the FcRn transports IgG bi-directionally; it transports IgG into the intestinal lumen and it recycles complexed antibody back to the tissue to support the induction of immunity, tolerance and/or susceptibility (55-58). In the genital tract, FcRn is expressed on columnar epithelial cells lining the human penile urethra and the endocervix, as well as on reserve epithelial cells lining the vagina. Thus, the tissue distribution of FcRn enables the delivery of IgG to relevant mucosal compartments and may thereby contribute to protection or susceptibility; the latter possibility has been suggested in the case of non-neutralizing Abs, which may mediate the transmission of bound, infectious particles via FcRn transport pathways (55, 59, 60).

The LS mutation (M428L/N434S) to the constant region of the VRC01 antibody enhances its binding affinity for FcRn at low pH, yet does not affect its release at pH 7.4 (61). Thus, the LS modification was found to alter antibody turnover in nonhuman primate (NHP) models, with increased persistence in serum and slower decay in rectal and cervicovaginal tissue. Intravenous administration of VRC01LS also afforded increased protection against intra-rectal challenge with simian-human immunodeficiency virus (SHIV) (61). Consequently, the increased persistence of anti-HIV neutralizing antibodies in mucosal tissue could have significant advantages in preventing HIV acquisition, as increased maintenance at the portals of entry might allow for consistent protective levels with less frequent administrations and/or reduced dosing.

VRC 606 was the first study of the VRC-HIVMAB080-00-AB (VRC01LS) monoclonal antibody (mAb) in healthy adults. VRC01LS was deemed safe and well tolerated at doses of 5-40 mg/kg IV and at 5 mg/kg SC (see Section 4.9.2.1). VRC01LS is also being studied in the ongoing HVTN 116 clinical trial [NCT02797171].

4.4 VRC07-523LS

The VRC has also developed VRC07-523LS, a highly potent and broadly neutralizing HIV-1 human mAb targeted against the HIV-1 CD4 binding site. Numerous HIV-1 neutralizing mAbs from the VRC01 family, including VRC07 ("07" denotes sequential numbering when discovered), have now been isolated from the same individual (donor 45) who, as noted above, was infected with HIV-1 for more than 15 years and whose immune system controlled the virus without ART (62). Through advances in B-cell immunology and structure-guided optimization techniques, VRC07-523 ("523" denotes sequential numbering when engineered variant generated), and later VRC07-523LS ("LS" denotes 2 specific amino acid mutations), were developed with potency and breadth greater than those of early antibodies (62).

Briefly, the VRC07 (wild-type) heavy chain was identified by deep sequencing of heavy chain transcripts from peripheral blood B cells isolated from donor 45 (24) based on its similarity to the VRC01 mAb and paired with the VRC01 (wild-type) light chain (62). To increase neutralization potency and breadth, a series of amino acid mutations were introduced; mutations that resulted in autoreactivity were rejected (62). The mutations that together define the 523 designation are a glycine to histidine mutation at residue 54 of the heavy chain, a deletion of the first two amino acids, glutamate and isoleucine, from the light chain, and a valine to serine mutation at the third amino acid residue of the light chain (62). The LS designation specifies methionine to leucine (L) and asparagine to serine (S) (M428L/N434S, referred to as LS) changes within the C-terminus of the heavy chain constant region. The LS mutation was introduced by site-directed mutagenesis to increase the binding affinity for the neonatal Fc-receptor (FcRn), resulting in increased recirculation of functional IgG (61, 63) and thereby increasing plasma half-life.

VRC07-523LS was found to be 5-to 8-fold more potent than VRC01 (see Figure 4-1), with an inhibitory concentration $IC_{50} < 50$ mcg/mL against 96% of HIV-1 pseudoviruses representing the major circulating HIV-1 clades, and an $IC_{50} < 1$ mcg/mL against 92% of HIV-1 viruses tested (62). Compared with other VRC07 derivatives, VRC07-523LS displayed minimal levels of autoreactivity.

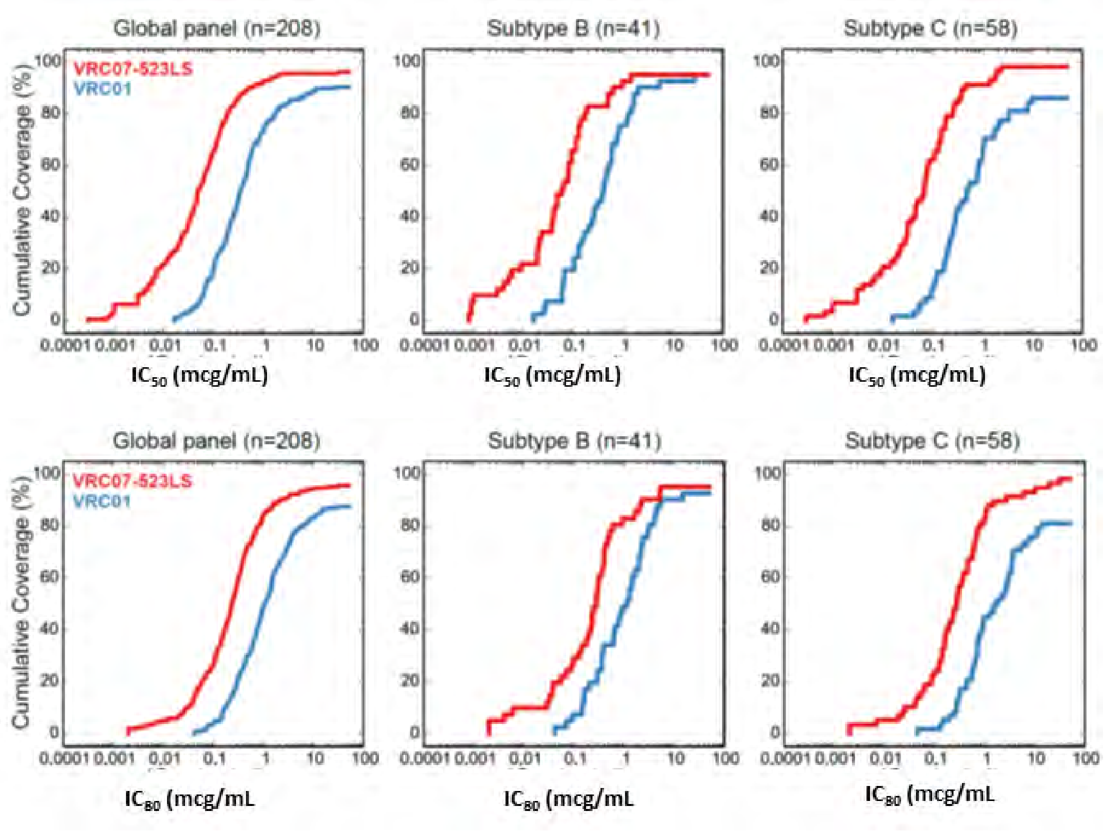

Figure 4-1 VRC07-523LS neutralizes a higher fraction of HIV-1 isolates at a lower concentration than VRC01 (data courtesy of Kshitij Wagh and Bette Korber)

In vivo proof-of-concept studies in rhesus macaques showed that VRC07-523LS is about 5-fold more potent than VRC01LS and has a 2-fold longer half-life (9.8 days) than VRC07 (4.9 days) after a single dose of mAb at 10 mg/kg administered IV (62). The increased neutralization potency and breadth in vitro and prolonged half-life of VRC07-523LS correlated with improved protection against SHIV challenge in vivo (Figure 4-2; see also Section 4.8). Taken together, these data suggest that VRC07-523LS has considerable potential for prevention of HIV-1 infection in humans.

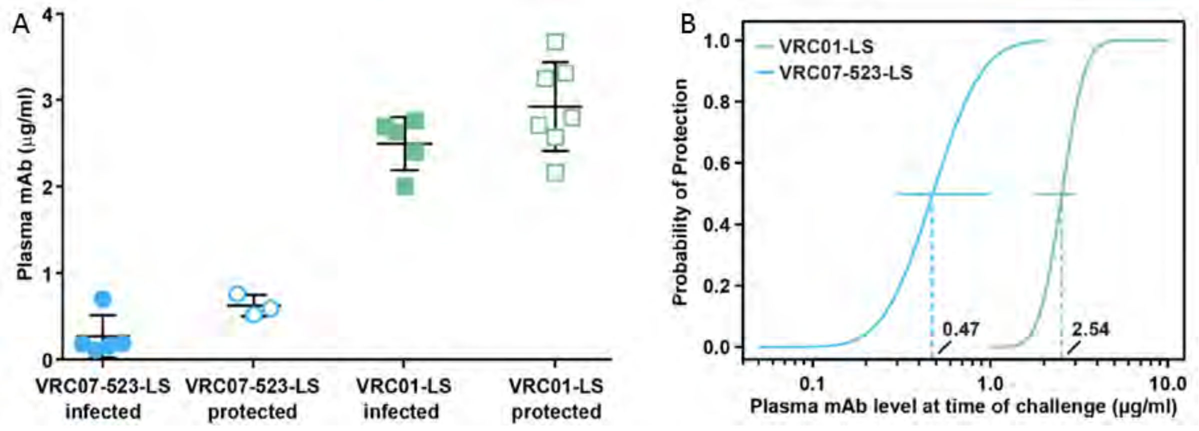

Figure 4-2 The increased neutralization potency of VRC07-523LS correlates with protection at lower antibody levels in a SHIV-rhesus macaque model compared with VRC01LS. (A) Rhesus macaques were administered 0.2 mg/kg (n = 4) or 0.05 mg/kg (n = 4) of VRC07-523LS or 0.3 mg/kg of VRC01LS (n = 12), challenged with SHIV-BaLP4 intrarectally on day 5, and plasma concentrations of VRC07-523LS and VRC01LS were assayed by ELISA. (B) Regression model based on plasma mAb concentration at the time of infection. From (62).

4.5 Trial design rationale

4.5.1 Dose and schedule

With the exception of IM administration, the VRC07-523LS doses and intervals between doses in this trial are based on human studies of VRC01 (Section 4.9.1), preclinical and clinical studies of VRC01LS (Sections 4.3 and 4.9.2), and preclinical and clinical studies of VRC07-523LS (Sections 4.4, 4.8, and 4.9.3). As pre-clinical dosing via the IM route has been conducted at the 2.5 mg/kg dose, this is the dose administered IM in this protocol. For comparison to other routes, the IM dosing schedule defaults to that of the IV and SC administration (see Section 6.1.2).

PK data obtained in this study that assesses a range of doses and routes of administration, together with the data obtained in VRC 605 and in other trials of VRC07-523LS, will allow population PK analysis and predictive dose-regimen selection modeling.

In addition, the study design allows comparisons between different dose/route groups, including the following:

- 2.5 mg/kg IV vs. 5mg/kg IV vs. 20 mg/kg IV (Groups 1, 2, and 3);
- 2.5 mg/kg SC vs 5 mg/kg SC (Groups 4 and 5);
- 5 mg/kg IV vs 5 mg/kg SC (Groups 2 and 5); and
- 2.5 mg/kg SC vs IV vs IM (Groups 1, 4, and 6).

4.5.2 Rationale for the exclusion of placebo recipients in IV and SC treatment groups

Substantial safety data have already been collected over several trials on closely related products, including VRC01 and VRC01LS. These studies have demonstrated the safety and tolerability of the IV and SC administration of these products at doses similar to or greater than those proposed in this study. For example, in VRC 606, the doses for VRC01LS in HIV-uninfected participants ranged between 1 and 40 mg/kg for IV administrations, and 5 mg/kg for SC injections. Moreover, in multiple studies, including VRC 601, VRC 602, HVTN 104, RV397, RV398, A5340, and A5342, doses for VRC01 in HIV-uninfected and/or HIV-infected participants ranged between 1 and 40 mg/kg for IV administrations, and between 5 and 40 mg/kg for SC injections.

One of the largest of the phase 1 trials, HVTN 104, evaluated VRC01 in 88 participants. Twenty-eight percent of IV infusions and 14% of SC injections were associated with mild pain or tenderness, with 55% of VRC01 recipients and 50% of placebo recipients experiencing mild pain and/or tenderness at the infusion or injection site at some point during the trial. Over 75% of IV infusions and SC injections in HVTN 104 were associated with no systemic reactogenicity; 56% of VRC01 recipients and 75% of placebo recipients experienced systemic reactogenicity at some point during the trial. Reactogenicity, when present, was typically mild or moderate (Mayer et al, PLoS Medicine, manuscript submitted).

As well, mAbs against other pathogens have been licensed at similar or higher dose levels. For example, the prevention of severe RSV in infants is generally treated via monthly IM injection of a dose of 15 mg/kg throughout the RSV season (21). Other investigational mAbs directed at pathogens that have been safely taken into efficacy trials include a *Clostridium difficile* anti-toxin mAb administered at a 10 mg/kg dosage (64) and a mAb directed at hepatitis C virus administered at a 50 mg/kg dosage (65), much higher than the dose levels proposed in this study.

Furthermore, the inclusion of placebo recipients would provide limited power to compare the rates of safety and tolerability events between active and placebo arms unless they were included in large numbers in each of the 5 IV and SC groups (66). For instance, with 20 participants in the active treatment arm of any of these groups, the study would need 20 placebo recipients per group to achieve ~80% power to detect a difference between the rates of AE/SAE in the active vs. placebo arms if the true AE/SAE rates in the placebo and in the active arm were 4% and 34%, respectively. These calculations assume a one-sided test comparing two independent binomial proportions with a significant level (type-1 error) of 5%.

In addition, drug serum concentration levels will only be present in VRC07-523LS recipients and participants' baseline samples can serve as controls for serum level assessments. Thus, the inclusion of placebo groups is not necessary for the PK objectives of this trial. Data from multiple groups will be evaluated

concurrently by laboratory staff who will remain blinded to group assignment, which would help to avoid group-specific bias in the assessment of various drug activity endpoints.

IM administration of an anti-HIV mAb will be evaluated for the first time in humans in this study. Since reactions to IM administration may not be comparable to those of IV or SC administration, a small number of placebo recipients have been included in the IM group to blind post-administration safety and tolerability assessments.

4.6 Plans for future product development and testing

This study is the next step in the characterization of VRC07-523LS and is designed to identify an optimal dose and route of administration of the antibody as well as to determine if anti-drug antibodies (ADA) or side effects emerge with more prolonged administration of the antibody.

This study will further characterize and compare the pharmacokinetics of IV and SC routes of administration at multiple doses. In addition, this study will provide the first characterization of the safety and pharmacokinetics of an anti-HIV mAb administered IM, which may ultimately prove to be a more feasible and acceptable route of administration than IV or SC.

The breadth of information obtained is expected to inform further study of VRC07-523LS, including its potential for combination with other mAbs undergoing early study at doses, intervals, and routes that could be combined with one or more of the regimens evaluated here.

Furthermore, since the site and mode of activity of VRC07-523LS are similar to those of VRC01, it should be possible to infer the potential HIV preventive efficacy of different regimens of VRC07-523LS from the PK and neutralizing values found in this study, as well as from the efficacy levels determined in the ongoing phase 2b efficacy studies of VRC01. This information will contribute greatly to the design of future bNAb efficacy trials.

4.7 Preclinical safety studies of VRC07-523LS

4.7.1 In vitro safety studies

Several in vitro preclinical safety studies were performed with VRC07-523LS to assess potential off-target binding. To measure potential anti-phospholipid cross-reactivity, binding of VRC07-523LS to cardiolipin was assessed using an enzyme-linked immunosorbent assay (ELISA) and demonstrated minimal binding compared with 4E10, an earlier-generation HIV-1 specific mAb which binds strongly to cardiolipin. VRC07-523LS was also tested for cross-reactivity against a panel of various nuclear antigens using a licensed systemic lupus erythematosus

diagnostic test kit (Luminex AtheNA Multi-Lyte® ANA-II test) and did cross-react with a small subset of nuclear antigens consistent with some reactivity with nuclear antigens. In addition, VRC07-523LS was assessed for anti-phospholipid properties in a clinical activated partial thromboplastin time (aPTT) assay and compared to the anti-HIV mAbs 4E10 and VRC01 as well as palivizumab; only 4E10 showed evidence of antiphospholipid activity. By immunohistochemistry, VRC07-523LS displayed minimal binding to HEp-2 cells at 50 mcg/mL and no binding at 25 mcg/mL (62).

A tissue cross-reactivity (TCR) study was performed in accordance with "Good Laboratory Practice (GLP) for Nonclinical Laboratory Studies" to determine the potential cross-reactivity of VRC07-523LS with cryosections of human and Sprague-Dawley rat tissues. VRC07-523LS staining was similar between the human and rat tissues examined, although staining tended to be somewhat more intense and frequent in the human tissue compared to the Sprague-Dawley rat tissues. According to ICH S6(R1), monoclonal antibody binding to cytoplasmic sites generally is considered of little to no toxicologic significance. See the Investigator's Brochure for further details.

4.7.2 In vivo toxicology studies

4.7.2.1 Repeat dose IV and SC toxicity study in Sprague-Dawley rats

A repeat dose IV and SC toxicity study (IITRI Project NO. 2517-001-002) with VRC07-523LS was performed in male and female Sprague-Dawley rats in accordance with Good Laboratory Practice (GLP) for Nonclinical Laboratory Studies. Treatment with VRC07-523LS at doses up to 400 mg/kg/dose IV or 40 mg/kg/dose SC with three doses at 10 day intervals was generally well tolerated as most findings were reversible and no longer seen at the end of the recovery period. Additionally, histologic changes were not observed in the GLP repeat dose toxicology study in the cell types with staining observed in the GLP tissue cross reactivity (TCR) study. The no observed adverse effect levels (NOAELs) for this study were 400 mg/kg IV and 40 mg/kg SC. See the Investigator's Brochure for further details.

4.7.2.2 Local tolerance of VRC07-523LS administered IM to Sprague-Dawley rats

A GLP local tolerance study (IITRI Project N0. 2749-001) was performed to evaluate tissue reactions of VRC-HIVMAB075-00-AB (VRC07-523 LS). Sprague-Dawley rats (five rats/sex in each group) were administered a single intramuscular (IM) injection of either the control (Final Formulation Buffer) or the MAb at a dose of 2.5 mg/kg on Day 1. Rats were then subjected to necropsy on Day 7 (6 days after dosing). Experimental endpoints included moribundity/mortality, daily clinical signs of toxicity, and injection site (Draize) reactogenicity scoring; body weights; plasma fibrinogen levels, serum alpha-2- macroglobulin (A2M) levels; gross pathology at necropsy; and microscopic pathology of the skeletal muscle injection sites.

No treatment-related findings were noted locally at the injection site through injection site reactogenicity observations, limb evaluations, and gross and microscopic pathology. No evidence of systemic toxicity was also noted following a single IM injection of the HIV MAb as there were no adverse clinical findings seen and no alterations in body weights and serum A2M levels. There were statistically significant changes in plasma fibrinogen levels in the MAb-treated group (compared to the controls); however, these findings were considered to be of minimal toxicological significance.

4.8 Preclinical PK and challenge studies of VRC07-523LS

When administered IV at a single dose of 10 mg/kg to male rhesus macaques (n = 4), the half-life of VRC07-523LS was 9.8 days, a 2-fold increase compared to VRC07 (4.9 days) (62). Plasma concentrations of VRC07-523LS exceeded 10 mcg/mL at day 14 and remained greater than 2 mcg/mL at day 28 (62). Detectable concentrations (> 10 ng/mL) of VRC07-523LS were measured in rectal secretions for at least 14 days (last collection point) in the 2 animals tested (62).

In a different study, VRC07-523LS was administered SC at a single dose of 10 mg/kg to male (n = 2) and female (n = 4) rhesus macaques. The half-life was about 14 days and it persisted at least 49 days (last collection point) in rectal, vaginal, and nasal secretions. See the Investigator's Brochure for further details.

When administered IV at a single dose of 10 mg/kg to male and female cynomolgus macaques (n = 4), the half-life of VRC07-523LS was about 12 days (Figure 4-3B). In all 4 animals, plasma concentrations of VRC07-523LS exceeded 10 mcg/mL at day 14 and were greater than 3 mcg/mL at day 28, and VRC07-523LS was detectable at 28 days (last collection point) in rectal and vaginal secretions and tissues. See the Investigator's Brochure for further details.

In a SHIV challenge study, VRC07-523LS was administered to male (n = 3) and female (n = 3) rhesus macaques at a dose of 20 mg/kg IV and the plasma concentrations monitored by ELISA (Figure 4-3A). 6/6 animals were protected from SHIV-SF162P3 challenge on Day 5 after receiving a single dose of VRC07-523LS at 20 mg/kg IV. The average plasma concentration of VRC07-523LS on the day of the challenge was 114.2 mcg/mL. See the Investigator's Brochure for further details.

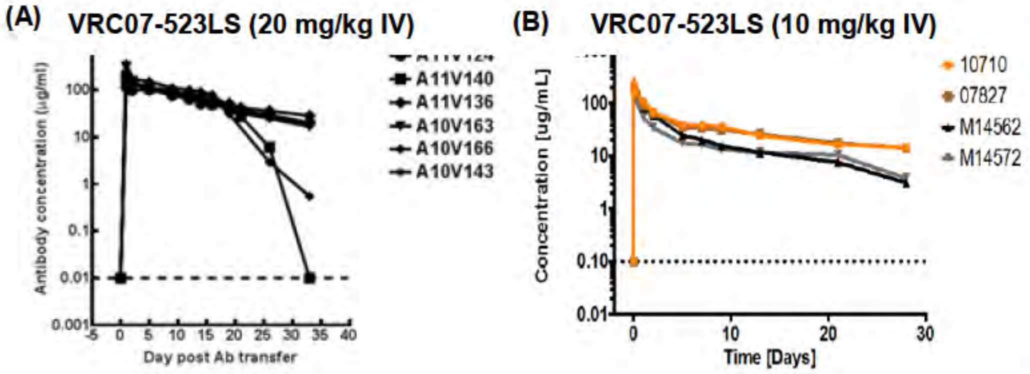

Figure 4-3 (A) VRC07-523LS was administered to male (n = 3) and female (n = 3) rhesus macaques at a dose of 20 mg/kg IV in a SHIV challenge study; all were protected against infection. (B) VRC07-523LS PK was measured in male (n = 2) and female (n = 2) cynomolgus macaques following a single administration of 10 mg/kg IV. Plasma concentrations were monitored by ELISA in both studies.

An additional challenge study assessed whether the increased neutralization potency observed with VRC07-523LS in vitro would confer greater protection in vivo compared to VRC01LS. 7/12 male rhesus macaques were protected from SHIV-BaLP4 intrarectal challenge after receiving VRC01LS at 0.3 mg/kg IV, whereas 3/4 male rhesus macaques were protected after receiving VRC07-523LS at 0.2 mg/kg IV. VRC07-523LS showed a > 5-fold increase in potency compared to VRC01LS, consistent with its ability to better neutralize viruses in vitro (62).

4.9 Clinical studies

4.9.1 Clinical studies of VRC01

4.9.1.1 VRC 601

VRC 601 (NCT01950325) was the first study of the VRC01 mAb in HIV-infected participants (47). It was a dose-escalation study to examine safety, tolerability, dose, PK, and anti- antibody immune responses. VRC 601 opened in September 2013 as a single site study at the NIH Clinical Center, Bethesda, Maryland and in total, 23 HIV-infected participants, including 15 aviremic ARV-treated participants and 8 viremic non-ARV treated participants, were infused with one or two doses of VRC01 at doses up to 40 mg/kg IV.

All IV infusions and SC injections were well tolerated with no serious adverse events (SAEs) or dose limiting toxicity. VRC 601 demonstrated evidence of VRC01-mediated antiviral effect. Analysis of the VRC 601 viral load data obtained from 8 viremic adults shows that VRC01 has a statistically significant in vivo virological effect on HIV viral load when administered as a single 40 mg/kg IV dose (Figure 4-4) (47). None of these adults were taking ART when enrolled into the study and none had started ART during the time period when the viral load data were collected. Six of the eight adult participants had ≥1 $\log_{10}$

copies/mL decrease in viral load and two participants had a viral load drop of 0.26 and 0.18 log_{10} copies/mL respectively. These interim data indicate the following for a single dose of VRC01 at 40 mg/kg IV: a statistically significant decline in viral load postinfusion at days 5 to 16; a median time of 5 days to reach ≥ 0.5-log10 decrease in viral load; and a median time of 7 days to greatest decrease in viral load postinfusion.

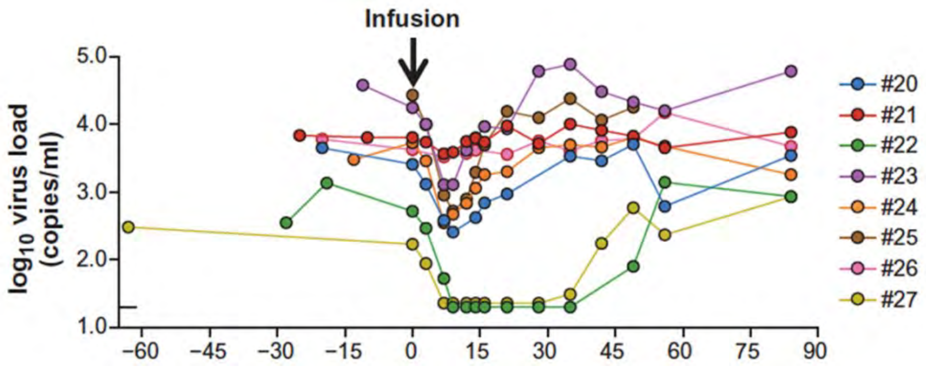

Figure 4-4 The effect of VRC01 on plasma viral load after infusion. Plasma virus load over time for eight viremic participants before and after infusion with VRC01 (day 0, indicated by arrow). Limit of detection of the assay is indicated with a tick at 20 copies/mL (47).

In VRC 601, participants were administered a single dose of VRC01 at 40 mg/kg and, therefore, a sustained effect on viral load was not expected. These data demonstrate a VRC01 mediated anti-viral effect and led to the hypothesis that repetitive dosing could have a beneficial clinical effect. This hypothesis was tested in two similar clinical trials, ACTG A5340 and NIH 15-I-0140, in which aviremic patients on stable ART underwent an ATI after receiving VRC01 at a dose of 40 mg/kg (50). No product-related safety concerns were identified in the 24 participants enrolled. Viral rebound occurred despite VRC01 serum concentrations well above 50 mcg/mL with a mean time to rebound of 4 to 6 weeks (50). While the mean time to rebound was statistically significantly different from historical controls in previous ACTG ATI studies at 4 weeks, there was no difference at 8 weeks. VRC01 was also found to exert selection pressure on emergent viruses (Figure 4-5).

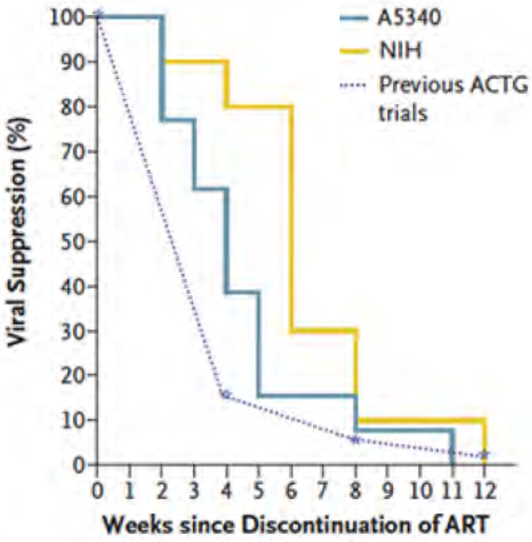

Figure 4-5 Viral suppression mediated by VRC01. Kaplan-Meier curve of plasma viremia suppression (< 200 copies/mL) after VRC01 administration in A5340 and NIH trial participants as compared with historical ACTG trials who underwent interruption of therapy without other immunotherapeutic interventions (50).

4.9.1.2 VRC 602

VRC 602 was a dose-escalation study of the VRC01 mAb in HIV-uninfected adults to examine safety, tolerability, dose, and PK of VRC01 (48). There were 3 open-label, dose escalation groups for IV administration and 1 double-blinded, placebo-controlled group for SC administration as shown in Table 4-1.

Table 4-1 VRC 602 study schema

VRC 602 Groups			VRC01 Administration Schedule	
**Group**	**Initial Enrollments**	**Additional Enrollments**	**Day 0**	**Week 4**
1	3	2	5 mg/kg IV	5 mg/kg IV
2	3	2	20 mg/kg IV	20 mg/kg IV
3	3	2	40 mg/kg IV	40 mg/kg IV
4	SC administration			
4A	3	2	5 mg/kg SC	5 mg/kg SC
4B	3	2	Placebo SC	Placebo SC
Total		25	20 participants treated with VRC01 at different dosage levels and 5 participants treated with placebo administered SC	
IV doses administered in 100 mL of normal saline over 1 hr.<br>First SC dose administered at about 15 mL/hr via SC infusion pump; participant option for second dose administration (Week 4) by direct SC injection with needle and syringe.				

All IV infusions and SC injections were well tolerated with no SAEs or dose limiting toxicity (48). PK analysis from VRC 602 revealed a VRC01 terminal half-life of 15 days across all IV infused dose groups. After the first infusion, 28-

day trough levels were 35 mcg/mL and 57 mcg/mL for the 20 mg/kg and 40 mg/kg dose groups, respectively. Following the second infusion, the 28-day trough values rose to 57 mcg/mL and 89 mcg/mL for the 20 mg/kg and 40 mg/kg dose groups, respectively (48). In addition, postinfusion VRC01 retained the expected neutralizing activity in serum and no anti-VRC01 antibodies were detected (48).

4.9.1.3 HVTN 104

HVTN 104 was a phase 1 clinical trial designed to evaluate the safety and drug levels of VRC01 administered in multiple IV or SC doses and different dosing schedules to 88 healthy, HIV-uninfected adults at 6 HVTN CRSs in 4 US cities: Boston, New York, Philadelphia, and Cleveland. The first participant enrolled in HVTN 104 on September 9, 2014 and study product administration for all participants was complete as of November 30, 2015. The study had 5 arms (Table 4-2): Group 1 evaluated the IV administration of a 40 mg/kg loading dose, with 5 subsequent 20 mg/kg doses given at 4 week intervals. Groups 2, 4, and 5 evaluated 3 infusions of 40 mg/kg, 10 mg/kg, or 30 mg/kg, respectively, given 8 weeks apart. Group 3 evaluated 5 mg/kg given every 2 weeks subcutaneously for 22 weeks after an initial IV administration at 40 mg/kg in order to inform the design of perinatal prophylaxis studies.

Table 4-2 HVTN 104 study schema

**Dose Groups**		**Study product administration schedule in months (days)**											
**Group**	**N**	**0**	**0.5 (14)**	**1 (28)**	**1.5 (42)**	**2 (56)**	**2.5 (70)**	**3 (84)**	**3.5 (98)**	**4 (112)**	**4.5 (126)**	**5 (140)**	**5.5 (154)**
1	20	VRC01 40mg/kg IV		VRC01 20mg/kg IV		VRC01 20mg/kg IV		VRC01 20mg/kg IV		VRC01 20mg/kg IV		VRC01 20mg/kg IV	
2	20	VRC01 40mg/kg IV				VRC01 40mg/kg IV				VRC01 40mg/kg IV			
3	20	VRC01 40mg/kg IV	VRC01 5mg/kg SC	VRC01 5mg/kg SC	VRC01 5mg/kg SC	VRC01 5mg/kg SC	VRC01 5mg/kg SC	VRC01 5mg/kg SC	VRC01 5mg/kg SC	VRC01 5mg/kg SC	VRC01 5mg/kg SC	VRC01 5mg/kg SC	VRC01 5mg/kg SC
	4	IV placebo for VRC01	SC placebo for VRC01	SC placebo for VRC01	SC placebo for VRC01	SC placebo for VRC01	SC placebo for VRC01	SC placebo for VRC01	SC placebo for VRC01	SC placebo for VRC01	SC placebo for VRC01	SC placebo for VRC01	SC placebo for VRC01
4	12	VRC01 10mg/kg IV				VRC01 10mg/kg IV				VRC01 10mg/kg IV			
5	12	VRC01 30mg/kg IV				VRC01 30mg/kg IV				VRC01 30mg/kg IV			
Total	88	Intravenous (IV) doses administered in 100 mL of normal saline over 1 hr<br>Subcutaneous (SC) doses administered by needle and syringe injection											

Secondary aims of HVTN 104 were to: (1) evaluate the kinetics of in vitro neutralization in serum of a single VRC01 sensitive virus isolate (TZM.bl assay); (2) determine whether anti-idiotypic antibody (AIA) could be detected and whether there was a correlation of VRC01 levels and AIA levels in serum; (3) determine if measurable levels of VRC01 could be found in genital, rectal, and oral secretions; (4) evaluate the kinetics of in vitro neutralization in mucosal secretions of a single VRC01 sensitive virus isolate; and (5) assess binding of VRC01 to multiple Env proteins.

A total of 249 IV infusions and 208 SC injections of VRC01 were administered in HVTN 104. The infusions and injections were well tolerated, with 28% of infusions and 14% of injections associated with mild pain and/or tenderness. 55% of VRC01 and 50% of placebo recipients experienced mild pain and/or tenderness at the infusion or injection site sometime during the trial. One participant experienced moderate pain and/or tenderness following an SC injection of VRC01. Mild infusion site erythema/induration reactions were reported for 2 VRC01 participants and 1 placebo recipient. Two moderate erythema/induration reactions were reported, 1 following IV VRC01 and 1 following VRC01 SC injection. For 76% of the infusions and injections administered in the trial, no systemic reactogenicity symptoms were reported. 56% of VRC01 and 75% of placebo recipients experienced systemic reactogenicity symptoms following at least one study product administration. These were all graded as mild in the placebo recipients. Among VRC01 recipients who experienced any systemic symptoms, the maximum severity was mild in 70%, moderate in 23%, and severe in 6% (3 participants). The most commonly reported symptoms were malaise/fatigue (30%), headaches (32%), and myalgias (26%).The severe systemic reactogenicity symptoms reported in 3 VRC01 recipients in HVTN 104 are detailed as follows:

- One participant developed severe malaise, myalgia, headache and chills, mild nausea, and moderate arthralgia symptoms within 3 days after the first infusion of study product. The participant had a concomitant AE of laboratory confirmed influenza A infection diagnosed on Day 2 and was treated with ibuprofen. Symptoms resolved by Day 7.

- One participant reported a viral illness AE of moderate intensity beginning 3 days prior to the 9th SC injection (10th study product administration), characterized by nausea and vomiting, sore throat, runny nose but no fevers; a household contact was also ill. At baseline on Day 0 of the 9th SC injection, mild malaise/fatigue was still present. At the early assessment timepoint on Day 0, mild malaise/fatigue was still present and was accompanied by grade 1 nausea. Within 3 days of the injection, the participant developed severe malaise, myalgia, headache, chills, and arthralgia; these all resolved on Day 6, which was the date the AE for viral illness was resolved. There was no reported use of concomitant medications for these symptoms.

- One participant reported severe malaise/fatigue on the day of the first infusion, resolving spontaneously the next day. During this reactogenicity period, this participant also reported a grade 1 headache on Day 1, resolving the next day.

There were no study product discontinuations due to reactogenicity symptoms.

There were 235 AEs occurring in 70 participants (79.5%), with similar rates of occurrence across all treatment groups (81% among VRC01 recipients; 75% in placebo recipients). 74% of AEs were graded as mild, 22.5% as moderate, and 3.4% as severe. Only 9 AEs (3.8% of all AEs) occurring in 8 participants were deemed product-related by the investigators; all were mild and transient. Those occurring following VRC01 administration included elevations of hepatic transaminases (aspartate aminotransferase [AST] and alanine aminotransferase [ALT] in 1 individual), elevated creatinine, neutropenia, localized injection site pruritus, diarrhea, generalized rash, and Varicella zoster infection. No VRC01-related hypersensitivity reactions or Cytokine Release Syndrome symptoms were observed during the study.

Study product administration was discontinued in 8 participants. Reasons for discontinuing study product included: AEs (3 participants, see Section 4.9.1.4), relocation from study site (2), unable to adhere to visit schedule (1), unable to contact (1), and pregnancy (1). In summary, VRC01 was well-tolerated when administered IV or SC.

Figure 4-6 shows individual-specific serum VRC01 levels among per-protocol (PP) participants who received all planned infusions and/or injections within protocol-specified visit windows in HVTN 104. Particularly, Groups 4 and 5 evaluated the dose and intervals selected for the phase 2b efficacy trials.

As measured by ELISA, average (geometric mean) serum VRC01 levels in T1 three days after the first (40 mg/kg) and second (20 mg/kg) IV infusion were 422 and 260 mcg/mL, respectively. Average peak levels 1 hour after the last infusion were 796 mcg/mL. Average trough levels 28 days post infusion were 69 mcg/mL after the first infusion and 51, 46, 45, 43 and 46 mcg/mL after infusions 2-6, respectively. VRC01 remained detectable ($\geq$ 1.1 mcg/mL) in 6/9 (67%) of per protocol participants 12 weeks after the last IV infusion in T1.

These levels are similar to those modeled for the phase 2b efficacy trials (HVTN 704/HPTN 085 and HVTN 703/HPTN 081) and they show the desired overlap in concentrations achieved with the two doses selected for these trials.

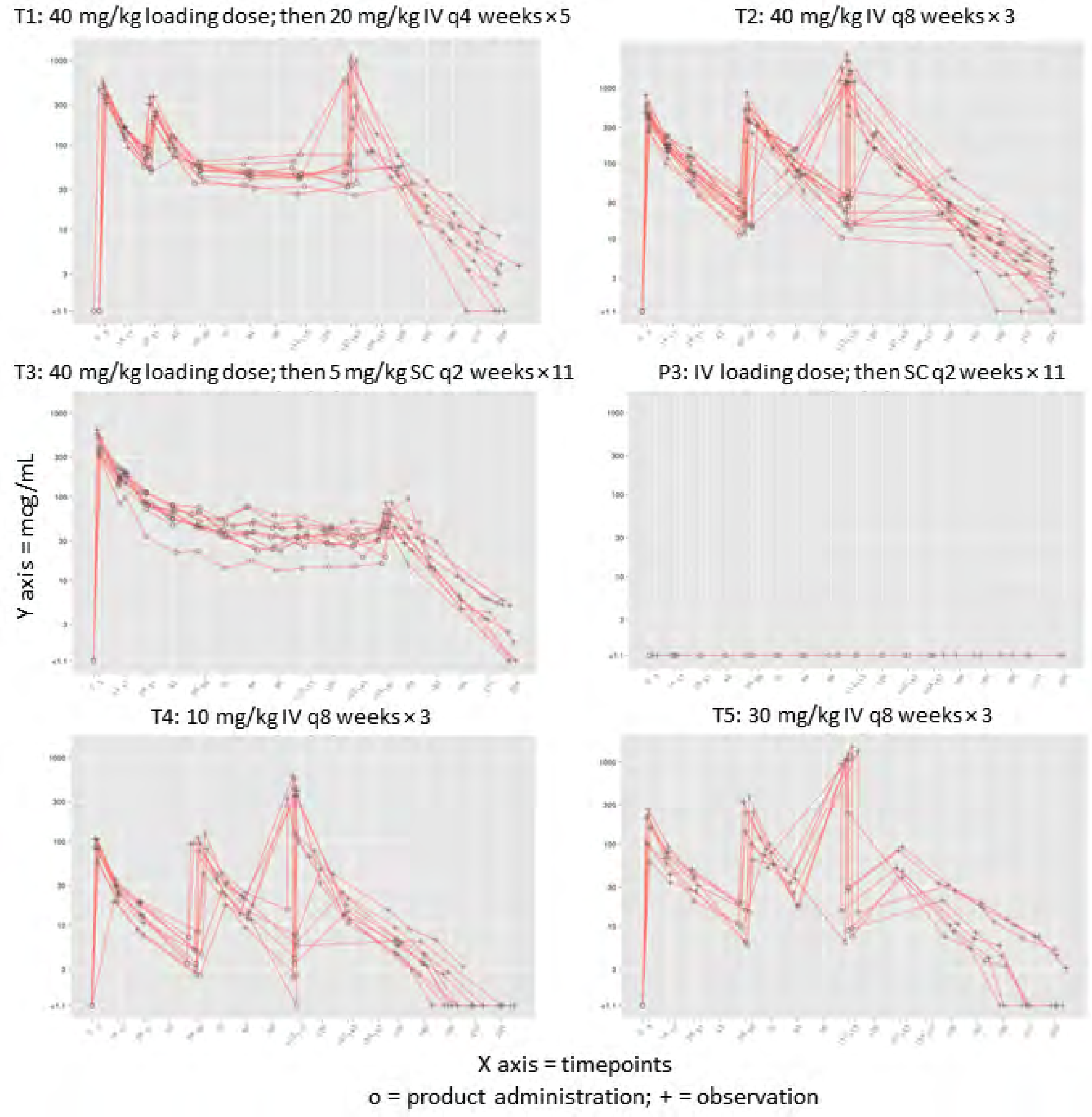

Figure 4-6 Individual-specific VRC01 serum levels among HVTN 104 participants who received all planned infusions/injections of VRC01/placebo. A double tick on the x-axis denotes a time interval of 3 days, representing visit days 3, 17, 31, 59, 115, and/or 157. T1: 20 mg/kg IV, q 4 weeks with 40 mg/kg IV loading; T2: 40 mg/kg IV, q 8 weeks; T3 (P3): 5 mg/kg SC, q 2 weeks with 40 mg/kg IV loading; T4: 10 mg/kg IV, q 8 weeks; T5: 30 mg/kg IV, q 8 weeks. Note: For T1, peak and pre-trough serum concentrations were not measured postinfusion at days 17, 59, 70, 98, 115, 126, 143, 145, and 159; for T3, peak serum concentrations were not measured postinjection at days 31, 59, 115, and 143; for T2, T4, and T5, peak serum concentrations were measured post-third infusion at day 115 only in a subset of participants who also provided mucosal samples.

4.9.1.4 Safety summary of VRC01

As of February 23, 2016, approximately 130 adult participants have received one or more VRC01 administrations in unblinded VRC01 studies, including 23 in VRC 601, 23 in VRC 602 (5 additional participants received placebo), and 84 in HVTN 104 (4 additional participants received placebo). 249 VRC01 infusions and 208 SC injections were given in HVTN 104.

Cumulatively, there have been no expedited safety reports to the FDA or study safety pauses for AEs and no reactions during VRC01 or placebo/control product administration that resulted in an incomplete administration.

VRC01 SC or IV administrations are generally associated with mild or moderate local reactions of pruritus, redness, and pain/tenderness, which resolve within a few minutes to a few hours after the administration is completed. When present, most systemic reactions after administration of VRC01 SC or IV are mild and include malaise, myalgia, headache, chills, nausea, and joint pain.

Unsolicited AEs of grade 3 or higher severity and deemed related to study product were not reported in these trials.

Other AEs attributed to study product administration have included mild or moderate AST elevation, ALT elevation, creatinine elevation, and decreased neutrophil count. Mild or moderate elevated transaminases were reported in 4 of 21 (19%) HIV-infected participants in VRC 601 (all of whom were taking ARVs). These laboratory changes resolved spontaneously and did not require discontinuation of study product administration. Among HIV-uninfected participants in VRC 602 or HVTN 104, 1 participant had grade 1 (mild) transiently elevated ALT assessed as possibly attributed to VRC01 in the VRC 602 study and 2 participants had grade 1 (mild) transiently elevated ALT/AST values assessed as related to VRC01 in HVTN 104. These 3 participants all received VRC01.

In HVTN 104, there were 3 product discontinuations for AEs, 2 of which were deemed related to study product infusion or injection. One discontinuation was for a 20-minute episode of chest tightness occurring approximately 25 minutes after SC injection of placebo in a participant who is a chronic smoker on nicotine replacement while continuing to smoke. One discontinuation was in a participant who reported a generalized rash that began three days after SC injection of VRC01, and resolved within 4 hours with ibuprofen and the application of an inert cream. The third discontinuation was in a young, otherwise healthy participant who experienced a brief episode of syncope, deemed not related to study product, approximately 4 hours after IV infusion of VRC01. In addition, one person in HVTN 104 had study product discontinued due to pregnancy occurring during the trial.

Of note, the safety experience with VRC01 has remained consistent whether 1 or 2 doses were administered, as in VRC 601 and 602, or multiple doses were administered, as in HVTN 104. There were no observed trends toward recurrence of lab abnormalities or AEs deemed related to study product, nor observed increases in frequency or severity of local or systemic reactogenicity symptoms with multiple administrations of VRC01 in these trials.

The ongoing efficacy trials (HVTN 704/HPTN 085 and HVTN 703/HPTN 081) have accumulated significant additional VRC01 clinical experience; but these trials remain blinded and, thus, do not yet contribute to the unblinded VRC01

safety profile. However, in the ongoing blinded phase of these trials, urticaria or similar reactions have been reported at an overall frequency of about 1%. These reactions have been treated by stopping the infusion and providing supportive care, including anti-histamine and steroid therapy, as indicated, and no serious health consequences have been reported. No participants who experienced urticaria have been rechallenged.

Overall, VRC01 administration in the dose range from 10 to 40 mg/kg IV and at 5 mg/kg SC has been assessed as well-tolerated and safe for further evaluation.

4.9.2 Clinical studies of VRC01LS

4.9.2.1 VRC 606

VRC01LS began evaluation in the VRC 606 study in November 2015. As of August 4, 2017, 39 healthy HIV-uninfected subjects had been enrolled and had received a total of 73 administrations of VRC01LS (35 IV and 38 SC) in this dose-escalation study to examine safety, tolerability, dose, and pharmacokinetics of VRC01LS. There were 4 open-label, dose escalation groups (Groups 1-4) to assess VRC01LS administered IV and SC once per participant and 2 open-label groups (Groups 5 and 6) to assess VRC01LS at 5 mg/kg SC or at 20 mg/kg IV administered every 12 weeks for a total of 3 administrations per participant.

VRC01LS SC administrations were sometimes associated with mild local reactions including pain and swelling, which typically resolved within 24 hours. The solicited local and systemic signs and symptoms following administration of VRC01LS were generally none to mild. There have been no SAEs attributed to VRC01LS administration. Grade 1 AEs attributed to study product administration include two cases of diarrhea, one case of dizziness, which resolved without clinical sequelae, one case of erythema at the injection site, and one case of induration at the injection site. No other study product-related AEs have been reported.

Overall, VRC01LS administration in the dose range from 1 to 40 mg/kg IV and at 5 mg/kg SC have been assessed as well-tolerated and safe for further evaluation. An additional study of VRC01LS, HVTN 116 [NCT02797171], is currently recruiting with 36 of 74 participants enrolled as of October 15, 2017.

Serum VRC01LS concentrations as high as 20 mcg/mL were observed in VRC 606 at 165 days (approximately 24 weeks) after a 5 mg/kg IV infusion and were, on average, over 65 mcg/mL at 165 days after a 20 mg/kg IV infusion. VRC01LS concentrations remained, on average, above 10 mcg/mL at 165 days after 5 mg/kg SC (Figure 4-7).

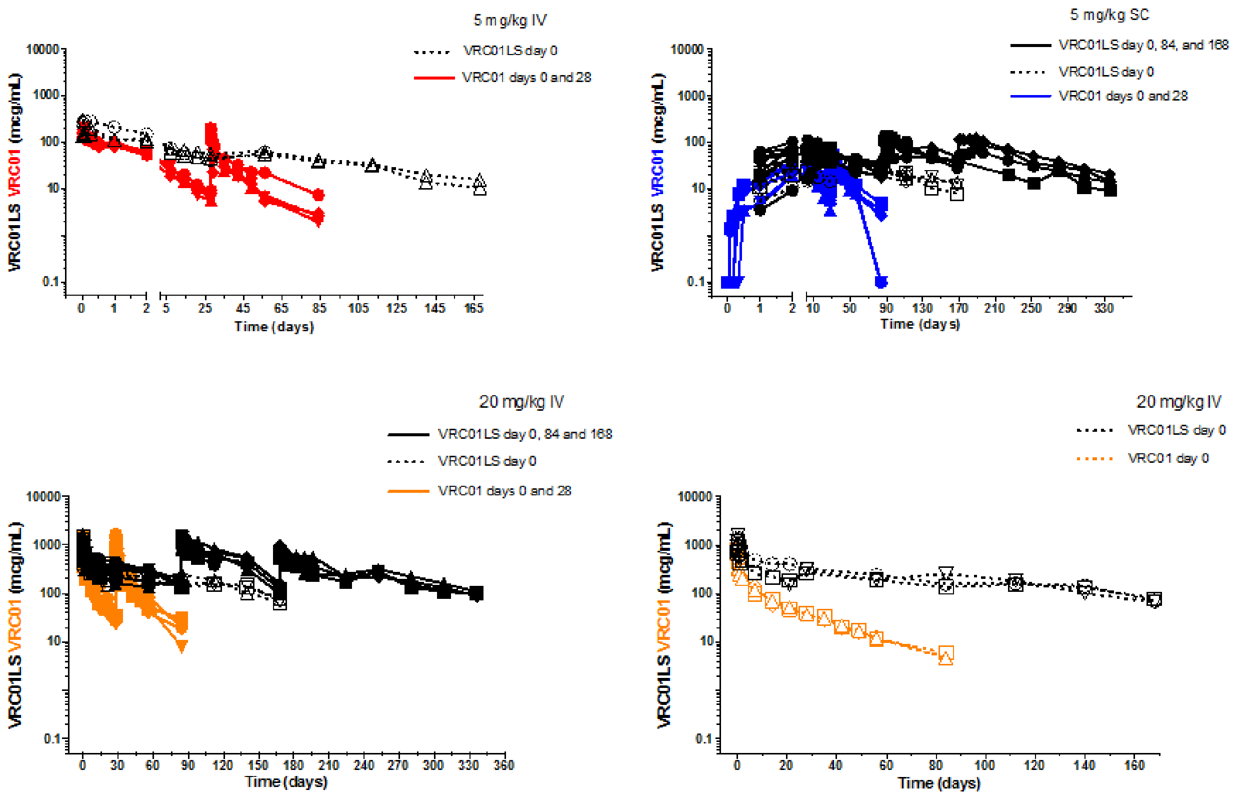

Figure 4-7 VRC01LS serum concentrations in VRC 606 compared to VRC01 serum concentrations with IV and SC administration

Correlation of mAb concentration with neutralization and effector functions is not yet known; however, these levels are considerably higher than seen in VRC01 at similar timepoints. VRC01LS concentration on Day 84 after a single 5mg/kg SC administration was, on average, over 10 times greater than VRC01 concentration on Day 84 after a single 5mg/kg SC VRC01 administration. The difference is even more marked when comparing VRC01 concentrations on Day 84 after a single 20 mg/kg IV infusion to VRC01LS concentrations on Day 84 after a single 20 mg/kg IV infusion: VRC01LS concentrations are over 40 times higher than those of VRC01 (Gaudinski et al, PLoS Medicine, manuscript submitted).

4.9.3 Clinical studies of VRC07-523LS; VRC 605

A phase 1 open-label, dose-escalation study of VRC07-523LS, VRC 605 (NCT03015181), is underway in healthy, HIV-uninfected adults to evaluate the safety and pharmacokinetics of 1 to 3 administrations of the antibody. The doses being evaluated are a single administration of 1 mg/kg and 5 mg/kg IV and SC, and 20 mg/kg and 40 mg/kg IV, and three administrations (q 12 weeks) of 5 mg/kg SC and 20 mg/kg IV VRC07-523LS (Table 4-3).

Study objectives include evaluating the safety and tolerability of the study regimen and the pharmacokinetics of each dose level through 24 weeks after the last dose; determining the presence or absence of detectable ADA to VRC07-523LS; and evaluating for evidence of functional activity of VRC07-523LS.

Table 4-3 VRC 605 study schema

Group	Participants	Administration Schedule		
		Day 0	Week 12	Week 24
1	3	1 mg/kg IV		
2	3	5 mg/kg IV		
3	3	5 mg/kg SC		
4	3	20 mg/kg IV		
5	3	40 mg/kg IV		
6	5	5 mg/kg SC	5 mg/kg SC	5 mg/kg SC
7	5	20 mg/kg IV	20 mg/kg IV	20 mg/kg IV
Total	25			

One participant enrolled into Group 1 then withdrew prior to receiving VRC07-523LS, so an additional participant was enrolled into the group.

As of January 15, 2018, the VRC 605 study is fully enrolled. Twenty-five (25) of 26 participants have received at least 1 dose of VRC07-523LS (12 SC and 25 IV administrations). One participant withdrew prior to receiving the study product. Overall, 15 of 25 participants who received the product (60%) have had at least one AE with the maximum severity being grade 1 for 7 participants, grade 2 for 6 participants, grade 3 for 1 participant and grade 4 for 1 participant. The grade 3 AE was for an elevated creatinine 56 day after the last product administration. This was most likely related to dehydration following exercise. The creatinine value was determined to be a grade 3 based on the DAIDS Table parameter of an increase of 1.5 to 2 times the baseline value, which was still well within the institutional normal range. The grade 4 AE was for elevated liver enzymes likely related to a starting a concomitant medication, fluoxetine, known to cause hepatotoxicity, and not related to VRC07-523LS. VRC07-523LS administrations were discontinued for this participant due to the concomitant illness. While the participant was being followed for safety, liver enzymes tests fluctuated again after starting citalopram which reinforced that the event was most likely caused by an underlying sensitivity to selective serotonin reuptake inhibitor (SSRI) medications. This grade 4 laboratory abnormality was not considered life-threatening as it was not clinically significant as there were no hospitalization, jaundice, coagulopathy, bleeding, or ascites. Six (6) mild or moderate AEs were assessed as related to study product including mild dizziness, 4 occasions of infusion reactions (1 mild and 3 moderate, reported for 2 participants), and mild abdominal pain. All AEs assessed as related to the study product have resolved without residual effects.

Figure 4-8 displays interim VRC07-523LS PK results in VRC 605.

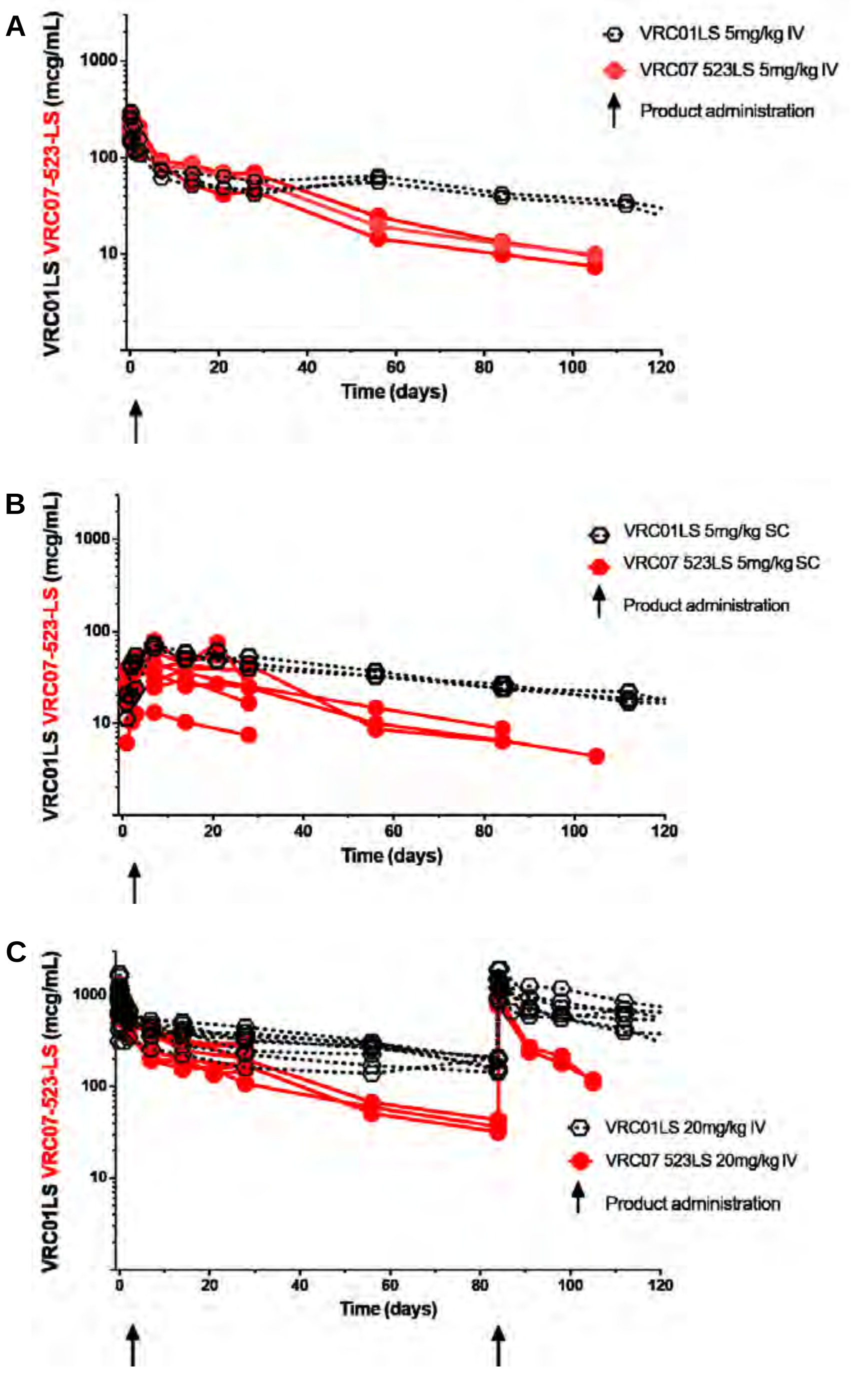

Figure 4-8 Interim VRC 605 PK results for VRC07-523LS with VRC01LS comparators. (A) 5 mg/kg IV (Group 2 in this trial); administrations completed. (B) 5 mg/kg SC (Group 5 in this trial); administrations ongoing. (C) 20 mg/kg IV (Group 3 in this trial); administrations ongoing.

4.10 Potential risks of study products and administration

There is limited human experience with administration of VRC07-523LS. VRC 605 is evaluating VRC07-523LS prior to the start of HVTN 127/HPTN 087 and thus far there have been no SAEs or Grade 3 or higher related AEs. In addition,

the similar CD4-binding site mAb VRC01 has been given to over 2300 participants in several phase 1 and phase 2 clinical trials. More than 8,000 infusions of 10 mg/kg and 30 mg/kg VRC01 have been given to HIV-uninfected adults in HVTN 704/HPTN 085 and HVTN 703/HPTN 081. Both VRC01 and VRC01LS are being tested in an ongoing phase 1 study (HVTN 116).

Standard infusion reactions to mAb administration are typically mild but may include fever, flushing, chills, rigors, nausea, vomiting, pain, headache, dizziness, shortness of breath, hypertension, pruritus, rash, urticaria, diarrhea, tachycardia or chest pain (67). Most infusion reactions appear to result from antibody-antigen interactions resulting in cytokine release (67). Administration of mAbs may have a risk of severe reactions, such as acute anaphylaxis, serum sickness, angioedema, bronchospasm, hypotension, and hypoxia, the generation of anti-drug antibodies; they may also be associated with an increased risk of infections. However, these reactions are rare and more often associated with mAbs targeted to human proteins (67) or with the use of murine mAbs, which have a risk of eliciting human anti-mouse antibodies (68). Infusion of mAbs directed against cell surface targets on lymphocytes may cause a reaction known as “cytokine release syndrome”, with clinical manifestations including fatigue, headache, urticaria, pruritus, bronchospasm, dyspnea, sensation of tongue or throat swelling, rhinitis, nausea, vomiting, flushing, fever, chills, hypotension, tachycardia, and asthenia (69). Cases of cytokine release syndrome occur most often in the first few hours after the first mAb dose, because the cytokine release is associated with lysis of the cells targeted by the mAb and the burden of target cells is greatest at the time of the first mAb treatment (69).

Since VRC07-523LS targets a viral antigen rather than human cell surface antigens and is a human mAb, severe infusion reactions are expected to be rare.

Most infusion-related events occur within the first 24 hours after beginning administration. Delayed allergic reactions to a mAb may include a serum sickness type of reaction, characterized by urticaria, fever, lymph node enlargement, and joint pains. These symptoms may not appear until several days after the exposure to the mAb and are noted to be more common with chimeric types of mAb (68). Reactions related to the rate of infusion have been described for several FDA-licensed mAbs. With licensed therapeutic mAbs, cytokine-mediated infusion reactions, including cytokine release syndrome, are typically managed by temporarily stopping the infusion, administering histamine blockers and restarting the infusion at a slower rate (70). Supportive treatment may also be indicated for some signs and symptoms.

The published experience with mAbs administered by the SC route is limited but there is experience with the SC route of administration of immunoglobulins, such as Hizentra® (CSL Behring LLC, Kankakee, IL), 20% immune serum globulin, for patients with primary immunodeficiency diseases. Comparison of the safety and PK of intravenous immunoglobulin (IVIG) to subcutaneous immunoglobulin (SCIG) has been reported. The SC route of administration has a good safety profile. Tissue reactions are common but usually mild and tend to decline over

time with repeated administrations (71). The package insert for Hizentra® notes that the most common adverse reactions (observed in ≥ 5% of study participants) in clinical trials were local reactions (such as swelling, redness, heat, pain and itching at the infusion site), headache, vomiting, pain, and fatigue.

The published experience with mAb administered by the IM route is also limited but there is experience with the IM route of administration for palivizumab (Synagis®) (72). Monthly IM administration of palivizumab at a dose of 15 mg/kg is safe, well-tolerated, and effective at preventing RSV infection in high-risk infants. Compliance with monthly IM dosing of palivizumab has been reported to be high in clinical practice (73).

To date, the clinical trial safety experience with VRC01-class mAbs has been reassuring.

- In HVTN 104, IV administration of VRC01 was generally well-tolerated with mild pain and/or tenderness commonly reported at the site of the IV infusion. Mild to moderate systemic reactogenicity symptoms were reported by VRC01 recipients following at least one of the infusions, but there was no clear relationship with frequency or severity to the dose of VRC01 (49).

- In HVTN 104, SC administration of VRC01 was generally well-tolerated and associated with mild to moderate local pain and/or tenderness, but there was no difference in the frequency or severity between VRC01 recipients and placebo recipients (49). Erythema and/or induration at the SC injection sites were generally less than 25 cm^2 whether VRC01 or placebo was injected (49).

- No hypersensitivity reactions or cytokine release syndrome symptoms were reported in HVTN 104 (49).

- The ongoing blinded HVTN 704/HPTN 085 and HVTN 703/HPTN 081 trials have reported an approximately 1% rate of urticaria or similar reactions.

- In the ongoing VRC 605 trial of VRC07-523LS, there have been no SAEs and no safety pauses. The maximum severity of AEs has been Grade 1 (for 17 events) and Grade 2 (for 6 events, including 2 infusion reactions).

- Severe reactions associated with mAb administration, such as acute anaphylaxis, serum sickness, anti-drug antibodies, and increased risk of infections have not been observed to date in trials of VRC01-class mAbs.

VRC07-523LS is an antibody to an HIV protein. Therefore, it may be theoretically possible for a standard antibody-based HIV diagnostic test to detect VRC07-523LS for a short time period postinfusion or postinjection. However, in HVTN 104, VRC01 did not cause a positive test result in several standard antibody-based HIV-1/2 diagnostic tests used in the US (49). In vitro testing of blood samples with VRC01 concentrations as high as 1,600 mcg/mL have been evaluated with several common HIV antibody-based test kits and no antibody-

induced seroreactivity has been demonstrated. The issue of seroreactive results to HIV-1/2 screening assays as a result of mAb administration will also be evaluated in the ongoing HVTN 704/HPTN 085 and HVTN 703/HPTN 081 studies to further inform this risk.

Risks of Blood Drawing: Blood drawing may cause pain and bruising and may infrequently cause a feeling of lightheadedness or fainting. Rarely, it may cause infection at the site where the blood is taken. Problems from use of an IV for blood drawing are generally mild and may include pain, bruising, minor swelling or bleeding at the IV site and rarely, infection, vein irritation (called phlebitis), or blood clot. Risk will be minimized by using sterile technique and universal precautions.

Risks of Intravenous Infusion: The placement of an intravenous catheter can allow for the development of bacteremia because of the contact between the catheter and unsterile skin when it is inserted. This will be prevented through careful decontamination of local skin prior to catheter placement and through the use of infection control practices during infusion. Product contamination will be prevented by the use of aseptic technique in the pharmacy and universal precautions during product administration.

5 Objectives and endpoints

5.1 Primary objectives and endpoints

Primary objective 1:

To evaluate the safety and tolerability of different doses of VRC07-523LS administered **IV, SC, and IM** by repeat dosing every 16 weeks for a total of 5 administrations to healthy adults

Primary endpoint 1:

Local and systemic Solicited AEs, laboratory measures of safety, Unsolicited AEs, SAEs, and AEs of special interest (AESI)

Early discontinuation of administration and reason(s) for discontinuation and early study termination

Primary objective 2:

To evaluate the serum concentrations of VRC07-523LS administered IV, SC, and IM over a total of 6 regimens

Primary endpoint 2:

Serum concentrations of VRC07-523LS in each group at specific timepoints from baseline through the final scheduled study visit, as measured by binding activity among participants who received all scheduled product administrations

5.2 Secondary objectives and endpoints

Secondary objective 1:

To develop predictive population pharmacokinetic models of VRC07-523LS administered IV, SC, and IM

Secondary endpoint 1:

Data on serum concentrations of VRC07-523LS at all available timepoints for all groups in all participants regardless of how many product administrations they received

Secondary objective 2:

To determine whether ADA can be detected and whether there is a correlation of VRC07-523LS levels and ADA levels in serum

Secondary endpoint 2:

Serum concentration of ADA in each group measured at multiple timepoints from baseline through the final study visit

Secondary objective 3:

To determine whether serum neutralizing activity is maintained at consistent levels after each product administration

Secondary endpoint 3:

Magnitude and breadth of serum neutralizing activity in each group measured against a panel of Tier 2 viruses that exhibit a range of known sensitivities to VRC07-523LS 8 weeks after each product administration

Secondary objective 4:

To determine whether serum neutralizing activity agrees with measured serum concentrations

Secondary endpoint 4:

Magnitude of neutralization compared to serum concentration and known IC50 of VRC07-523LS for each isolate 8 weeks after each product administration

5.3 Exploratory objectives

Exploratory objective 1:

To evaluate the magnitude and breadth of serum IgG binding activity at each dose level

Exploratory objective 2:

To evaluate additional non-neutralizing functional humoral activities (eg, antibody-dependent cellular cytotoxicity [ADCC], antibody-dependent cellular phagocytosis [ADCP], virion capture) in serum with reagents that have known reactivity with VRC07-523LS

Exploratory objective 3:

To conduct analyses related to furthering the understanding of HIV, monoclonal antibodies, immunology, vaccines, and clinical trial conduct

6 Statistical considerations

6.1 Accrual and sample size calculations

Recruitment will target enrolling 124 healthy, HIV-uninfected adult participants.

The study design includes 6 treatment groups. Table 3-1 provides an overview of the sample sizes, routes of administration, doses, and schedules of administration for these treatment groups. All groups will be open to enrollment at the same time. Participants will be randomly assigned to the 6 groups.

Groups 1, 2, and 3 entail multiple IV administrations of one of three doses of VRC07-523LS (2.5, 5, and 20 mg/kg, respectively). Participants will receive one administration of VRC07-523LS every 16 weeks, for a total of 5 administrations. The first and last administrations will be delivered at baseline (Week 0) and at Week 64 post-baseline, respectively. Recruitment will target 20 participants in each treatment group.

Groups 4, and 5 entail multiple SC administrations of one of two doses of VRC07-523LS (2.5 mg/kg in Group 4, and 5 mg/kg in Group 5). Participants will receive one administration of VRC07-523LS every 16 weeks, for a total of 5 administrations. The first and last administrations will be delivered at baseline (Week 0) and at Week 64 post-baseline, respectively. Recruitment will target 20 participants in each treatment group.

Group 6 entails multiple IM administrations of either one dose level of VRC07-523LS (2.5 mg/kg) or a placebo. Participants will receive one administration every 16 weeks, for a total of 5 administrations. The first and last administrations will be delivered at baseline (Week 0) and at Week 64 post-baseline, respectively. Recruitment will target 24 participants in this treatment group, and use a randomization ratio of 5:1 to assign 20 participants to the VRC07-523LS arm and 4 participants to the placebo arm.

Participants will be followed for 112 weeks from the day they receive their first product administration.

To ensure that both men and women will be adequately represented in the trial, the trial will enroll at least approximately 40% of each sex assigned at birth overall.

Since enrollment is concurrent with receiving the first study product administration, all participants will provide some safety data. However, for PK and functional analyses, it is possible that data may be missing for various reasons, such as participants terminating from the study early, problems in shipping specimens, low cell viability of processed peripheral blood mononuclear cells (PBMCs) or high assay background. Immunogenicity data from 17 phase 1 and 2 phase 2a HVTN vaccine trials, which began enrolling after June 2005 (data

as of September 2014), indicate that 17% is a reasonable estimate for the rate of missing data. In HVTN 104 (phase 1 trial of VRC01), approximately 10-15% of drug concentration data were missing at the primary timepoints. Therefore, the sample size calculations in Section 6.1.2 account for 10-20% enrolled participants having missing data for the primary endpoint.

6.1.1 Sample size calculations for safety

The goal of the safety evaluation for this study is to identify safety concerns associated with product administration. The ability of the study to detect SAEs (see Section 11.2.3) can be expressed by the true event rate above which at least 1 SAE would likely be observed and the true event rate below which no events would likely be observed. Specifically, for each treatment group of size n = 20, there is at least 90% chance of observing at least 1 event if the true rate of such an event is 10.9% or more; and there is at least 90% chance of observing no events if the true rate is 0.5% or less. For active IV administration arms (n = 60) combined, there is at least 90% chance of observing at least 1 event if the true rate of such an event is 3.8% or more; and there is at least 90% chance of observing no events if the true rate is 0.17% or less. For active SC administration arms (n = 40) combined, there is at least 90% chance of observing at least 1 event if the true rate of such an event is 5.6% or more; and there is at least 90% chance of observing no events if the true rate is 0.26% or less. For all active arms combined (n = 120), there is at least 90% chance of observing at least 1 event if the true rate of such an event is 1.91% or more; and there is at least 90% chance of observing no events if the true rate is 0.087% or less. As a reference, in HVTN vaccine trials from December 2000 through April 2014, about 4% of participants who received placebos experienced an SAE.

Binomial probabilities of observing 0, 1 or more, and 2 or more events among arms of sizes 20, 40, 60 and 120 are presented in Table 6-1 for a range of possible true AE rates. These calculations provide a more complete picture of the sensitivity of this study design to identify potential safety problems with VRC07-523LS.

Table 6-1 Probability of observing 0 events, 1 or more events, and 2 or more events, among arms of size 20, 40, 60, and 120 for a range of true event rates

True event rate (%)	arm size	0 events	1+ events	2+ events
1	20	0.82	0.18	0.02
	40	0.67	0.33	0.06
	60	0.55	0.45	0.12
	120	0.30	0.70	0.34
4	20	0.44	0.56	0.19
	40	0.20	0.80	0.48
	60	0.09	0.91	0.70
	120	<0.01	>0.99	0.96
10	20	0.12	0.88	0.61
	40	0.01	0.99	0.92
	60	<0.01	>0.99	0.99
	120	<0.01	>0.99	>0.99
20	20	0.01	0.99	0.93
	40	<0.01	>0.99	>0.99
	60	<0.01	>0.99	>0.99
	120	<0.01	>0.99	>0.99
30	20	<0.01	>0.99	0.99
	40	<0.01	>0.99	>0.99
	60	<0.01	>0.99	>0.99
	120	<0.01	>0.99	>0.99

An alternative way of describing the statistical properties of the study design is in terms of the 95% confidence interval for the true rate of an AE based on the observed data. Table 6-2 shows the 2-sided 95% confidence intervals for the probability of an event based on a particular observed rate. Calculations are done using the score test method (74). If none of the 20 participants for each individual treatment group experience a safety event, the 95% 2-sided upper confidence bound for the true rate of such events in the total VRC07-523LS-treated population is 16.1%.

Table 6-2 Two-sided 95% confidence intervals based on observing a particular rate of safety endpoints for arms of size 20, 40, 60, and 120

Observed event rate	95% Confidence interval (%)
0/20	[0 ; 16.1]
1/20	[0.89 ; 23.6]
2/20	[2.79 ; 30.1]
0/40	[0 ; 8.76]
1/40	[0.44 ; 12.9]
2/40	[1.38; 16.5]
0/60	[0 ; 6.02]
1/60	[0.30 ; 8.9]
2/60	[0.92; 11.4]
0/120	[0 ; 3.1]
1/120	[0.2 ; 4.6]
2/120	[0.15 ; 5.9]

6.1.2 Sample size calculations for serum levels of VRC07-523LS

Primary Objective 2 of this study is to evaluate serum concentrations of VRC07-523LS at several timepoints following multiple IV, SC, and IM administrations. This objective is descriptive in nature, and will be accomplished by estimating the mean VRC07-523LS serum concentration within each active arm at specific timepoints following administration. The precision with which a true mean concentration can be estimated from observed data depends on the standard deviation (SD) of the measurements and the sample size. Table 6-3 displays two-sided 95% confidence intervals for the mean drug concentration for several values of the observed average drug concentration. The construction of these confidence intervals assumed sample sizes of n = 16, 18, and 20 per arm, reflecting an attrition rate of 20%, 10%, and 0% compared to a planned treatment group size of 20 participants. The calculations assumed that log-transformed serum concentrations are approximately normally distributed. For instance, with an observed mean $\log_e$ serum level of $\log_e(5)$ mcg/mL and assuming a standard deviation of 0.5 for their log-transformed values, a two-sided 95% confidence interval for the true mean drug concentration level is (3.8 , 6.5) (in mcg/mL) with an effective sample size of 16 participants.

Table 6-3 Two-sided 95% confidence intervals based on observing a particular average drug level in participants in any of the active arms, taking attrition into consideration (n = 16, 18, 20)

Observed average $\log_e$-serum level ($\log_e$ mcg/mL)	SD of $\log_e$-drug levels ($\log_e$ mcg/mL)	95% confidence interval (mcg/mL)		
		n = 16	n = 18	n = 20
$\log_e(0.5)$	0.5	(0.4 , 0.7)	(0.4 , 0.6)	(0.4 , 0.6)
$\log_e(1)$		(0.8 , 1.3)	(0.8 , 1.3)	(0.8 , 1.3)
$\log_e(3)$		(2.3 , 3.9)	(2.3 , 3.8)	(2.4 , 3.8)
$\log_e(5)$		(3.8 , 6.5)	(3.9 , 6.4)	(4.0 , 6.3)
$\log_e(10)$		(7.7 , 13.1)	(7.8 , 12.8)	(7.9 , 12.6)
$\log_e(20)$		(15.3 , 26.1)	(15.6 , 25.7)	(15.8 , 25.3)
$\log_e(30)$		(23.0 , 39.1)	(23.4 , 38.5)	(23.7 , 37.9)
$\log_e(40)$		(30.6 , 52.2)	(31.2 , 51.3)	(31.6 , 50.6)
$\log_e(50)$		(38.3, 65.3)	(39.0, 64.1)	(39.6, 63.2)
$\log_e(100)$		(76.6, 130.5)	(78.0, 128.2)	(79.1, 126.4)
$\log_e(250)$		(191.5, 326.3)	(195.0, 320.6)	(197.8, 315.9)
$\log_e(500)$		(383.1, 652.6)	(389.9, 641.1)	(395.7, 631.8)
$\log_e(1000)$		(766.1, 1305.3)	(779.9, 1282.3)	(791.4, 1263.7)
$\log_e(0.5)$	1	(0.3 , 0.9)	(0.3 , 0.8)	(0.3 , 0.8)
$\log_e(1)$		(0.6 , 1.7)	(0.6 , 1.6)	(0.6 , 1.6)
$\log_e(3)$		(1.8 , 5.1)	(1.8 , 4.9)	(1.9 , 4.8)
$\log_e(5)$		(2.9 , 8.5)	(3.0 , 8.2)	(3.1 , 8.0)
$\log_e(10)$		(5.9 , 17.0)	(6.1 , 16.4)	(6.3 , 16.0)
$\log_e(20)$		(11.7 , 34.1)	(12.2 , 32.9)	(12.5 , 31.9)
$\log_e(30)$		(17.6 , 51.1)	(18.3 , 49.3)	(18.8 , 47.9)
$\log_e(40)$		(23.5 , 68.2)	(24.3 , 65.8)	(25.0 , 63.9)
$\log_e(50)$		(29.3, 85.2)	(30.4, 82.2)	(31.3, 79.8)
$\log_e(100)$		(58.7, 170.4)	(60.8, 164.4)	(62.6, 159.7)
$\log_e(250)$		(146.7, 426.0)	(152.0, 411.1)	(156.6, 399.2)
$\log_e(500)$		(293.5, 851.9)	(304.1, 822.1)	(313.1, 798.4)
$\log_e(1000)$		(586.9, 1703.8)	(608.2, 1644.3)	(626.2, 1596.8)

The values of the observed average and SD of the $\log_e$ serum concentrations reported in Table 6-3 were selected to cover the range of values that we expect in the proposed study and chosen on the basis of model-based predictions of serum concentration data following multiple IV and SC administrations of VRC07-523LS. These predictions were generated through Monte Carlo simulations of a population PK model adapted from a previously developed population PK model of VRC01 serum concentrations (48, 49). In these simulations, the PK parameters (eg, absorption and distribution rates) of VRC07-523LS were assumed to be all identical to those of VRC01 with the exception of the terminal elimination rate which was set to either 1/2, 1/5, or 1/10 of that of VRC01 (estimated half-life = 15 days). These assumptions were motivated by experimental observations in preclinical macaque studies with VRC07-523LS and VRC 606 using VRC01LS which suggested that the half-life of VRC07-523LS could be approximately at least twice as long as that of VRC01. Thus, 3 scenarios were considered in these

simulations in which the terminal half-life of VRC07-523LS was set to either 30 days, or 75 days, or 150 days. We simulated the serum concentration trajectories of 4,000 hypothetical individuals using the population PK model. Because body weight was found to be significantly associated with some of the PK parameters of VRC01 (eg, its clearance) in the HVTN 104 study, the body weights of the 4,000 hypothetical individuals were randomly drawn with replacement from the sample of body weights collected in the HVTN 104 study. The mean and variance of log-transformed serum concentrations were next computed up to 48 weeks after the last administration of VRC07-523LS for the 20 mg/kg IV and 2.5 mg/kg SC treatment groups proposed in this study (Groups 3 and 4). For example, assuming a half-life of 30 days for VRC07-523LS, the model predicted mean $\log_e$-serum concentrations at 24 weeks post last IV and SC administration in Groups 3 and 4 of $\log_e(2.4)$ and $\log_e(0.7)$ mcg/mL, respectively. Assuming a half-life of 75 days for VRC07-523LS, the predicted mean $\log_e$-serum concentrations at 24 weeks post last IV and SC administration in Groups 3 and 4 were estimated as $\log_e(33.2)$ and $\log_e(3.7)$ mcg/mL, respectively. Assuming a half-life of 150 days for VRC07-523LS, the predicted mean serum concentrations at 24 weeks post the last IV and SC administration in Groups 3 and 4 were estimated as $\log_e(102.0)$ and $\log_e(12.4)$ mcg/mL, respectively. The scenarios presented in Table 6-3 are expected to cover the range of values of VRC07-523LS serum concentrations that will be measured at the sampling times in Groups 1 - 5.

Serum concentration data following IM administrations of 2.5 mg/kg VRC07-523LS (Group 6) were not simulated due to the lack of a population PK model describing this route of administration in a related product. The mean and variance of serum concentrations are expected to fall within the range of values predicted for serum concentration data following IV and SC administrations of 2.5 mg/kg VRC07-523LS (Groups 1 and 4).

6.2 Randomization

A participant's randomization assignment will be computer generated and provided to the CRS pharmacist through a Web-based randomization system. All groups will be enrolled simultaneously. The randomization will be done in blocks. This will ensure balanced randomization across Groups 1 through 5, with proper weights used to accelerate enrollment in Group 6 relative to Groups 1 through 5 so that enrollment progresses concurrently in all arms.

At each institution, the pharmacist with primary responsibility for dispensing study products is charged with maintaining security of the treatment assignments (except in emergency situations as specified in the Study Specific Procedures [SSP]).

6.3 Blinding

Participants and CRS staff will be unblinded to participant group assignments (Groups 1 through 6); and to participant treatment assignments (Groups 1 through 5).

In Group 6, participants and CRS staff (except for CRS pharmacists) will be blinded to treatment assignment (ie, VRC07-523LS versus placebo). As the differing viscosities of VRC07-523LS and normal saline risk unblinding CRS staff (despite the use of syringe overlays [Section 8.3.1.3]), Group 6 participants will have study product administered and AE assessment/reporting performed by different CRS staff members. Study product assignments are accessible to those CRS pharmacists, DAIDS protocol pharmacists and contract monitors, and SDMC staff who are required to know this information in order to ensure proper trial conduct. Any discussion of study product assignment between pharmacy staff and any other CRS staff is prohibited.

The SMB members are unblinded to treatment assignment for all 6 groups in order to conduct review of trial safety.

If a participant in Group 6 leaves the trial prior to study completion, the participant will be told he or she must wait until all Group 6 participants are unblinded to learn his or her treatment assignment.

Emergency unblinding decisions will be made by the site investigator. If time permits, the HVTN 127/HPTN 087 PSRT should be consulted before emergency unblinding occurs.

6.4 Statistical analyses

This section describes the final study analysis, unblinded as to group and treatment arm assignment.

Analyses for primary endpoints will be performed using SAS and R. All other descriptive and inferential statistical analyses will be performed using SAS, StatXact, or R statistical software. Additional software may be used to perform population PK analyses (eg, NONMEM).

No formal multiple comparison adjustments will be employed for multiple primary endpoints. However, multiplicity adjustments will be made for certain secondary endpoints or assay data when the endpoint is viewed as a collection of hypotheses (eg, testing multiple peptide pools to determine a positive response; comparison of PK parameters across multiple treatment groups).

6.4.1 Analysis variables

The analysis variables consist of baseline participant characteristics, safety, VRC07-523LS serum concentrations, ADA, and drug functionality for primary- and secondary-objective analyses.

6.4.2 Baseline comparability

Treatment arms will be compared for baseline participant characteristics using descriptive statistics.

6.4.3 Safety/tolerability analysis

All data from enrolled participants will be analyzed according to the initial randomization assignment regardless of how many administrations they received. The analysis is a modified intent-to-treat analysis (MITT) in that individuals who are randomized but not enrolled do not contribute data and hence are excluded from subsequent analysis.

Since enrollment is concurrent with receiving the first study product administration, all participants will have received at least 1 study product administration and therefore will provide some safety data.

6.4.3.1 Solicited AEs

The number and percentage of participants experiencing each type of Solicited AE sign or symptom will be tabulated by severity, attribution and treatment arm and the percentages displayed graphically by arm. For a given sign or symptom, each participant's Solicited AEs will be counted once under the maximum severity for all infusion/injection visits. In addition to the individual types of events, the maximum severity of local pain or tenderness, induration or erythema, and of systemic symptoms will be calculated. Appropriate tests (eg, Kruskal-Wallis or Wilcoxon rank-sum tests) will be used to test for differences in severity between arms.

6.4.3.2 SAEs and Unsolicited AEs

Unsolicited AEs will be summarized using MedDRA System Organ Class and preferred terms. Tables will show by treatment arm the number and percentage of participants experiencing an Unsolicited AE within a System Organ Class or within preferred term category by severity or by relationship to study product. For the calculations in these tables, a participant with multiple Unsolicited AEs within a category will be counted once under the maximum severity or the strongest recorded causal relationship to study product. Formal statistical testing comparing arms is not planned since interpretation of differences must rely heavily upon clinical judgment.

A listing of SAEs reported to the DAIDS Regulatory Support Center (RSC) Safety Office will provide details of the events including severity, relationship to

study product, time between onset and last study product administration, and number of study product administrations received. A separate listing will do the same for AESI. A list of AESI to be reported for this protocol is provided in Appendix H.

6.4.3.3 Local laboratory values

Box plots of local laboratory values will be generated for baseline values and for values measured during the course of the study by treatment arm and visit. Each box plot will show the first quartile, the median, and the third quartile. Outliers (values outside the box plot) will also be plotted. If appropriate, horizontal lines representing boundaries for abnormal values will be plotted.

For each local laboratory measure, summary statistics will be presented by treatment arm and timepoint, as well as changes from baseline for postenrollment values. In addition, the number (percentage) of participants with local laboratory values recorded as meeting Grade 1 AE criteria or above as specified in the Division of AIDS Table for Grading the Severity of Adult and Pediatric Adverse Events (see Section 11.2.2) will be tabulated by treatment arm for each poststudy product administration timepoint. Reportable clinical laboratory abnormalities without an associated clinical diagnosis will also be included in the tabulation of AEs described above.

6.4.3.4 Reasons for study product discontinuation and early study termination

The number and percentage of participants who discontinue study product administration and who terminate the study early will be tabulated by reason and treatment arm.

6.4.4 Serum concentration and population PK analysis of VRC07-523LS

6.4.4.1 General approach

The primary goal of the statistical analyses is to document serum concentrations of VRC07-523LS over time following multiple IV, SC, and IM administrations.

The primary analyses of serum concentrations of VRC07-523LS (Primary Objective 2) will be restricted to participants who received all scheduled administrations per protocol. Serum concentration data that appear unreliable, or from specimens collected outside of the visit window, or from HIV-infected participants postinfection may be excluded.

The primary analysis of serum concentrations of VRC07-523LS will be performed using univariate statistics (eg, sample mean, standard deviation, standard error for the mean). Data will be displayed graphically using appropriate visualization techniques (eg, boxplots, histograms, spaghetti plots). Summary statistics and visualizations will be produced per treatment group and per timepoint.

Additional analyses, addressing Secondary Objective 1, will estimate population PK parameters of VRC07-523LS, compare PK parameters across treatment groups, and assess whether any associations exist between PK parameters and participant's baseline covariates (eg, body weight, age, or gender). Data from enrolled participants will be analyzed according to the initial randomization assignment regardless of how many administrations they received (MITT analysis). Serum concentration data that appear unreliable, or from HIV-infected participants postinfection may be excluded. Serum concentration data from specimens collected outside of the visit window may be included in population PK analysis that account for the actual specimen collection time and the actual time of each product administration. Since the exact date of HIV infection is unknown, any serum level data from blood draws 4 weeks prior to an infected participant's last seronegative sample and thereafter may be excluded. All data from HIV-infected participants who have no seronegative samples postenrollment may be excluded from the analysis.

The objective of the analysis of Secondary Objective 1 is to develop a population PK model of VRC07-523LS serum concentrations in healthy, HIV-uninfected adults. This analysis will be conducted using a population PK approach. The proposed models will describe the pharmacokinetics of VRC07-523LS at the individual level using a compartmental approach. Based on a previous population PK analysis of the serum concentrations of VRC01, we anticipate that a two-compartmental model can characterize the kinetics of the serum concentrations of VRC07-523LS. In the event the modeling assumptions appear violated, we will consider other compartmental models. Serum concentrations from study participants will be described using a non-linear mixed effects model. Comparisons of PK parameters across treatment groups, doses, or routes of administration, will be performed using either likelihood ratio or Wald tests. Additional exploratory analyses will be performed to identify baseline covariates potentially associated with PK parameters of VRC07-523LS. Estimates of PK parameters, including area-under-the-curve (AUC), maximum concentration (Cmax), time to Cmax (Tmax), clearance (CL), volume of distribution (V), elimination rates and half-life, will be derived from these analyses.

6.4.5 ADA and drug functionality analysis

The goal of the statistical analyses for the Secondary Objectives 2 – 4 and for the Exploratory Objectives will be to document drug functionality endpoints following multiple IV, SC, and IM administrations of VRC07-523LS, and to assess whether any association exists between ADA and VRC07-523LS serum levels.

For Secondary Objective 2 regarding ADA, data from enrolled participants will be used according to the initial randomization assignment regardless of how many administrations they received (MITT). For Secondary Objectives 3 and 4 regarding drug functionality, data from enrolled participants who received all scheduled administrations per-protocol will be used. Assay results that are unreliable or from HIV-infected participants postinfection will be excluded.

Univariate and bivariate descriptive analyses of continuous assay data (eg, serum concentration of VRC07-523LS) will be performed using mean, median, standard deviation, range, skewness, Spearman's and Pearson's correlation coefficients, for example. Graphical displays of the data based on appropriate techniques (eg, boxplots, histograms, kernel density estimates, probability plots, two- or three-dimensional scatterplots, spaghetti plots) will be generated to visually explore distributional properties of the data as well as potential pairwise associations. Statistics and graphical displays will be produced for each treatment arm across timepoints.

Comparisons of continuous assay data between treatment groups or timepoints will be primarily performed using nonparametric rank-based tests (eg, Wilcoxon rank-sum test, Friedman nonparametric two-way analysis of variance (ANOVA). In the event the data appear normally distributed, the results of these tests may be compared to those produced by parametric tests (eg, two-sample *t*-tests with unequal variances). Appropriate data transformations (eg, square-root, logarithmic) may be applied prior to testing hypotheses in order for key distributional assumptions [eg, normality, homoscedasticity (ie, constancy of variance)] to be satisfied.

Analysis of longitudinal data may be performed using mixed effects models or generalized estimating equations (GEE). These approaches allow describing outcome responses over several timepoints while accounting for potential inter-subject heterogeneity.

Analyses of categorical variables (eg, binary) will be conducted by constructing frequency tables. One such table will be produced for each treatment group and each timepoint. Crude response rates will be presented with their corresponding 95% confidence interval estimates calculated using the score test method (74). Associations between categorical variables will be assessed using Fisher's exact or Chi-squared tests.

To achieve unbiased statistical estimation and inferences with nonparametric tests and generalized linear models fit by GEE methods, missing data need to be missing completely at random (MCAR). MCAR assumes that the probability of an observation being missing does not depend upon the observed responses or upon any unobserved covariates but may depend upon covariates included in the model (eg, missing more among whites than nonwhites). In situations where data are missing in no more than 20% of the participants, we will use nonparametric tests and GEE methods on which violations of the MCAR assumption are expected to have limited impact. We will consider including any of the available baseline predictors of the missing outcomes as covariates in statistical models.

When the frequency of missing data is more substantial, methods that require the MCAR assumption may give misleading results. In this situation, statistical analyses will be performed using parametric generalized linear models fitted using the method of maximum likelihood which provides unbiased estimation and inferences under the parametric modeling assumptions and the assumption that

the missing data are missing at random (MAR). MAR assumes that the probability of an observation being missing may depend upon the observed responses and upon observed covariates, but not upon any unobserved factors. Thus, this assumption is less stringent than the MCAR assumption. Generalized linear models for response rates will use a binomial error distribution and for quantitative endpoints, a normal error distribution. We will assess repeated immunogenicity measurement using linear mixed effects models. If immunological outcomes are left- and/or right- censored, we will use Hughes' (75) linear mixed effects models to accommodate censoring. In addition, secondary analyses of repeated immunogenicity measurements may be done using weighted GEE (76) methods, which are valid under MAR. We will again consider including any of the available baseline predictors of the missing outcomes as covariates in statistical models.

6.4.6 Analyses and data sharing prior to end of scheduled follow-up visits

Any analyses conducted prior to the end of the scheduled follow-up visits should not compromise the integrity of the trial in terms of participant retention or safety endpoint assessments. In particular, early analyses by treatment assignment require careful consideration and should be made available on a need to know basis in accordance with Section 6.4.6.1. Interim blinded safety data should not be shared outside of the SMB, HVTN 127/HPTN 087 PSRT, the protocol team leadership, the study product developer, and the study sponsor and/or its designee(s) for their regulatory reporting unless approved by the protocol and Networks' leadership.

6.4.6.1 Safety analyses

During the course of the trial, unblinded analyses of safety data will be prepared approximately every 4 months for review by the SMB. Ad hoc safety reports may also be prepared for SMB review at the request of the HVTN 127/HPTN 087 PSRT. The HVTN and HPTN leadership must approve any other requests for unblinded safety data prior to the end of the scheduled follow-up visits.

7 Selection and withdrawal of participants

Participants will be healthy, HIV-uninfected (seronegative) adults who comprehend the purpose of the study and have provided written informed consent. Volunteers will be recruited and screened; those determined to be eligible, based on the inclusion and exclusion criteria, will be enrolled in the study. Final eligibility determination will depend on information available at the time of enrollment, including results of screening laboratory tests, medical history, physical examinations, and answers to self-administered and/or interview questions.

Investigators should always use good clinical judgment in considering a volunteer's overall fitness for trial participation. Some volunteers may not be appropriate for enrollment even if they meet all inclusion/exclusion criteria. Medical, psychiatric, occupational, or other conditions may make evaluation of safety and/or immunogenicity difficult, and some volunteers may be poor candidates for retention.

Determination of eligibility, taking into account all inclusion and exclusion criteria, must be made within 56 days prior to enrollment unless otherwise noted in Sections 7.1 and 7.2.

7.1 Inclusion criteria

General and Demographic Criteria

1. **Age** of 18 to 50 years
2. **Access to a participating CRS** and willingness to be followed for the planned duration of the study
3. Ability and willingness to provide **informed consent**
4. **Assessment of understanding**: volunteer demonstrates understanding of this study and completes a questionnaire prior to first study product administration with verbal demonstration of understanding of all questionnaire items answered incorrectly
5. **Agrees not to enroll in another study** of an investigational research agent until completion of the last required protocol clinic visit
6. **Good general health** as shown by medical history, physical exam, and screening laboratory tests

HIV-Related Criteria:

7. Willingness to receive **HIV test results**

8. Willingness to discuss HIV infection risks and amenable to HIV risk reduction counseling.

9. Assessed by the clinic staff as being at 'low risk' for HIV infection and committed to maintaining behavior consistent with those criteria through the last required protocol clinic visit (see Appendix I).

Laboratory Inclusion Values

Hemogram/CBC

10. **Hemoglobin** ≥ 11.0 g/dL for volunteers who were assigned female sex at birth, ≥ 13.0 g/dL for volunteers who were assigned male sex at birth. For transgender participants who have been on hormone therapy for more than 6 consecutive months, determine hemoglobin eligibility based on the gender with which they identify (ie, a transgender female who has been on hormone therapy for more than 6 consecutive months should be assessed for eligibility using the hemoglobin parameters for volunteers assigned female sex at birth).

11. **White blood cell count** = 2,500 to 12,000 cells/mm^3

12. **WBC differential** either within institutional normal range or with site physician approval

13. **Platelets** = 125,000 to 550,000/mm^3

Chemistry

14. **Chemistry panel**: **ALT** < 1.25 times the institutional upper limit of normal and **creatinine** ≤ institutional upper limits of normal.

Virology

15. **Negative HIV-1 and -2 blood test**: US volunteers must have a negative FDA-approved enzyme immunoassay (EIA) or chemiluminescent microparticle immunoassay (CMIA). Non-US sites may use locally available assays that have been approved by HVTN and HPTN Laboratory Operations.

16. **Negative Hepatitis B surface antigen (HBsAg)**

17. **Negative anti-Hepatitis C virus antibodies (anti-HCV)**, or negative HCV polymerase chain reaction (PCR) if the anti-HCV is positive

Urine

18. **Negative or trace urine protein**

Reproductive Status

19. **Volunteers who were assigned female sex at birth**: negative serum or urine beta human chorionic gonadotropin (β-HCG) pregnancy test performed prior to study product administration on the day of initial study infusion/injection. Persons who are NOT of reproductive potential due to having undergone total hysterectomy or bilateral oophorectomy (verified by medical records), are not required to undergo pregnancy testing.

20. **Reproductive status**: A volunteer who was assigned female sex at birth must:

    - Agree to use effective contraception (see Appendix B) for sexual activity that could lead to pregnancy from at least 21 days prior to enrollment through the last required protocol clinic visit. Effective contraception is defined as using the following methods:
        - Condoms (male or female) with or without a spermicide,
        - Diaphragm or cervical cap with spermicide,
        - Intrauterine device (IUD),
        - Hormonal contraception, or
        - Any other contraceptive method approved by the HVTN 127/HPTN 087 PSRT
        - Successful vasectomy in any partner assigned male sex at birth (considered successful if a volunteer reports that a male partner has [1] documentation of azoospermia by microscopy, or [2] a vasectomy more than 2 years ago with no resultant pregnancy despite sexual activity postvasectomy);
    - Or not be of reproductive potential, such as having reached menopause (no menses for 1 year) or having undergone hysterectomy, bilateral oophorectomy, or tubal ligation;
    - Or plan to be sexually abstinent until at least 6 months following the last study product administration.

21. **Volunteers who were assigned female sex at birth must also agree not to seek pregnancy through alternative methods**, such as artificial insemination or in vitro fertilization until after the last required protocol clinic visit

7.2 Exclusion criteria

General

1. **Weight > 115 kg**

2. **Blood products** received within 120 days before first study product administration, unless eligibility for earlier enrollment is determined by the HVTN 127/HPTN 087 PSRT

3. **Investigational research agents** received within 30 days before first study product administration

4. **Intent to participate in another study** of an investigational research agent or any other study that requires non-Network HIV antibody testing during the planned duration of the HVTN 127/HPTN 087 study

5. **Pregnant or breastfeeding**

Vaccines and other Injections

6. **HIV vaccine(s)** received in a prior HIV vaccine trial. For volunteers who have received control/placebo in an HIV vaccine trial, the HVTN 127/HPTN 087 PSRT will determine eligibility on a case-by-case basis.

7. **Previous receipt of humanized or human mAbs,** whether licensed or investigational; the HVTN 127/HPTN 087 PSRT will determine eligibility on a case-by-case basis.

Immune System

8. **Immunosuppressive medications** received within 30 days before first injection or infusion (Not exclusionary: [1] corticosteroid nasal spray; [2] inhaled corticosteroids; [3] topical corticosteroids for mild, uncomplicated dermatitis; or [4] a single course of oral/parenteral prednisone or equivalent at doses < 2 mg/kg/day and length of therapy < 11 days with completion at least 30 days prior to enrollment)

9. **Serious adverse reactions to VRC07-523LS formulation components,** including history of anaphylaxis and related symptoms such as hives, respiratory difficulty, angioedema, and/or abdominal pain

10. **Immunoglobulin** received within 90 days before first injection or infusion, unless eligibility for earlier enrollment is determined by the HVTN 127/HPTN 087 PSRT

11. **Autoimmune disease** (Not excluded from participation: Volunteer with mild, stable and uncomplicated autoimmune disease that does not require immunosuppressive medication and that, in the judgment of the site investigator, is likely not subject to exacerbation and likely not to complicate Solicited and Unsolicited AE assessments)

12. **Immunodeficiency**

Clinically significant medical conditions

13. **Clinically significant medical condition**, physical examination findings, clinically significant abnormal laboratory results, or past medical history with clinically significant implications for current health. A clinically significant condition or process includes but is not limited to:

    - A process that would affect the immune response,
    - A process that would require medication that affects the immune response,
    - Any contraindication to repeated injections, infusions, or blood draws, including inability to establish venous access,
    - A condition that requires active medical intervention or monitoring to avert grave danger to the volunteer's health or well-being during the study period,
    - A condition or process (eg, chronic urticaria or recent injection or infusion with evidence of residual inflammation) for which signs or symptoms could be confused with reactions to the study product, or
    - Any condition specifically listed among the exclusion criteria.

14. **Any medical, psychiatric, occupational, or other condition** that, in the judgment of the investigator, would interfere with, or serve as a contraindication to, protocol adherence, assessment of safety or Solicited AEs, or a volunteer's ability to give informed consent

15. **Psychiatric condition that precludes compliance with the protocol**. Specifically excluded are persons with psychoses within the past 3 years, ongoing risk for suicide, or history of suicide attempt or gesture within the past 3 years.

16. **Current anti-tuberculosis (TB) therapy**

17. **Asthma** other than mild or moderate, well-controlled asthma. (Symptoms of asthma severity as defined in the most recent National Asthma Education and Prevention Program (NAEPP) Expert Panel report).

    Exclude a volunteer who:

    - Uses a short-acting rescue inhaler (typically a beta 2 agonist) daily, or
    - Uses high dose inhaled corticosteroids, or
    - In the past year has had either of the following:
        - Greater than 1 exacerbation of symptoms treated with oral/parenteral corticosteroids;
        - Needed emergency care, urgent care, hospitalization, or intubation for asthma.

18. **Diabetes mellitus** type 1 or type 2 (Not excluded: type 2 cases controlled with diet alone or a history of isolated gestational diabetes.)

19. **Hypertension**:

    - If a person has been found to have elevated blood pressure or hypertension during screening or previously, exclude for blood pressure that is not well controlled. Well-controlled blood pressure is defined as consistently ≤ 140 mm Hg systolic and ≤ 90 mm Hg diastolic, with or without medication, with only isolated, brief instances of higher readings, which must be ≤ 150 mm Hg systolic and ≤ 100 mm Hg diastolic. For these volunteers, blood pressure must be ≤ 140 mm Hg systolic and ≤ 90 mm Hg diastolic at enrollment.

    - If a person has NOT been found to have elevated blood pressure or hypertension during screening or previously, exclude for systolic blood pressure ≥ 150 mm Hg at enrollment or diastolic blood pressure ≥ 100 mm Hg at enrollment.

20. **Bleeding disorder** diagnosed by a doctor (eg, factor deficiency, coagulopathy, or platelet disorder requiring special precautions)

21. **Malignancy** (Not excluded from participation: Volunteer who has had malignancy excised surgically and who, in the investigator's estimation, has a reasonable assurance of sustained cure, or who is unlikely to experience recurrence of malignancy during the period of the study)

22. **Seizure disorder:** History of seizure(s) within past three years. Also exclude if volunteer has used medications in order to prevent or treat seizure(s) at any time within the past 3 years.

23. **Asplenia**: any condition resulting in the absence of a functional spleen

24. History of hereditary **angioedema**, acquired angioedema, or idiopathic angioedema.

7.3 Participant departure from study product administration schedule or withdrawal

This section concerns an individual participant's departure from the study product administration schedule. Pause rules for the trial are described in Section 11.3.

7.3.1 Delaying study product administration for a participant

Under certain circumstances, a participant's scheduled infusion or injection will be delayed. The factors to be considered in such a decision include but are not limited to the following:

- Within 7 days prior to any study product administration

- Receipt of systemic glucocorticoids (eg, prednisone or other glucocorticoids) or other immunomodulators (other than nonsteroidal anti-inflammatory drugs [NSAIDs])

- Preinfusion or preinjection abnormal vital signs or clinical symptoms that may mask assessment of study product reaction.

- Intercurrent illness that is not expected to resolve prior to the next scheduled study product administration which is assessed by the site principal investigator (or designee) to require delay or withdrawal from the study product administration schedule. The investigator may consult the HVTN 127/HPTN 087 PSRT.

- Pregnancy: study product administration will be stopped while a participant is pregnant. If the participant is no longer pregnant or breast-feeding and study product administration can be performed within an appropriate visit window, study product administration may resume with unanimous consent of the HVTN 127/HPTN 087 PSRT.

Infusions and injections should not be administered outside the visit window period specified in the HVTN 127/HPTN 087 SSP.

In order to avoid administration delays and missed injections or infusions, participants who plan to receive systemic glucocorticoids should be counseled to schedule receipt of these substances, when possible, outside the intervals indicated above. The effects of these substances on safety and their interactions with study product are unknown.

7.3.2 Participant departure from study product administration schedule

Every effort should be made to follow the study product administration schedule per the protocol. If a participant misses a study product administration and the visit window period for the study product administration has passed, that study product cannot be given. The participant should be asked to continue study visits. The participant should resume the study product administration schedule with the next study product administration unless there are circumstances that require further delay or permanent discontinuation of study product administration (see Sections 7.3.1 and 7.3.3).

7.3.3 Discontinuing study product administration for a participant

Under certain circumstances, an individual participant's study product administrations will be permanently discontinued. Specific events that will result in stopping a participant's study product administration schedule include:

- Co-enrollment in a study with an investigational research agent (rare exceptions allowing for the continuation of study product administrations may be granted with the unanimous consent of the HVTN 127/HPTN 087 PSRT)

- Clinically significant condition (ie, a condition that affects the immune system or for which continued study product administration and/or blood draws may pose additional risk), including but not limited to the following:
  - Any grade 4 local or systemic Solicited or Unsolicited AE that is subsequently considered to be related to study product;
  - Any grade 3 clinical AE (exception: fever and subjective local and systemic symptoms) that is subsequently considered to be related to study product (upon review, the HVTN 127/HPTN 087 PSRT may allow continuation of study product administration if the participant has grade 3 erythema and/or induration);
  - Any grade 3 or 4 lab abnormality confirmed by a repeated value that is subsequently considered to be related to study product;
  - SAE that is subsequently considered to be related to study product administration; or
  - Clinically significant hypersensitivity or infusion related reaction including but not limited to type 1 hypersensitivity reaction, urticaria, or serum sickness associated with study product. Consultation with the HVTN 127/HPTN 087 PSRT is required prior to subsequent infusion following any hypersensitivity reaction associated with study product; or
- Investigator determination in consultation with Protocol Team leadership (eg, for repeated nonadherence to study staff instructions)

Participants discontinuing study product should be counseled on the importance of continuing with the study and strongly encouraged to participate in follow-up visits and protocol-related procedures per the protocol for the remainder of the trial, unless medically contraindicated.

In addition, study product administration will be stopped for participants diagnosed with HIV infection. HIV-infected participants will not continue in the trial (see Sections 7.3.4).

7.3.4 Participant termination from the study

Under certain circumstances, an individual participant may be terminated from participation in this study. Specific events that will result in early termination include:

- Participant refuses further participation,
- Participant relocates and remote follow-up or transfer to another CRS is not possible,
- CRS determines that the participant is lost to follow-up,
- Participant becomes HIV infected, or

- Investigator decides, in consultation with Protocol Team leadership, to terminate participation (eg, if participant exhibits inappropriate behavior toward clinic staff).

- Any condition where termination from the study is required by applicable regulations.

8 Study product preparation and administration

CRS pharmacists should consult the Pharmacy Guidelines and Instructions for DAIDS Clinical Trials Networks for standard pharmacy operations. The protocol schema is shown in Table 3-1. See the Investigator's Brochure for further information about study products.

8.1 Study product regimen

The schedule of study product administration is shown in Section 3 and additional information is given below.

Group 1

Treatment 1 (T1): VRC07-523LS (VRC-HIVMAB075-00-AB) 2.5 mg/kg to be administered IV at weeks 0, 16, 32, 48, and 64.

Group 2

Treatment 2 (T2): VRC07-523LS (VRC-HIVMAB075-00-AB) 5 mg/kg to be administered IV at weeks 0, 16, 32, 48, and 64.

Group 3

Treatment 3 (T3): VRC07-523LS (VRC-HIVMAB075-00-AB) 20 mg/kg to be administered IV at weeks 0, 16, 32, 48, and 64.

Group 4

Treatment 4 (T4): VRC07-523LS (VRC-HIVMAB075-00-AB) 2.5 mg/kg to be administered SC at weeks 0, 16, 32, 48, and 64.

Group 5

Treatment 5 (T5): VRC07-523LS (VRC-HIVMAB075-00-AB) 5 mg/kg to be administered SC at weeks 0, 16, 32, 48, and 64.

Group 6

Treatment 6 (T6): VRC07-523LS (VRC-HIVMAB075-00-AB) 2.5 mg/kg to be administered IM at weeks 0, 16, 32, 48 and 64.

OR

Placebo 6 (P6): Sodium Chloride for Injection USP, 0.9% to be administered IM at weeks 0, 16, 32, 48 and 64.

8.2 Study product formulation

VRC07-523LS (Labeled as VRC07-523LS HIV MAb Drug Product VRC-HIVMAB075-00-AB)

VRC07-523LS will be supplied as 10 mL glass vials with a 6.25 ± 0.1 mL fill volume and 3 mL glass vials with a 2.25 mL ± 0.1 mL fill volume, at a concentration of 100 ± 10 mg/mL. Each vial contains a clear, colorless to yellow isotonic, sterile solution essentially free of visible particles; some opaque or translucent particles may be present. The formulation buffer is composed of 50 mM histidine, 50 mM sodium chloride, 5% sucrose and 2.5% sorbitol at pH 6.8.

Vials are intended for single use only and thus do not contain a preservative.

VRC07-523LS product label designates the long-term storage as -35°C to -15°C (-31°F to 5°F). Clinical site storage in a qualified, continuously monitored, temperature-controlled freezer with temperature excursions between -45°C to -10°C (-49°F to 14°F) is acceptable.

Following thaw, VRC07-523LS vials may be stored for up to 24 hours at controlled room temperature (maximum 27°C) and/or up to 2 weeks (14 days) at 2°C to 8°C. Product may not be stored in direct sunlight. If stored at 2°C to 8°C, vials must be equilibrated at controlled room temperature (maximum 27°C) for a minimum of 30 minutes and may be held at room temperature for up to 8 hours prior to product preparation.

8.3 Preparation of study products

Prior to preparation of the first dose (enrollment visit), a new prescription will be sent to the pharmacy. The prescription MUST contain the participant's weight (in kg) based upon the participant's weight (in kg) at the most recent visit where weight was measured (this includes screening) and randomization code (this may NOT be communicated verbally). If this information is NOT on the prescription, the prescription will be returned to the clinic from the pharmacy to be completed appropriately prior to the pharmacist beginning preparation of study product. Subsequent visit weights based upon the participant's weight (in kg) at the most recent visit where weight was measured must be communicated to the pharmacy in writing prior to the day of the visit.

Any changes in weight of more than 10% (between the prior weight and the weight on the day of the study product administration visit) will require an updated visit weight. A new prescription, which includes the new weight, must be written so that product can be prepared based on that weight change.

Pharmacists should keep in mind that the preparation instructions below are considered medium risk per USP 38 General Chapter Physical Tests / <797> Pharmaceutical Compounding - Sterile, and should follow the requirements of

their country, their institution, and their pharmacy regulatory authority regarding these procedures.

8.3.1 VRC07-523LS

VRC07-523LS is a highly concentrated protein solution and may develop white, opaque to translucent particles after thawing. When particles are observed, they may disappear after a few hours at room temperature or storage at 2°C to 8°C.

Ensure that only the required vials are present in the preparation unit during dilution and that medication labels are strictly segregated to avoid mix-ups.

Thawing instructions (all groups):

1. Thaw vial(s) for a minimum of 1 hour at controlled room temperature (maximum 27°C) after removing from the freezer.

   Following thaw, unopened vials of VRC07-523LS may be stored for up to 24 hours at controlled room temperature (maximum 27°C) and/or up to 2 weeks (14 days) at 2°C to 8°C.

2. Keep the material at room temperature during the entire preparation period until use, up to the maximum storage times described in Section 8.2.

3. Prior to preparation for administration, vials should be swirled for 30 seconds with sufficient force to resuspend any visible particles, yet avoiding foaming. DO NOT SHAKE THE VIALS. If the particles are observed, return the vials to 2°C to 8°C storage. If the particles redissolve within the maximum storage times described in Section 8.2 for 2°C to 8°C storage, the vials may be used for product preparation. If particles continue to be observed, do not use the vialed product for SC or IV administration.

   Refrigerated product must be equilibrated at controlled room temperature (maximum 27°C) for a minimum of 30 minutes before preparation and must be used within 8 hours of any subsequent return to room temperature.

4. If the thawed material is not administered within 24 hours of thaw, follow the storage information provided in Section 8.2.

8.3.1.1 Intravenous infusion preparation instructions (T1, T2, and T3):

After thawing, if product was refrigerated again at 2°C to 8°C, vials of VRC07-523LS should be equilibrated to controlled room temperature (maximum 27°C) for a minimum of 30 minutes and may be held at room temperature for up to 8 hours prior to product preparation.

1. Calculate the total milligrams of VRC07-523LS required based on the participant’s weight (in kg) and the randomized treatment group of either 2.5 mg/kg, 5 mg/kg or 20 mg/kg. Remove the total number of vials required from

storage based on a 6 mL or 2 mL withdrawal volume containing 600 mg or 200 mg of VRC07-523LS, respectively.

2. Gently swirl thawed vials for 30 seconds to avoid foaming. DO NOT SHAKE VIALS. Keep the vials upright at all times until ready to withdraw the contents. Do not invert the vial during inspection.

3. Observe vials for particles. If particles are observed, refer to the thawing instructions described above in Section 8.3.1.

4. Using aseptic technique, add the calculated volume of VRC07-523LS (total calculated milligrams of VRC07-523LS) to an appropriately sized IV container (bag/glass bottle) containing 100 mL of Sodium Chloride Injection, USP 0.9% that will also permit the addition of the required calculated volume of VRC07-523LS. Alternatively, if the pharmacist is using an IV container (bag/glass bottle) that cannot accommodate the full VRC07-523LS dose volume plus 100 mL of Sodium Chloride Injection, USP 0.9%, please refer to the Study Products Considerations section of the SSP for further preparation instructions.

5. After product preparation in IV container (bag/glass bottle), the prepared VRC07-523LS may be stored at 2°C to 8°C up to 24 hours or at room temperature (maximum 30°C) for a maximum of 8 hours total including the infusion time. Product may not be stored in direct sunlight. If stored at 2°C to 8°C, prepared product must be equilibrated at room temperature (maximum 30°C) for a minimum of 30 minutes prior to product administration.

6. Label the IV container (bag/glass bottle) as follows:

   - Participant identifier,
   - Participant weight (in kg)
   - Randomized dose of VRC07-523LS of either 2.5 mg/kg, 5 mg/kg, or 20 mg/kg and the total amount (mg) of VRC07-523LS added to the Sodium Chloride Injection, USP 0.9%
   - Final volume of the IV container (bag/glass bottle)
   - Lot number

   The prepared IV label should also be labeled with a DO NOT INFUSE after date and time, as follows:

   - 24 hours if stored at 2°C to 8°C
   - 8 hours, including completion of infusion, if stored at controlled room temperature (not to exceed 27°C)

- Product may not be stored in direct sunlight

Any unused portion of a VRC07-523LS vial will not be used for another participant. Any empty vials, unused portion of entered vials, or unused IV solution which contains study product should be discarded in a biohazard containment bag and incinerated or autoclaved in accordance with institutional or pharmacy policy.

8.3.1.2 Subcutaneous injection preparation instructions (T4 and T5):

1. Calculate the total milligrams of VRC07-523LS required based on the participant's weight (in kg) and the randomized treatment group of either 2.5 mg/kg or 5 mg/kg. Remove the total number of vials required from storage based on a 6 mL of 2 mL withdrawal volume containing 600 mg or 200 mg of VRC07-523LS, respectively.

2. Gently swirl thawed vials for 30 seconds to avoid foaming. DO NOT SHAKE VIALS. Keep the vials upright at all times until ready to withdraw the contents. Do not invert the vial during inspection.

3. Observe vials for particles. If particles are observed, refer to the thawing instructions described above in Section 8.3.1.

4. Using aseptic technique, withdraw the needed volume of VRC07-523LS (total calculated milligrams of VRC07-523LS) from the vial(s) into 1 to 4 syringes using a 5 micron filter needle (see SSP for needle/filter specifications and details). A new filter needle must be used when withdrawing product from vial for each syringe. The filter needle must be discarded prior to dispensing. Cap syringe(s) and affix a needle suitable for SC injection for administration.

5. After product preparation in syringe(s), the prepared VRC07-523LS may be stored at 2°C to 8°C up to 24 hours or at room temperature (maximum 30°C) for a maximum of 4 hours total including the administration time. Product may not be stored in direct sunlight. If stored at 2°C to 8°C, prepared product must be equilibrated at room temperature (maximum 30°C) for a minimum of 30 minutes prior to product administration.

6. Label the syringe (or bag containing multiple syringes) as follows:

    - Participant identifier

    - Participant weight (in kg)

    - Randomized dose of VRC07-523LS of either 2.5 mg/kg or 5 mg/kg and total amount (mg) of VRC07-523LS in syringe(s)

    - Lot number

The prepared syringe/bag labels should also be labeled with a DO NOT administer after time and date, as follows:

- within 4 hours of preparation, including the administration time, when stored at controlled room temperature (maximum 30°C)
- Product may not be stored in direct sunlight

Any unused portion of a VRC07-523LS vial will not be used for another participant. Any empty vials, unused portion of entered vials, or unused IV solution which contains study product should be discarded in a biohazard containment bag and incinerated or autoclaved in accordance with institutional or pharmacy policy.

8.3.1.3 Intramuscular injection preparation instructions (T6 and P6)

1. Calculate the total milligrams of VRC07-523LS required based on the participant's weight and the randomized treatment group of 2.5 mg/kg and the total number of vials required based on a 6 mL or 2 mL withdrawal volume containing 600 mg or 200 mg of VRC07-523LS, respectively.

2. Gently swirl thawed vials for 30 seconds to avoid foaming. DO NOT SHAKE VIALS. Keep the vials upright at all times until ready to withdraw the contents. Do not invert the vial during inspection.

3. Observe vials for particles. If particles are observed, refer to the thawing instructions described above in Section 8.3.1.

4. Using aseptic technique, withdraw the needed volume of VRC07-523LS (total calculated milligrams of VRC07-523LS) from the vial(s) into 1 to 4 syringes using a 5 micron filter needle (see SSP for needle/filter specifications and details). A new filter needle must be used when withdrawing product from vial for each syringe. The filter needle must be discarded prior to dispensing.

   For Placebo, using aseptic technique, draw the appropriate amount of Sodium Chloride for Injection USP, 0.9% into the appropriate number of syringes, based upon the equivalent weight-based dose calculated for the active arm.

5. Cover each syringe (barrel and adapter hub) with an overlay to maintain the blind, due to the fact that VRC07-523LS appearance may be clear, colorless to yellow, which can possibly un-blind clinicians administering the IM placebo saline product which is clear. Cap syringe(s) and affix a needle suitable for IM injection for administration.

6. After product preparation in syringe(s), the prepared VRC07-523LS may be stored at 2°C to 8°C up to 24 hours or at room temperature (maximum 30°C) for a maximum of 4 hours total including the administration time. Product may not be stored in direct sunlight. If stored at 2°C to 8°C, prepared product

must be equilibrated at room temperature (maximum 30°C) for a minimum of 30 minutes prior to product administration.

7. Label the syringe (or bag containing multiple syringes) as follows:

    - Participant identifier
    - Participant weight (in kg)
    - "VRC705-523LS 2.5mg/kg or placebo for IM administration"

    The prepared syringe/bag labels should also be labeled with a DO NOT administer after time and date, as follows:

    - within 4 hours of preparation, including the administration time, when stored at controlled room temperature (maximum 30°C)
    - Product may not be stored in direct sunlight

    Any unused portion of a VRC07-523LS vial will not be used for another participant. Any empty vials, unused portion of entered vials, or unused IV solution which contains study product should be discarded in a biohazard containment bag and incinerated or autoclaved in accordance with institutional or pharmacy policy.

8.4 Administration

8.4.1 VRC07-523LS (Intravenously)

For Groups T1, T2, and T3 - Prior to infusion, if the IV container (bag/glass bottle) containing the prepared solution has been stored at 2°C to 8°C, the prepared IV container (bag/glass bottle) must be equilibrated to room temperature (maximum of 30°C) for 30 minutes or longer and may be held for up to 8 hours, including equilibration time and completion of product administration. (NOTE: This 8 hour period may NOT exceed the DO NOT INFUSE after date and time on the IV bag label.)

The IV container (bag/glass bottle) prepared by the pharmacy will include the weight that was used for preparation of the IV container (bag/glass bottle). The clinician responsible for administration will check the IV container (bag/glass bottle) label and confirm that the participant identifier is correct and that the weight on the IV container (bag/glass bottle) label is within 10% of the participant's current actual weight (refer to Section 8.3 for more information). The entire contents of the prepared investigational study product solution will typically be administered IV over about 15 to 60 minutes using a volumetric pump. The total time needed to administer the dose may be longer based on factors such as participant tolerance. For a 30 minute administration, the rate of

infusion may range from 10-20 mg/kg/hr with the lowest dose group to 80-160 mg/kg/hr with the highest dose group. The mL/hr infusion rate may vary based on the total volume needed to administer a full dose.

An in-line filter infusion set must be used for IV product administration. In-line filters must comply with the specifications described in the HVTN 127/HPTN 087 SSP. When the in-line filter is added to the tubing, prime the administration set with normal saline for injection. CRS staff may prime the administration set with VRC07-523LS only during normal saline shortage. Flush the administration set with about 30 mL or appropriate volume of normal saline at the end of product administration.

8.4.2 VRC07-523LS (Subcutaneously)

For Groups T4 and T5 - Syringes prepared by the pharmacy will contain the total amount (mg) of VRC07-523LS, undiluted, in up to 4 syringes depending on the volume needed to administer the total calculated dose.

If more than one syringe is needed, the zip lock bag containing the syringes will show the total volume to be administered. The clinician responsible for administration will check the syringe/bag label(s) and confirm that the participant identifier is correct and that the weight on the syringe/bag label(s) is within 10% of the participant's current actual weight (refer to Section 8.3 for more information).

The filter needle(s) used during preparation must be discarded prior to dispensing and replaced with a needle suitable for SC injection at the time of administration.

Proper SC technique must be used to ensure administration into the SC fatty layer of the abdomen, inner thigh, or upper arm and a slow push to minimize discomfort or the excessive distention of overlying skin. All SC injections must be at least 2 inches apart.

When preparing a dose in a syringe and administering the dose, consideration should be given to the volume of solution in the needle before and after the dose is administered. Particularly if the needle used to withdraw the product is replaced prior to product administration, consideration should be given to conserving the full dose of product. The pharmacy and clinic staff members are encouraged to work together to administer the dose specified in the protocol.

8.4.3 VRC07-523LS (Intramuscularly)

For Group T6/P6- Syringes prepared by the pharmacy will contain the total amount (mg) of VRC07-523LS undiluted or placebo, in up to 4 syringes depending on the volume needed to administer the total calculated dose.

If more than one syringe is needed, the zip lock bag containing the syringes will show the total volume to be administered. The clinician responsible for administration will check the syringe/bag label(s) and confirm that the participant

identifier is correct and that the weight on the syringe/bag label(s) is within 10% of the participant's current actual weight (refer to Section 8.3 for more information).

The filter needle(s) used during preparation must be discarded prior to dispensing and replaced with a needle suitable for IM injection at the time of administration.

Proper IM technique shall be used to ensure administration into gluteus or deltoid. All IM injections must be at least 2 inches apart.

When preparing a dose in a syringe and administering the dose, consideration should be given to the volume of solution in the needle before and after the dose is administered. Particularly if the needle used to withdraw the product is replaced prior to product administration, consideration should be given to conserving the full dose of product. The pharmacy and clinic staff members are encouraged to work together to administer the dose specified in the protocol.

8.5 Acquisition of study products

VRC-HIVMAB075-00-AB (VRC07-523LS) is provided by the VRC/NIAID.

Filter needles, in-line filter infusion sets and Sodium Chloride for Injection USP, 0.9% should be obtained by the CRS. Please refer to the study product considerations section of the SSPs for product specific reference numbers.

Once a CRS is protocol registered, the pharmacist can obtain study products from the NIAID Clinical Research Products Management Center (CRPMC) by following the ordering procedures given in Pharmacy Guidelines and Instructions for DAIDS Clinical Trials Networks.

8.6 Pharmacy records

The CRS pharmacist is required to maintain complete records of all study products. The pharmacist of record is responsible for maintaining randomization codes and randomization confirmation notices for each participant in a secure manner.

8.7 Final disposition of study products

All unused study products must be returned to the CRPMC after the study is completed or terminated unless otherwise instructed by the CRPMC. The procedures and relevant form are included in the Pharmacy Guidelines and Instructions for DAIDS Clinical Trials Networks.

9 Clinical procedures

The schedules of clinical procedures are shown in Appendix G.

9.1 Informed consent

Informed consent is the process of working with participants so that they fully understand what will and may happen to them while participating in a research study. The informed consent form documents that a participant (1) has been informed about the potential risks, benefits, and alternatives to participation, and (2) is willing to participate in the study. Informed consent encompasses all written or verbal study information CRS staff provide to the participant, before and during the trial. CRS staff will obtain informed consent of participants according to HVTN and HPTN policies and procedures.

The informed consent process continues throughout the study. Key study concepts should be reviewed periodically with the participant and the review should be documented. At each study visit, CRS staff should consider reviewing the procedures and requirements for that visit and for the remaining visits. Additionally, if any new information is learned that might affect the participants' decisions to stay in the trial, this information will be shared with trial participants. If necessary, participants will be asked to sign revised informed consent forms.

A CRS may employ recruitment efforts prior to the participant consenting. For example, some CRSs use a telephone script to prescreen people before they come to the clinic for a full screening visit. Participants must sign a screening or protocol-specific consent before any procedures are performed to determine eligibility. CRSs must submit recruitment and prescreening materials to their IRB/EC and any applicable RE for human subjects protection review and approval.

Note: As defined in the DAIDS Protocol Registration Manual, an RE is "Any group other than the local IRB/EC responsible for reviewing and/or approving a clinical research protocol and site-specific ICFs [informed consent forms] prior to implementation at a site." CRSs are responsible for knowing the requirements of their applicable REs.

9.1.1 Screening consent form

Without a general screening consent, screening for a specific study cannot take place until the site receives protocol registration from the DAIDS RSC Protocol Registration Office.

Some CRSs have approval from their IRB/EC and any applicable RE to use a general screening consent form that allows screening for an unspecified HIV prevention clinical trial. In this way, CRS staff can continually screen potential participants and, when needed, proceed quickly to obtain protocol-specific

enrollment consent. Sites conducting general screening or prescreening approved by their IRB/EC and any applicable RE may use the results from this screening to determine eligibility for this protocol, provided the tests are conducted within the time periods specified in the eligibility criteria.

9.1.2 Protocol-specific consent forms

The protocol-specific consent forms describe the study products to be used and all aspects of protocol participation, including screening and enrollment procedures. A sample protocol-specific consent form for the main study is located in Appendix A. A separate sample consent form for other uses of specimens is located in Appendix C.

Each CRS is responsible for developing a protocol-specific consent form(s) for local use, based on the sample protocol-specific consent forms in Appendix A and Appendix C. The consent form(s) must be developed in accordance with requirements of the following:

- CRS’s IRB/EC and any applicable REs,
- CRS’s institution, and
- Elements of informed consent as described in Title 45, CFR Part 46 and Title 21 CFR, Part 50, and in the International Conference on Harmonisation (ICH) E6, Good Clinical Practice: Consolidated Guidance 4.8.

Study sites are strongly encouraged to have their local CABs review their sites-specific consent forms. This review should include, but should not be limited to, issues of cultural competence, local language considerations, and the level of understandability.

The sample informed consent forms include instructions throughout the documents for developing specific content.

Regarding protocol registration, sites should follow procedures outlined in the current version of the DAIDS Protocol Registration Manual.

9.1.3 Assessment of Understanding

Study staff are responsible for ensuring that participants fully understand the study before enrolling them. This process involves reviewing the informed consent form with the participant, allowing time for the participant to reflect on the procedures and issues presented, and answering all questions completely.

An Assessment of Understanding is used to document the participant’s understanding of key concepts in this clinical trial. The participant must complete the Assessment of Understanding before enrollment. Staff may provide assistance in reading and understanding the questions and responses, if necessary. Participants must verbalize understanding of all questions answered incorrectly.

This process and the participant's understanding of the key concepts should be recorded in source documentation at the site.

IRB/EC and any applicable RE may require that a participant has signed either a screening or protocol-specific consent document prior to administering the Assessment of Understanding. The consent process (including the use of the Assessment of Understanding) should be explained thoroughly to the IRB/EC and any applicable RE, whose recommendations should be followed.

9.2 Pre-enrollment procedures

Screening may occur over the course of several contacts/visits, up to and including before study product administration on day 0. All inclusion and exclusion criteria must be assessed within 56 days before enrollment, unless otherwise specified in the eligibility criteria (or below in this section).

After the appropriate informed consent has been obtained and before enrollment, the following procedures are performed:

- Medical history, documented in the case history record
- Assessment of whether the volunteer is at low risk for HIV infection (see Appendix I)
- Complete physical examination, including height, weight, vital signs, and clinical assessments of head, ears, eyes, nose, and throat; neck; lymph nodes; heart; chest; abdomen; extremities; neurological function; and skin
- Assessment of concomitant medications the volunteer is taking, including prescription and nonprescription drugs, vitamins, topical products, alternative/complementary medicines (eg, herbal and health food supplements), recreational drugs, vaccinations, and allergy shots
- Laboratory tests as defined in the inclusion and exclusion criteria, including:
  - Screening HIV,
  - HBsAg,
  - Anti-HCV Abs,
  - Syphilis,
  - CBC with differential and platelets,
  - Chemistry panel (ALT, creatinine),
  - Urine dipstick (urinalysis if indicated; see Section 9.7),
  - Urine or serum pregnancy test (volunteers who were assigned female sex at birth); Persons who are not of reproductive potential due to having

undergone total hysterectomy with bilateral oophorectomy (verified by medical records), are not required to undergo pregnancy testing;

- Administration of behavioral risk assessment questionnaire

- Obtaining of volunteer demographics in compliance with the NIH Policy on Reporting Race and Ethnicity Data: Subjects in Clinical Research, Aug. 8, 2001 (available at http://grants.nih.gov/grants/guide/notice-files/NOT-OD-01-053.html)

- Counseling on HIV testing and risk reduction, performed in compliance with the US Centers for Disease Control and Prevention (CDC)'s current guidelines or other local guidelines for HIV counseling, testing, and referral as described in Section 9.5

- Discussion of pregnancy prevention. A pregnant or breastfeeding person may not be enrolled in this trial. Specific criteria and assessment of contraception and pregnancy status are described in study inclusion criteria. Discussion of pregnancy prevention includes advising a participant who was assigned female sex at birth and who reports no current sexual activity that could lead to that participant becoming pregnant to have a plan to begin adequate birth control. This plan would be put to use if, during the study, the participant becomes sexually active in a way that could lead to that participant becoming pregnant.

9.2.1 Use of screening results from another HVTN or HPTN study

If a participant screens for an HVTN or HPTN study at the same CRS but then does not join that study, screening results from that effort may be applied to the screening for this protocol, as long as the screening was done under participant consent, the participant has signed a consent form to begin screening for this study, and the tests were conducted within the time periods specified in the eligibility criteria (see Sections 7.1 and 7.2).

9.3 Enrollment and study product administration visits

Enrollment is simultaneous with first study product administration. The CRS requests the randomization assignment via a Web-based randomization system. In general, the interval between randomization and enrollment should not exceed 4 working days. However, circumstances may require a participant's enrollment visit to be changed. This may exceed the 4-day randomization time limit.

At all infusion/injection visits, the following procedures are performed before study product administration:

- Abbreviated physical examination, including weight, vital signs, and a symptom-directed evaluation by history and/or appropriate physical exam based on participant self-reported symptoms or complaints;
- Assessment of baseline Solicited AEs;
- Assessment of concomitant medications (as described in Section 9.2);
- Assessment of any new or unresolved Unsolicited AEs/intercurrent illnesses;
- CBC with differential and platelets,
- Chemistry panel (see Section 9.2), and
- Urine or serum pregnancy test (for participants who were assigned female sex at birth). Persons who are NOT of reproductive potential due to having undergone total hysterectomy or bilateral oophorectomy (verified by medical records), are not required to undergo pregnancy testing.

Following completion of all procedures in the preceding list, and if results indicate that study product administration may proceed, study product is prepared and administered (see Sections 8.3 and 8.4).

Administration of all infusions and injections during a study product administration visit must be accomplished within 1 calendar day.

Immediately following infusion/injection, the participant remains in the clinic for observation. See the HVTN 127/HPTN 087 SSP for details regarding infusion visit protocols and subsequent infusion observation and Solicited AE assessment procedures that CRSs must follow. The CRS will make arrangements to be in contact with the participant during the Solicited AE period (as described in Section 9.8 and the SSP).

The following procedures will be performed at all infusion/injection visits. These procedures may be performed prior to or following study product administration:

- Risk reduction counseling (as described in Section 9.5);
- For participants capable of becoming pregnant, pregnancy prevention assessment (as described in Section 9.2 and 9.6); and

The following procedure will be performed at all infusion/injection visits following study product administration:

- Acceptability questionnaire.

Additional procedures will be performed at scheduled visits as specified in Appendix G:

- HIV infection assessment including pretest counseling and HIV diagnostic testing. A subsequent follow-up contact is conducted to provide post-test counseling and to report results to participant;
- Behavioral risk assessment questionnaire;
- Assessment of new or unresolved social impacts (site staff will ask participant about the status of any unresolved social impacts and if s/he has experienced any new social impacts as a result of the trial participation).
- Social impact questionnaire;
- Confirm that participants received HIV test results from previous visit. If not, provide test results and post-test counseling as appropriate; and
- Specimen collection (should be completed prior to study product administration).

9.4 Follow-up visits

Procedures will be performed at scheduled follow-up visits as specified in Appendix G:

- Assessment of new or unresolved social impacts (site staff will ask participant about the status of any unresolved social impacts and if s/he has experienced any new social impacts as a result of the trial participation);
- Assessment of new or continuing concomitant medications (as described in Section 9.2); and
- Assessment of new or unresolved AEs/intercurrent illnesses or AESIs (as described in Section 11.2.2 and Appendix H);
- Risk reduction counseling (as described in Section 9.5);
- Pregnancy prevention assessment (as described in Sections 9.2 and 9.6); and
- Behavioral risk assessment questionnaire;
- Administration of the social impact assessment questionnaire (types of impacts assessed involve personal relationships, health insurance, life insurance, educational or employment opportunities, housing, immigration, or travel);
- Ask participants in Group 6 whether they believe they received the active study product or the control;

- HIV infection assessment including pre-test counseling and HIV diagnostic testing. A subsequent follow-up contact is conducted to provide post-test counseling and to report results to participant;

- Confirm that participants received HIV test results from previous visit. If not, provide test results and post-test counseling as appropriate;

- Abbreviated physical examination including weight, vital signs, and a symptom-directed evaluation by history and/or appropriate physical exam based on participant self-reported symptoms or complaints;

- Complete physical examination, including weight, vital signs, and clinical assessments of head, ears, eyes, nose, and throat; neck; lymph nodes; heart; chest; abdomen; extremities; neurological function; and skin;

- Specimen collection;

- Clinical laboratory tests including:
  - CBC with differential,
  - Chemistry panel (see Section 9.2), and
  - Urine dipstick (urinalysis if appropriate; see Section 9.7); and

- Urine or serum pregnancy test (for participants who were assigned female sex at birth). Persons who are NOT of reproductive potential due to having undergone total hysterectomy or bilateral oophorectomy (verified by medical records), are not required to undergo pregnancy testing.

9.5 HIV counseling and testing

HIV counseling will be performed in compliance with the CDC's guidelines or other local guidelines for HIV counseling and referral. HIV testing will be performed in accordance with the protocol-specific HIV testing algorithm following enrollment.

Participants will be counseled routinely during the trial on the avoidance of HIV infection.

Potential participants identified as being HIV infected during screening are not enrolled. All participants who become HIV infected during the study will be terminated from this study. Potential and enrolled participants identified as HIV infected will be referred for medical treatment, counseling, and management of the HIV infection. These individuals may also be referred to appropriate ongoing clinical trials or observational studies.

9.5.1 Study product-related seroreactivity

Tests of human sera containing monoclonal antibodies similar to VRC07-523LS have been tested using a variety of commercially available HIV test kits without any indication of reactivity. For this reason, we do not anticipate that receipt of the study product will cause a reactive result on currently available HIV test kits. However, this remains a theoretical possibility.

Because this possibility cannot be categorically eliminated, study staff will advise study participants to confine their HIV testing while in the study to that provided through the CRS. Staff will also inform study participants of the likelihood of routine HIV testing being offered or performed outside the study CRS at emergency rooms, clinics, and medical offices, and will inform participants of their right to opt out of HIV testing outside the study site. CRS staff should inform study participants if local and/or state/regional policies and regulations permit medical providers to perform HIV testing without first informing patients. If this is the case, then CRS staff should advise study participants that they may decline testing preemptively. CRS staff should also inform participants if positive results must be reported to local public health authorities. CRS staff should provide participants with CRS contact information and should encourage participants to ask medical providers to contact the CRS. The CRS can verify that the participant is in an HIV mAb clinical trial and should only be tested at the study CRS.

9.6 Contraception status

Contraception status is assessed and documented at screening and study product administration visits for a participant who was assigned female sex at birth and who is sexually active in a way that could cause that participant to become pregnant. Prior to enrollment and throughout the study, staff will ask participants to verbally confirm their use of adequate contraceptive methods. A participant who was assigned female sex at birth and is sexually active in a way that could cause that participant to become pregnant should be reminded at all scheduled clinic visits of the importance of using contraception and should be referred to specific counseling, information, and advice as needed. (Specific contraception requirements are listed in Section 7.1). This reminder should be documented in the participant's study record.

Self-reported infertility—including having undergone hysterectomy, bilateral oophorectomy, or tubal ligation—must be documented in the participant's study record.

9.7 Urinalysis

Urine dipstick testing may be performed in the clinic or the lab. The following analytes should be analyzed and recorded: specific gravity, protein, blood/hemoglobin, pH, urobilinogen, bilirubin, ketones, leucocyte esterase,

nitrite, and glucose. The examination is performed on urine obtained by clean catch. Urine microscopy is required when the protein result is 1+, 2+, 3+, or 4+ (or semi-quantitative 30, 100, 300, or 2000 g/L) and/or the blood/hemoglobin result is trace, 1+, 2 +, 3+, or 4+, but not required (unless otherwise clinically indicated) if the dipstick result is abnormal due to non-urinary bleeding.

If the dipstick performed for screening is transiently abnormal with an exclusionary result (eg, 2+ proteinuria), then repeat the dipstick and, if within the eligibility limits specified in the protocol, the participant may be enrolled (see SSP for further detail).

A follow-up urine test should be deferred if a participant is experiencing non-urinary bleeding (eg, menstruation), but should be performed as soon as possible. If a follow-up dipstick is abnormal due to non-urinary bleeding (eg, a participant’s menstrual period), document in the comment section of the CRF and repeat the dipstick once the participant is no longer experiencing non-urinary bleeding. In this case, a urine microscopy is not required (see SSP for further detail). If a follow-up dipstick is abnormal due to infection, document this issue in the participant’s source documentation and provide appropriate treatment and/or referral.

9.8 Assessments of Solicited AEs

For all participants, baseline assessments are performed before each infusion or injection and assessments for Solicited AEs are performed after each infusion or injection. All Solicited AEs are graded according to the Division of AIDS Table for Grading the Severity of Adult and Pediatric Adverse Events, Corrected Version 2.1 dated July 2017, except as noted in Section 11.2.2.

The Solicited AE assessment period is 3 full days following each infusion or injection per the assessment schedule shown in Table 9-1. Participants are instructed to record symptoms using a memory tool. The site staff and the participant will be in contact after the 3-day Solicited AE assessment period, or sooner if indicated. See SSP for further detail. In general, a participant who self-reports any reaction following infusion/injection that is greater than mild is seen by a clinician within 48 hours after onset, unless the reaction is improving and/or has completely resolved. Clinic staff will follow new or unresolved Solicited AEs present at day 3 to resolution.

Solicited AEs are reported using CRFs that correspond to the time of assessment in Table 9-1. Solicited AE assessments include assessments of systemic and local symptoms, and study product-related lesions. Events not listed on a CRF, or with an onset after the Solicited AE assessment period (day of infusion/injection and 3 full days after), or those SAEs/Unsolicited AEs requiring expedited reporting to DAIDS, are recorded on an AE Log.

Table 9-1 Schedule of Solicited AE assessments

Day	Time	Performed by
0[a]	Baseline: before infusion or injection	CRS clinician
	Early: 25-60 minutes after infusion or injection	CRS clinician
	Between early assessment and 11:59pm day 0	CRS clinician or participant
1-3[b]	Between 12:00am and 11:59pm on the respective day	CRS clinician or participant

[a] Day of infusion or injection

[b] New or unresolved Solicited AEs present on day 3 are followed until resolution

9.8.1 Assessment of systemic and local symptoms

Systemic symptoms to be assessed as solicited AEs in this trial include increased body temperature, chills, malaise and/or fatigue, headache, myalgia, arthralgia, nausea, urticaria, non-exertional dyspnea, non-exertional tachycardia (assessed by CRS staff, not by the participant), generalized pruritus, facial flushing, and unexplained diaphoresis. Local symptoms include pain or tenderness at the infusion/injection site. Additionally, in participants receiving SC injection local pruritus will be assessed. The daily maximum severity reached for each symptom during the assessment period is reported (see SSP).

Body temperature is measured by oral or infrared thermometry. All temperatures must be measured by non-axillary thermometry. This includes temperatures taken in the clinic, as well as temperatures taken by participants during the solicited AE assessment period. Temperature is reported in degrees Celsius. If temperature is measured in Fahrenheit, the conversion to Celsius should be documented in the participant’s chart note. A measurement is taken once daily during the assessment period and should be repeated if participant is feeling feverish.

9.8.2 Assessment of infusion/injection site

Typical infusion and injection site reactions are erythema/redness and induration/swelling. The maximum horizontal and maximum vertical measurements for all infusion/injection site reactions are recorded.

All infusion/injection site reactions are monitored until resolution. Areas greater than 25 cm^2 are followed daily; otherwise, the frequency of follow-up is based on clinician judgment.

9.9 Visit windows and missed visits

Visit windows are defined in SSP. For a visit not performed within the window period, a Missed Visit form is completed.

If a participant misses a scheduled visit, CRS staff should attempt to bring the participant in as soon as possible to complete the required safety assessments and other procedures. See the SSP for more details. If a participant missed an

infusion/injection visit or if study product administration must be permanently discontinued, see Section 7.3.2 and Section 7.3.3 for resolution.

9.10 Early termination visit

In the event of early participant termination, site staff should consider if the following assessments are appropriate: a final physical examination, clinical laboratory tests (including urine dipstick, CBC with differential, ALT, and creatinine), pregnancy testing, social impact assessment, and HIV test.

9.11 Pregnancy

If a participant becomes pregnant during the course of the study, no more infusions or injections of study product will be given, but remaining visits and study procedures should be completed unless medically contraindicated or applicable regulations require termination from the study. If the participant terminates from the study prior to the pregnancy outcome, the site should make every effort to keep in touch with the participant in order to ascertain the pregnancy outcome. Pregnancies and pregnancy outcomes will be reported.

10 Laboratory

10.1 CRS laboratory procedures

The HVTN 127/HPTN 087 Site Lab Instructions and SSP provide further guidelines for operational issues concerning the clinical and processing laboratories. These documents include guidelines for general specimen collection, special considerations for phlebotomy, specimen labeling, whole blood processing, HIV screening/diagnostic testing, and general screening and safety testing.

Tube types for blood collection are specified in Appendix F. For tests performed locally, the local lab may assign appropriate tube types.

In specific situations, the blood collection tubes may be redirected to another laboratory or may require study-specific processing techniques. In these cases, laboratory special instructions will be posted on the protocol-specific section of the HVTN website.

Of note, all assays described below are performed as research assays and are not approved for use in medical care. Results from these assays are not made available to participants or medical professionals to guide treatment decisions.

10.2 Total blood volume

Required blood volumes per visit are shown in Appendix F. Not shown is any additional blood volume that would be required if a safety lab needs to be repeated, or if a serum pregnancy test needs to be performed. The additional blood volume would likely be minimal. The total blood volume drawn for each participant will not exceed 500 mL in any 56-day (8-week) period.

10.3 VRC07-523LS concentrations

VRC07-523LS concentrations will be measured in serum. The binding assay may employ the VRC01 Fab specific 5C9 mAb or another suitable protein. 5C9 is an anti-idiotype antibody cloned from a single B cell that was sorted by flow cytometry using a VRC01 scFv probe. 5C9 also captures VRC07-523LS given the homology of the proteins. Serum concentrations of VRC07-523LS will be based upon the concentrations of sample dilutions falling within the linear range of the standard curve.

10.4 Endpoint assays: humoral

10.4.1 Neutralizing antibody assay

HIV-1–specific neutralizing antibody (nAb) assays will be performed on serum samples from study participants taken at postadministration timepoints and at baseline. The TZM-bl assay (77, 78) will test neutralization of a panel of Tier 2 viruses that exhibit a range of known sensitivities to VRC07-523LS. The viruses will be selected from a global panel and/or clade-specific panels (79, 80).

10.4.2 ADA detection assay

Anti-VRC07-523LS antibody assays will be performed on select serum samples from study participants collected at baseline and after antibody administration. ADA will be measured by using the Meso Scale Discovery (MSD) platform with VRC07-523LS as the target antigen.

10.4.3 ADA functional assay

A functional ADA assay will be used to confirm any positive activity that is observed in the ADA detection assay (MSD). Functional activity will measure a reduction in VRC07-523LS neutralizing activity against a qualified virus in the TZM-bl assay.

10.4.4 Binding antibody multiplex assay (BAMA)

HIV-1–specific total binding IgG may be assessed on serum samples from study participants collected at baseline and after antibody administration with reagents that have known reactivity with VRC07-523LS.

10.5 Exploratory studies

Samples may be used for other testing and research related to furthering the understanding of HIV immunology, antibody mediated prevention, or vaccines. In addition, cryopreserved samples may be used to perform additional assays to support standardization and validation of existing or newly developed methods.

10.6 Specimen storage and other use of specimens

The Networks store specimens from all study participants indefinitely, unless a participant requests that specimens be destroyed or if required by IRB/EC, or RE.

Other use of specimens is defined as studies not covered by the protocol or the informed consent form for the main study (see Appendix A).

This research may relate to HIV, vaccines, monoclonal antibodies, the immune system, and other diseases. This could include genetic testing and, potentially,

genome-wide studies. This research is done only to the extent authorized in each study site's informed consent form, or as otherwise authorized under applicable law. Other research on specimens will occur only after review and approval by the HVTN, the HPTN, the IRB/EC of the researcher requesting the specimens, and the CRS's IRBs/ECs/REs if required.

As part of consenting for the study, participants document their initial decision to allow or not allow their specimens to be used in other research, and they may change their decision at any time. The participant's initial decision about other use of their specimens, and any later change to that decision, is recorded by their CRS in a Web-based tool that documents their current decisions for other use of their specimens. The Networks will only allow other research to be done on specimens from participants who allow such use.

CRSs must notify HVTN Regulatory Affairs if institutional or local governmental requirements pose a conflict with or impose restrictions on specimen storage or other use of specimens.

10.7 Biohazard containment

As the transmission of HIV and other blood-borne pathogens can occur through contact with contaminated needles, blood, and blood products, appropriate precautions will be employed by all personnel in the drawing of blood and shipping and handling of all specimens for this study, as currently recommended by the CDC and the NIH or other applicable agencies.

All dangerous goods materials, including Biological Substances, Category A or Category B, must be transported according to instructions detailed in the International Air Transport Association Dangerous Goods Regulations.

11 Safety monitoring and safety review

11.1 Safety monitoring and oversight

11.1.1 HVTN 127/HPTN 087 PSRT

The HVTN 127/HPTN 087 PSRT is composed of the following members:

- DAIDS medical officer representatives
- Protocol chairs
- Protocol Team leader
- Core medical monitor
- Clinical safety specialist

The clinician members of HVTN 127/HPTN 087 PSRT are responsible for decisions related to participant safety.

The Protocol Team clinic coordinator, clinical data manager, study product developer representative, clinical research manager, clinical trials manager, and others may also be included in HVTN 127/HPTN 087 PSRT meetings.

11.1.2 HVTN SMB

The SMB is a multidisciplinary group consisting of biostatisticians, clinicians, and experts in HIV vaccine and drug research that, collectively, has experience in the conduct and monitoring of vaccine and drug trials. Members of the SMB are not directly affiliated with the protocols under review.

The SMB reviews safety data, unblinded as to treatment arm, approximately every 4 months. The reviews consist of evaluation of cumulative Solicited AEs, Unsolicited AEs, laboratory safety data, and individual reports of AEs requiring expedited reporting to DAIDS. The SMB conducts additional special reviews at the request of the HVTN 127/HPTN 087 PSRT.

Study sites will receive SMB summary minutes and are responsible for forwarding them to their IRB/EC and any applicable RE.

11.1.3 Roles and responsibilities in safety monitoring

The roles and responsibilities of the SDMC in relation to safety monitoring include:

- Maintaining a central database management system for clinical data;

- Providing reports of clinical data to appropriate groups such as the HVTN 127/HPTN 087 PSRT and HVTN SMB (see Section 11.1.2);

The roles and responsibilities of the HVTN CSS or HVTN Core designee in relation to safety monitoring include:

- Daily monitoring of clinical data for events that meet the safety pause and HVTN 127/HPTN 087 PSRT AE review criteria (see Section 11.3);
- Notifying CRSs and other groups when safety pauses or planned holds are instituted and lifted (see Section 11.3);
- Querying CRSs for additional information regarding reported clinical data; and
- Providing support to the HVTN 127/HPTN 087 PSRT.

11.2 Safety reporting

11.2.1 Submission of safety forms to SDMC

Site staff must submit all safety forms (eg, Solicited AEs, Unsolicited AEs, urinalysis, local lab results, and concomitant medications) before the end of the next business day, excluding federal or bank holidays. The forms should not be held in anticipation of additional information at a later date. If additional information is received at a later date, the forms should be updated and resubmitted before the end of the next business day after receiving the new information. For the case of a longer site holiday closure, site staff must submit the data by the end of the 5th day (local time) after receiving the information even if this day is a holiday.

For example: If the site becomes aware of an AE on Thursday (Day 0), the site must submit the data by the end of the next business day, on Friday. If there is a longer site holiday closure, then this AE may be reported no later than the end of the fifth day, Monday (Day 4). If Monday is a holiday as well, all safety forms still need to be submitted by the end of Monday (Day 4).

11.2.2 AE reporting

An AE is any untoward medical occurrence in a clinical investigation participant administered a study product/procedure(s) and which does not necessarily have a causal relationship with this treatment. An AE can therefore be any unfavorable and unintended sign (including an abnormal laboratory finding), symptom, or disease temporally associated with the use of an investigational study product/procedure(s), whether or not related to the investigational study product/procedure(s).

The study Unsolicited AE reporting period is from study enrollment of a trial participant to (and including) the Week 80 visit (v16.0)

Only AESI are reported to the SDMC after the completion of that visit (bulleted above) to the end of trial participation for each participant (see Appendix H).

All AEs are graded according to the Division of AIDS (DAIDS) Table for Grading the Severity of Adult and Pediatric Adverse Events, Corrected Version 2.1, July 2017, available on the RSC website at http://rsc.tech-res.com/clinical-research-sites/safety-reporting/daids-grading-tables, except:

- Unintentional weight loss is required to be reported as an AE only if it is considered to be potentially deleterious to the participant's health (see HVTN 127/HPTN 087 SSP)

- Infusion Site Erythema or Redness and Infusion Site Induration or Swelling will not consider interference with usual social and functional activities such that:
    - Grade 1 is: 2.5 to < 5 cm in diameter OR 6.25 to < 25 cm^2 surface area;
    - Grade 2 is: ≥ 5 to < 10 cm in diameter OR ≥ 25 to < 100 cm^2 surface area;
    - Grade 3 is: ≥ 10 cm in diameter OR ≥ 100 cm^2 surface area OR Ulceration OR Secondary infection OR Phlebitis OR Sterile abscess OR Drainage;
    - Grade 4 is: Potentially life-threatening consequences (eg, abscess, exfoliative dermatitis, necrosis involving dermis or deeper tissue);

- Injection Site Erythema or Redness and Injection Site Induration or Swelling will not consider interference with usual social and functional activities such that:
    - Grade 1 is: 2.5 to < 5 cm in diameter OR 6.25 to < 25 cm^2 surface area;
    - Grade 2 is: ≥ 5 to < 10 cm in diameter OR ≥ 25 to < 100 cm^2 surface area;
    - Grade 3 is: ≥ 10 cm in diameter OR ≥ 100 cm^2 surface area OR Ulceration OR Secondary infection OR Phlebitis OR Sterile abscess OR Drainage;
    - Grade 4 is: Potentially life-threatening consequences (eg, abscess, exfoliative dermatitis, necrosis involving dermis or deeper tissue);

- Infusion reactions (see HVTN 127/HPTN 087 SSP)

All AEs are reported to the SDMC on the appropriate CRF. Clinic staff should evaluate every AE to determine if (1) the AE meets the requirements for expedited reporting to DAIDS (see Section 11.2.3), (2) if the AE meets the criteria for a safety pause/prompt AE review (see Section 11.3), and (3) if the AE is listed as an AESI. A list of AESI to be reported in this protocol is provided in Appendix H.

Sites are expected to notify HVTN clinical safety staff of any serious safety concern requiring their attention (Table 11-1). Telephone numbers and email addresses are found on the protocol home page on the HVTN 127/HPTN 087 protocol-specific site (https://members.hvtn.org/protocols/hvtn127-hptn087). Concerns requiring immediate attention should be communicated by calling the clinical safety phone.

In the case of email notification, clinical safety staff will reply during working hours (HVTN Core local time) to confirm that the email has been received and reviewed. If email service is not available, the CRS should notify clinical safety staff of the event by telephone, then submit CRFs.

In addition, site investigators are required to submit AE information in accordance with IRB/EC and any applicable RE requirements.

11.2.3 Expedited reporting of AEs to DAIDS

Requirements, definitions and methods for expedited reporting of AEs are outlined in Version 2.0 (January 2010) of the *Manual for Expedited Reporting of Adverse Events* to DAIDS (DAIDS EAE Manual), which is available on the RSC website at http://rsc.tech-res.com/clinical-research-sites/safety-reporting/manual. The SAE Reporting Category will be used for this study.

The internet-based DAIDS Adverse Experience Reporting System (DAERS) must be used for expedited AE (EAE) reporting to DAIDS. In the event of system outages or technical difficulties, expedited AE reports may be submitted via the DAIDS EAE Form. This form is available on the DAIDS RSC website at http://rsc.tech-res.com/clinical-research-sites/safety-reporting/daids/paper-eae-reporting.

For questions about DAERS, please contact CRMSsupport@niaid.nih.gov or from within the DAERS application itself.

For questions about EAE reporting, please contact the DAIDS RSC Safety Office at (DAIDSRSCSafetyOffice@tech-res.com).

The study products for which expedited reporting are required are:

- VRC-HIVMAB075-00-AB (VRC07-523LS)

While the participant is in the study, from enrollment to the end of trial participation for that participant, the SAE Reporting Category will be used.

After the end of trial participation for that participant, unless otherwise noted, only Suspected, Unexpected Serious Adverse Reactions (SUSARs) as defined in Version 2.0 of the DAIDS EAE Manual must be reported to DAIDS, if the study staff become aware of the events. All AESI will be considered unexpected, and, if deemed related to the study products, will be reported as SUSARs, if applicable.

The NIAID/DAIDS will report all unexpected SAEs related to the study products observed in this clinical trial to the FDA in accordance with 21 CFR 312.32 (IND Safety Reports). However, because safety is a primary study endpoint, the Sponsor Medical Officer will not routinely be unblinded to study treatment assignment when there is an assessment of relatedness of the SAE with the study product(s); and the safety report will be sent to the FDA based on the blinded attribution assessment. In addition, the NIAID/DAIDS or designee(s) will prepare and file expedited reports to other appropriate regulatory authorities within the timelines required by pertinent national regulatory agencies.

If the PSRT believes unblinding is appropriate (eg, if unblinding to treatment assignment will assist with the clinical management of the SAE), the PSRT will consult the independent HVTN SMB for a recommendation. In the event the HVTN SMB determines that unblinding is indicated, the SMB will inform the individual who requested unblinding (eg, site physician) of the participant’s treatment assignment in such a manner as to maintain the study blind of the PSRT and study team. For additional impact and management of SAEs on the study, see Section 11.3.

CRS Investigators of Record (IoRs)/designees will submit AE information and any other relevant safety information to their IRBs/ECs in accordance with IRB/EC requirements.

11.3 Safety pause and prompt PSRT AE review

When a trial is placed on safety pause, all enrollment and study product administration related to the event that triggered the pause will be held until further notice. The AEs that will lead to a safety pause or prompt HVTN 127/HPTN 087 PSRT AE review are summarized in Table 11-1. Study product administration may be suspended for safety concerns other than those described in the table, or before pause rules are met, if, in the judgment of the HVTN 127/HPTN 087 PSRT, participant safety may be threatened. Criteria for an individual participant’s departure from the schedule of study product administration are listed in Section 7.3.

Table 11-1 AE notification and safety pause/AE review rules

Event and relationship to study products	Severity	CRS action[a]	HVTN Core action[b]
SAE, related	Grade 5 or Grade 4	Phone immediately, email and submit forms immediately	Immediate pause
SAE, not related	Grade 5	Phone immediately, email and submit forms immediately	Immediate PSRT notification
SAE, related	Grade 3	Email and submit forms immediately	Immediate PSRT notification and prompt PSRT AE review to consider pause
AE[c], related	Grade 4 or 3	Email and submit forms immediately	Immediate PSRT notification and prompt PSRT AE review to consider pause

[a] Phone numbers and email addresses are found on the Protocol home page on the HVTN Members' site (https://members.hvtn.org/protocols/hvtn127-hptn087).

[b] HVTN CSS or HVTN Core designee

[c] Does not include subjective Solicited AEs (injection or infusion site pain/tenderness, fatigue/malaise, headache, non-exertional dyspnea, generalized pruritus, facial flushing, unexplained diaphoresis).

For all safety pauses, HVTN Core notifies the HVTN 127/HPTN 087 PSRT, HVTN Regulatory Affairs, DAIDS Pharmaceutical Affairs Branch (PAB), DAIDS Regulatory Affairs Branch (RAB), DAIDS Safety and Pharmacovigilance Team (SPT), and participating CRSs. When an immediate safety pause is triggered, HVTN Core notifies the SMB.

Once a trial is paused, the HVTN 127/HPTN 087 PSRT reviews safety data and decides whether the pause can be lifted or permanent discontinuation of study product administration is appropriate, consulting the SMB if necessary. HVTN Core notifies the participating CRSs, HVTN Regulatory Affairs, DAIDS PAB, DAIDS RAB, and DAIDS SPT of the decision regarding resumption or discontinuation of study product administration. Based on the HVTN 127/HPTN 087 PSRT assessment, DAIDS RAB notifies the FDA as needed.

If an immediate HVTN 127/HPTN 087 PSRT notification or prompt HVTN 127/HPTN 087 PSRT AE review is triggered, HVTN Core notifies the HVTN 127/HPTN 087 PSRT as soon as possible during working hours (HVTN Core local time)—or, if the information was received during off hours, by the morning of the next workday. If a prompt HVTN 127/HPTN 087 PSRT AE review cannot be completed within 72 hours of notification (excluding weekends and US federal holidays), an automatic safety pause occurs.

The HVTN and HPTN require that each CRS submit to its IRB/EC and any applicable RE protocol-related safety information (such as IND safety reports, notification of study product holds due to the pause rules, unanticipated problems involving risks to participants or others, and notification of other unplanned safety pauses). CRSs must also follow all applicable RE reporting requirements.

In addition, all other AEs are reviewed routinely by the HVTN 127/HPTN 087 PSRT (see Section 11.4.2).

11.4 Review of cumulative safety data

Routine safety review occurs at the start of enrollment and then throughout the study.

Reviews proceed from a standardized set of protocol-specific safety data reports. These reports are produced by the SDMC and include queries to the CRSs. Events are tracked by internal reports until resolution.

11.4.1 Daily review

Blinded daily safety reviews are routinely conducted by HVTN Core for events requiring expedited reporting to DAIDS, and events that meet safety pause criteria or prompt HVTN 127/HPTN 087 PSRT AE review criteria.

11.4.2 Weekly review

During the study product administration phase of the trial, the HVTN 127/HPTN 087 PSRT reviews clinical safety reports on a weekly basis and conducts calls to review the data as appropriate. Following the visit 8 weeks post-final study product administration, less frequent reporting and safety reviews may be conducted at the discretion of the HVTN 127/HPTN 087 PSRT. The HVTN CSS or HVTN Core designee reviews reports of clinical and laboratory AEs. Events identified during the review that are considered questionable, inconsistent, or unexplained are referred to the CRS clinic coordinator for verification.

11.5 Study termination

This study may be terminated early by the determination of the HVTN 127/HPTN 087 PSRT, a pertinent national regulatory authority, NIH, Office for Human Research Protections (OHRP), or study product developer(s). In addition, the conduct of this study at an individual CRS may be terminated by the determination of the IRB/EC and any applicable RE.

12 Protocol conduct

This protocol and all actions and activities connected with it will be conducted in compliance with the principles of GCP (ICHe6), the HVTN and HPTN network-specific Manuals of Operations, and DAIDS Clinical Research Policies and Standard Procedures Documents, including procedures for the following:

- Protocol registration, activation, and implementation;
- Informed consent, screening, and enrollment;
- Study participant reimbursement;
- Clinical and safety assessments;
- Safety monitoring and reporting;
- Data collection, documentation, transfer, and storage;
- Participant confidentiality;
- Study follow-up and close-out;
- Unblinding of staff and participants;
- Quality control;
- Protocol monitoring and compliance;
- Advocacy and assistance to participants regarding negative social impacts associated with participation in the trial;
- Risk reduction counseling;
- Specimen collection, processing, and analysis;
- Exploratory and ancillary studies and sub-studies, and
- Destruction of specimens.

Any policies or procedures that vary from DAIDS, HVTN, or HPTN standards or require additional instructions (eg, instructions for randomization specific to this study) will be described in the HVTN 127/HPTN 087 SSP.

12.1 Social impacts

It is possible that participants' involvement in the study could result in social impacts. For example, a participant's involvement in the study could become known to others, and a social harm may result (ie, because participants could be perceived as being HIV infected or at "high risk" for HIV infection). Participants could be treated unfairly, or could have problems being accepted by their families and/or communities. Alternatively, a social benefit may result (eg, a participant could feel good helping others).

Social harms are negative social impact events and social benefits are positive social impact events that a participant reports as affecting them as a result of being involved in a research study. It is not the researcher's opinion of how they perceive an event has affected a participant. Social impacts will be collected and reported on CRFs during scheduled visits (see Appendix G). A social harm that is reported by the participant and judged by the IoR/designee to be serious or unexpected will be reported to the responsible site's IRB at least annually, or according to their individual requirements. In the event that a participant reports a social harm, every effort will be made by study staff to provide appropriate care and counseling to the participant as necessary, and/or referral to appropriate resources for the safety and wellbeing of the participant. While maintaining participant confidentiality, study sites may engage their Community Advisory Board (CAB) in exploring the social context surrounding instances of social harms to minimize the potential occurrence of such an impact.

12.2 Emergency communication with study participants

As in all clinical research, this study may generate a need to reach participants quickly to avoid imminent harm, or to report study findings that may otherwise concern their health or welfare.

When such communication is needed, the CRS will request that its IRB/EC and any applicable RE expedite review of the message. If this review cannot be completed in a timeframe consistent with the urgency of the required communication, the site can contact the participant without IRB/EC approval if such communication is necessary to avoid imminent harm to the study participant. The CRS must notify the IRB/EC and any applicable RE of the matter as soon as possible.

13 Version history

The Protocol Team may modify the original version of the protocol. Modifications are made to Network protocols via clarification memos, letters of amendment, or full protocol amendments.

The version history of, and modifications to, Protocol HVTN 127/HPTN 087 are described below.

Protocol history and modifications

Date: March 8, 2018

Protocol version: Version 2.0
Protocol modification: Full Protocol Amendment 1

Item 1 Added on Title page: IND number

Item 2 Revised on Title page and in Section 3, Appendices A, C, and J: Study title

Item 3 Added: Intramuscular injection (IM) study arm

Item 4 Revised: Product administration and follow-up visit schedules

Item 5 Updated in Section 4.9.3: *Clinical studies of VRC07-523LS; VRC 605*

Item 6 "Pharmacokinetics" removed in Section 6.1.2, *Sample size calculations for serum levels of VRC07-523LS*

Item 7 Updated in Section 6.4.6: *Analyses and data sharing prior to end of scheduled follow-up visits*

Item 8 Updated in Sections 7, Appendix B, and Appendix D: Use of "sex assigned at birth"

Item 9 Removed in Section 9.1.2, *Protocol-specific consent forms*: Instruction to follow protocol-specific memo regarding when to start using site-specific consent forms

Item 10 Clarified in Sections 9.3 and 9.4: HIV assessment procedure includes HIV diagnostic testing

Item 11 Clarified in Section 9.8 *Assessments of Solicited AEs*: CRS clinician assessment

Item 12 Clarified in Section 9.8.1, *Assessment of systemic and local symptoms*: Thermometry

Item 13 Clarified in Section 9.8.2, *Assessment of infusion/injection site*: Infusion/injection site reaction measurements

Item 14 Updated in Section 10.1, *CRS laboratory procedures*: Special instructions and research assays

Item 15 Clarified in Section 11.2.1, *Submission of safety forms to SDMC*: Submittal deadlines

Item 16 Revised in Section 11.2.2, *AE reporting*: Unsolicited AE reporting period

Item 17 Clarified in Section 11.2.3, *Expedited reporting of AEs to DAIDS*: Unblinding procedures

Item 18 Added in Section 11.3, *Safety pause and prompt PSRT AE review*: Submission of unanticipated problems to IRB/EC

Item 19 Clarified in Section 12.2, *Emergency communication with study participants*: Circumstances under which communication is allowed prior to IRB/EC approval

Item 20 Clarified in Appendix A, Item 8: Visit intervals

Item 21 Clarified in Appendix A, Item 14: Sample testing

Item 22 Clarified in Appendix A Item 16 and Appendix C: Other research on stored samples

Item 23 Added in Appendix A and in Appendix C: Participants may change their minds regarding use of samples and data in other studies

Item 24 Clarified in footnote to Appendix A and Appendix C signature blocks: Witness requirement

Item 25 Clarified in Appendix B, *Approved birth control methods (for sample informed consent form)*: Condom use for HIV and STI prevention

Item 26 Revised in Appendix D: Table of procedures (for sample informed consent form)

Item 27 Revised in Appendix E: Product administration schedule graphic

Item 28 Revised in Appendix F: *Laboratory procedures* table

Item 29 Revised in Appendix G: *Procedures at CRS* table

Item 30 Clarified in Appendix H, *Adverse events of special interest (AESI)*: Update provisions

Item 31 Added as Appendix I: *Low risk guidelines for the US and Switzerland*

Item 32 Updated and corrected in Section 3.1: Protocol team

Item 33 Updated: Section and appendix numbers and cross-references

Item 34 Corrected: Acronyms, spelling and grammatical errors, page layout, and stylistic inconsistencies

Date: November 28, 2017

Protocol version: Version 1.0
Protocol modification: Original protocol

14 Document references (other than literature citations)

Other documents referred to in this protocol, and containing information relevant to the conduct of this study, include:

- Assessment of Understanding. Accessible through the HVTN protocol-specific website.
- Current CDC Guidelines
  - Revised Recommendations for HIV Testing of Adults, Adolescents, and Pregnant Women in Health-Care Settings. Available at http://www.cdc.gov/mmwr/PDF/rr/rr5514.pdf
  - Revised Guidelines for HIV Counseling, Testing, and Referral. Available at http://www.cdc.gov/mmwr/preview/mmwrhtml/rr5019a1.htm
- Division of AIDS (DAIDS) Clinical Research Policies and Standard Procedures Documents. Available at https://www.niaid.nih.gov/research/daids-clinical-research-policies-standard-procedures
- Division of AIDS Protocol Registration Manual. Available at https://www.niaid.nih.gov/sites/default/files/prmanual.pdf
- Division of AIDS Table for Grading the Severity of Adult and Pediatric Adverse Events. Corrected Version 2.1, July 2017. Available at http://rsc.tech-res.com/clinical-research-sites/safety-reporting/daids-grading-tables
- The Manual for Expedited Reporting of Adverse Events to DAIDS. Version 2.0, January 2010. Available at http://rsc.tech-res.com/clinical-research-sites/safety-reporting/manual
- HVTN Certificate of Confidentiality. Accessible through the HVTN website.
- HVTN 127/HPTN 087 Special Instructions. Accessible through the HVTN protocol-specific website.
- HVTN 127/HPTN 087 Study Specific Procedures. Accessible through the HVTN protocol-specific website.
- HVTN 127/HPTN 087 Site Lab Instructions. Accessible through the HVTN protocol-specific website.
- HVTN Manual of Operations. Accessible through the HVTN website.

- Dangerous Goods Regulations (updated annually), International Air Transport Association. Available for purchase at http://www.iata.org/ps/publications/dgr/Pages/index.aspx

- Lab assay algorithm (available upon request)

- International Council for Harmonisation of Technical Requirements for Pharmaceuticals for Human Use (ICH) E6, Guideline for Good Clinical Practice: Section 4.8, Informed consent of trial subjects. Available at http://www.ich.org/products/guidelines/efficacy/article/efficacy-guidelines.html

- HVTN 127/HPTN 087 Participants' Bill of Rights and Responsibilities. Accessible through the HVTN protocol-specific website.

- NIH Policy on Reporting Race and Ethnicity Data: Subjects in Clinical Research. Available at http://grants1.nih.gov/grants/guide/notice-files/NOT-OD-01-053.html

- Pharmacy Guidelines and Instructions for DAIDS Clinical Trials Networks, July 2008.

- Requirements for Source Documentation in DAIDS Funded and/or Sponsored Clinical Trials. Available at https://www.niaid.nih.gov/sites/default/files/daids-sourcedocpolicy.pdf

- Title 21, Code of Federal Regulations, Part 50. Available at http://www.accessdata.fda.gov/scripts/cdrh/cfdocs/cfcfr/CFRSearch.cfm?CFRPart=50

- Title 45, Code of Federal Regulations, Part 46. Available at http://www.hhs.gov/ohrp/humansubjects/guidance/45cfr46.html

See Section 16 for literature cited in the background and statistics sections of this protocol.

15 Acronyms and abbreviations

Ab	antibody
ACD	acid citrate dextrose
ADA	anti-drug antibody
ADCC	antibody dependent cellular cytotoxicity
ADCP	antibody dependent cellular phagocytosis
AE	adverse event
AESI	adverse events of special interest
AIA	anti-idiotypic antibody
ALT	alanine aminotransferase
AMP	antibody mediated prevention
ANOVA	analysis of variance
aPTT	activated partial thromboplastin time
ART	antiretroviral therapy
AST	aspartate aminotransferase
ATI	analytic treatment interruption
AUC	area under the curve
BAMA	binding antibody multiplex assay
β-HCG	beta human chorionic gonadotropin
bNAb	broadly neutralizing antibody
CAB	Community Advisory Board
CBC	complete blood count
CDC	US Centers for Disease Control and Prevention
CFR	Code of Federal Regulations
CIOMS	Council for International Organizations of Medical Sciences
CL	clearance
Cmax	maximum concentration
CMIA	chemiluminescent microparticle immunoassay
CRF	case report form
CRPMC	NIAID Clinical Research Products Management Center
CRS	clinical research site
CSR	central specimen repository
CSS	Clinical Safety Specialist
DAERS	DAIDS Adverse Experience Reporting System
DAIDS	(NIAID, NIH) Division of AIDS
DEHP	di(2-ethylhexyl)phthalate
DHHS	US Department of Health and Human Services
EAE	expedited AE
EC	ethics committee

EDTA	ethylenediaminetetraacetic acid
EIA	enzyme immunoassay
ELISA	enzyme-linked immunosorbent assay
Fc	fragment crystallizable
FcRn	neonatal Fc receptor
FDA	US Food and Drug Administration
Fred Hutch	Fred Hutchinson Cancer Research Center
GCP	Good Clinical Practice
GEE	generalized estimating equation
GINA	Genetic Information Nondiscrimination Act
GLP	Good Laboratory Practice
HBsAg	hepatitis B surface antigen
HCV	hepatitis C virus
Hg	mercury
HIPAA	Health Insurance Portability and Accountability Act
HIV	human immunodeficiency virus
HPTN	HIV Prevention Trials Network
HVTN	HIV Vaccine Trials Network
ICF	informed consent form
ICH	International Council for Harmonisation of Technical Requirements for Pharmaceuticals for Human Use
IgG	immunoglobulin G
IND	Investigational New Drug
IoR	Investigator of Record
IRB	institutional review board
IUD	intrauterine device
IVIG	intravenous immunoglobulin
IV	intravenous
mAb	monoclonal antibody
MAR	missing at random
MCAR	missing completely at random
MedDRA	Medical Dictionary for Regulatory Activities
MITT	modified intent-to-treat
MSD	Meso Scale Discovery
nAb	neutralizing antibody
NAEPP	National Asthma Education and Prevention Program
NHP	nonhuman primate
NIAID	National Institute of Allergy and Infectious Diseases (US NIH)
NIH	US National Institutes of Health
NSAID	nonsteroidal anti-inflammatory drug

NOAEL	no observed adverse effects level
OHRP	US Office for Human Research Protections
PAB	DAIDS Pharmaceutical Affairs Branch
PBMC	peripheral blood mononuclear cell
PCR	polymerase chain reaction
PEP	post-exposure prophylaxis
PES	polyethersulfone
PI	Principal Investigator
PK	pharmacokinetic
PP	per protocol
PrEP	pre-exposure prophylaxis
PSRT	Protocol Safety Review Team
RAB	DAIDS Regulatory Affairs Branch
RE	regulatory entity
RSC	(DAIDS) Regulatory Support Center
RSV	respiratory syncytial virus
SAE	serious adverse event
SC	subcutaneous
SCHARP	Statistical Center for HIV/AIDS Research and Prevention
SCIG	subcutaneous immunoglobulin
SD	standard deviation
SDMC	statistical and data management center
SHIV	simian human immunodeficiency virus
SMB	Safety Monitoring Board
SPT	DAIDS Safety and Pharmacovigilance Team
SSP	Study Specific Procedures
SST	serum-separating tube
SUSAR	suspected unexpected serious adverse reaction
TasP	treatment as prevention
TB	tuberculosis
TCR	tissue cross reactivity
Tmax	time to Cmax
USP	United States Pharmacopeia
UW-VSL	University of Washington Virology Specialty Laboratory
V	volume of distribution
VITL	Vaccine Research Center- Immunology Testing Laboratory
VRC	Vaccine Research Center (NIAID)

16 Literature cited

1. UNAIDS. Ethical considerations in biomedical HIV prevention trials. 2007 7/2007.

2. The National Commission for the Protection of Human Subjects of Biomedical and Behavioral Research. The Belmont Report: Ethical Principles and Guidelines for the Protection of Human Subjects of Research. 1979 4/18/1979.

3. Council for International Organizations of Medical Sciences (CIOMS). International ethical guidelines for biomedical research involving human subjects. Bull Med Ethics. 2002(182):17-23.

4. UNAIDS. Report on the Global AIDS Epidemic, 2012 country progress reports 2013 [updated 2013. Available from: http://www.unaids.org/en/dataanalysis/knowyourresponse/countryprogressreports/2012countries/.

5. UNAIDS. Global Report: Report on the global AIDS epidemic 2013. 2013 2013. Report No.: UNAIDS / JC2502/1/E.

6. WHO/UNICEF/UNAIDS. Global Report on HIV Treatment 2013: Results, Impact and Opportunities. 2014.

7. UNAIDS. Local Epidemics Issues Brief. 2014 2014.

8. UNAIDS. Global AIDS Update. 2016.

9. Cohen MS, Chen YQ, McCauley M, Gamble T, Hosseinipour MC, Kumarasamy N, et al. Prevention of HIV-1 infection with early antiretroviral therapy. N Engl J Med. 2011;365(6):493-505.

10. Grant RM, Lama JR, Anderson PL, McMahan V, Liu AY, Vargas L, et al. Preexposure chemoprophylaxis for HIV prevention in men who have sex with men. N Engl J Med. 2010;363(27):2587-99.

11. Grant RM, Smith DK. Integrating Antiretroviral Strategies for Human Immunodeficiency Virus Prevention: Post- and Pre-Exposure Prophylaxis and Early Treatment. Open Forum Infect Dis. 2015;2(4):ofv126.

12. Fauci AS, Marston HD. Ending AIDS--is an HIV vaccine necessary? N Engl J Med. 2014;370(6):495-8.

13. Gray GE, Laher F, Lazarus E, Ensoli B, Corey L. Approaches to preventative and therapeutic HIV vaccines. Curr Opin Virol. 2016;17:104-9.

14. Hey A. History and Practice: Antibodies in Infectious Diseases. Microbiol Spectr. 2015;3(2):AID-0026-2014.

15. Graham BS, Ambrosino DM. History of passive antibody administration for prevention and treatment of infectious diseases. Curr Opin HIV AIDS. 2015;10(3):129-34.

16. Winokur PL, Stapleton JT. Immunoglobulin prophylaxis for hepatitis A. Clin Infect Dis. 1992;14(2):580-6.

17. Fiore AE, Wasley A, Bell BP. Prevention of hepatitis A through active or passive immunization: recommendations of the Advisory Committee on Immunization Practices (ACIP). MMWR Recomm Rep. 2006;55(RR-7):1-23.

18. Manning SE, Rupprecht CE, Fishbein D, Hanlon CA, Lumlertdacha B, Guerra M, et al. Human rabies prevention--United States, 2008: recommendations of the Advisory Committee on Immunization Practices. MMWR Recomm Rep. 2008;57(RR-3):1-28.

19. McLean HQ, Fiebelkorn AP, Temte JL, Wallace GS, Centers for Disease C, Prevention. Prevention of measles, rubella, congenital rubella syndrome, and mumps, 2013: summary recommendations of the Advisory Committee on Immunization Practices (ACIP). MMWR Recomm Rep. 2013;62(RR-04):1-34.

20. Centers for Disease C, Prevention. Updated recommendations for use of VariZIG--United States, 2013. MMWR Morb Mortal Wkly Rep. 2013;62(28):574-6.

21. Simoes EA. Immunoprophylaxis of respiratory syncytial virus: global experience. Respir Res. 2002;3 Suppl 1:S26-33.

22. Li Y, Migueles SA, Welcher B, Svehla K, Phogat A, Louder MK, et al. Broad HIV-1 neutralization mediated by CD4-binding site antibodies. Nat Med. 2007;13(9):1032-4.

23. Simek MD, Rida W, Priddy FH, Pung P, Carrow E, Laufer DS, et al. Human immunodeficiency virus type 1 elite neutralizers: individuals with broad and potent neutralizing activity identified by using a high-throughput neutralization assay together with an analytical selection algorithm. J Virol. 2009;83(14):7337-48.

24. Wu X, Yang ZY, Li Y, Hogerkorp CM, Schief WR, Seaman MS, et al. Rational design of envelope identifies broadly neutralizing human monoclonal antibodies to HIV-1. Science. 2010;329(5993):856-61.

25. Zhou T, Georgiev I, Wu X, Yang ZY, Dai K, Finzi A, et al. Structural basis for broad and potent neutralization of HIV-1 by antibody VRC01. Science. 2010;329(5993):811-7.

26. Gray ES, Taylor N, Wycuff D, Moore PL, Tomaras GD, Wibmer CK, et al. Antibody specificities associated with neutralization breadth in plasma from human immunodeficiency virus type 1 subtype C-infected blood donors. J Virol. 2009;83(17):8925-37.

27. Gray ES, Madiga MC, Hermanus T, Moore PL, Wibmer CK, Tumba NL, et al. The neutralization breadth of HIV-1 develops incrementally over four years and is associated with CD4+ T cell decline and high viral load during acute infection. J Virol. 2011;85(10):4828-40.

28. Corti D, Langedijk JP, Hinz A, Seaman MS, Vanzetta F, Fernandez-Rodriguez BM, et al. Analysis of memory B cell responses and isolation of novel monoclonal antibodies with neutralizing breadth from HIV-1-infected individuals. PLoS ONE. 2010;5(1):e8805.

29. Walker LM, Huber M, Doores KJ, Falkowska E, Pejchal R, Julien JP, et al. Broad neutralization coverage of HIV by multiple highly potent antibodies. Nature. 2011;477(7365):466-70.

30. Scheid JF, Mouquet H, Ueberheide B, Diskin R, Klein F, Oliveira TY, et al. Sequence and structural convergence of broad and potent HIV antibodies that mimic CD4 binding. Science. 2011;333(6049):1633-7.

31. Binley JM, Lybarger EA, Crooks ET, Seaman MS, Gray E, Davis KL, et al. Profiling the specificity of neutralizing antibodies in a large panel of plasmas from patients chronically infected with human immunodeficiency virus type 1 subtypes B and C. J Virol. 2008;82(23):11651-68.

32. Sather DN, Armann J, Ching LK, Mavrantoni A, Sellhorn G, Caldwell Z, et al. Factors associated with the development of cross-reactive neutralizing antibodies during human immunodeficiency virus type 1 infection. J Virol. 2009;83(2):757-69.

33. Falkowska E, Ramos A, Feng Y, Zhou T, Moquin S, Walker LM, et al. PGV04, an HIV-1 gp120 CD4 binding site antibody, is broad and potent in neutralization but does not induce conformational changes characteristic of CD4. J Virol. 2012;86(8):4394-403.

34. Walker LM, Phogat SK, Chan-Hui PY, Wagner D, Phung P, Goss JL, et al. Broad and potent neutralizing antibodies from an African donor reveal a new HIV-1 vaccine target. Science. 2009;326(5950):285-9.

35. Walker LM, Simek MD, Priddy F, Gach JS, Wagner D, Zwick MB, et al. A limited number of antibody specificities mediate broad and potent serum

neutralization in selected HIV-1 infected individuals. PLoS Pathog. 2010;6(8):e1001028.

36. McLellan JS, Pancera M, Carrico C, Gorman J, Julien JP, Khayat R, et al. Structure of HIV-1 gp120 V1/V2 domain with broadly neutralizing antibody PG9. Nature. 2011;480(7377):336-43.

37. Kong L, Lee JH, Doores KJ, Murin CD, Julien JP, McBride R, et al. Supersite of immune vulnerability on the glycosylated face of HIV-1 envelope glycoprotein gp120. Nat Struct Mol Biol. 2013;20(7):796-803.

38. Huang J, Ofek G, Laub L, Louder MK, Doria-Rose NA, Longo NS, et al. Broad and potent neutralization of HIV-1 by a gp41-specific human antibody. Nature. 2012;491(7424):406-12.

39. Stamatatos L, Morris L, Burton DR, Mascola JR. Neutralizing antibodies generated during natural HIV-1 infection: good news for an HIV-1 vaccine? Nat Med. 2009;15(8):866-70.

40. Walker LM, Burton DR. Rational antibody-based HIV-1 vaccine design: current approaches and future directions. Curr Opin Immunol. 2010;22(3):358-66.

41. Burton DR, Ahmed R, Barouch DH, Butera ST, Crotty S, Godzik A, et al. A Blueprint for HIV Vaccine Discovery. Cell Host Microbe. 2012;12(4):396-407.

42. Burton DR, Stanfield RL, Wilson IA. Antibody vs. HIV in a clash of evolutionary titans. Proc Natl Acad Sci U S A. 2005;102(42):14943-8.

43. Kwong PD, Mascola JR. Human antibodies that neutralize HIV-1: identification, structures, and B cell ontogenies. Immunity. 2012;37(3):412-25.

44. Georgiev IS, Gordon JM, Zhou T, Kwong PD. Elicitation of HIV-1-neutralizing antibodies against the CD4-binding site. Curr Opin HIV AIDS. 2013;8(5):382-92.

45. Pegu A, Hessell AJ, Mascola JR, Haigwood NL. Use of broadly neutralizing antibodies for HIV-1 prevention. Immunol Rev. 2017;275(1):296-312.

46. Wu X, Wang C, O'Dell S, Li Y, Keele BF, Yang Z, et al. Selection pressure on HIV-1 envelope by broadly neutralizing antibodies to the conserved CD4-binding site. J Virol. 2012;86(10):5844-56.

47. Lynch RM, Boritz E, Coates EE, DeZure A, Madden P, Costner P, et al. Virologic effects of broadly neutralizing antibody VRC01 administration during chronic HIV-1 infection. Sci Transl Med. 2015;7(319):319ra206.

48. Ledgerwood JE, Coates EE, Yamshchikov G, Saunders JG, Holman L, Enama ME, et al. Safety, pharmacokinetics and neutralization of the broadly neutralizing HIV-1 human monoclonal antibody VRC01 in healthy adults. Clin Exp Immunol. 2015;182(3):289-301.

49. Huang Y, Zhang L, Ledgerwood J, Grunenberg N, Bailer R, Isaacs A, et al. Population pharmacokinetics analysis of VRC01, an HIV-1 broadly neutralizing monoclonal antibody, in healthy adults. MAbs. 2017:1-9.

50. Bar KJ, Sneller MC, Harrison LJ, Justement JS, Overton ET, Petrone ME, et al. Effect of HIV Antibody VRC01 on Viral Rebound after Treatment Interruption. N Engl J Med. 2016;375(21):2037-50.

51. Pyzik M, Rath T, Lencer WI, Baker K, Blumberg RS. FcRn: The Architect Behind the Immune and Nonimmune Functions of IgG and Albumin. J Immunol. 2015;194(10):4595-603.

52. Roopenian DC, Akilesh S. FcRn: the neonatal Fc receptor comes of age. Nat Rev Immunol. 2007;7(9):715-25.

53. Roopenian DC, Christianson GJ, Sproule TJ, Brown AC, Akilesh S, Jung N, et al. The MHC class I-like IgG receptor controls perinatal IgG transport, IgG homeostasis, and fate of IgG-Fc-coupled drugs. J Immunol. 2003;170(7):3528-33.

54. Akilesh S, Christianson GJ, Roopenian DC, Shaw AS. Neonatal FcR expression in bone marrow-derived cells functions to protect serum IgG from catabolism. J Immunol. 2007;179(7):4580-8.

55. Gupta S, Gach JS, Becerra JC, Phan TB, Pudney J, Moldoveanu Z, et al. The Neonatal Fc receptor (FcRn) enhances human immunodeficiency virus type 1 (HIV-1) transcytosis across epithelial cells. PLoS Pathog. 2013;9(11):e1003776.

56. Gupta S, Pegu P, Venzon DJ, Gach JS, Ma ZM, Landucci G, et al. Enhanced in vitro transcytosis of simian immunodeficiency virus mediated by vaccine-induced antibody predicts transmitted/founder strain number after rectal challenge. J Infect Dis. 2015;211(1):45-52.

57. Yoshida M, Kobayashi K, Kuo TT, Bry L, Glickman JN, Claypool SM, et al. Neonatal Fc receptor for IgG regulates mucosal immune responses to luminal bacteria. J Clin Invest. 2006;116(8):2142-51.

58. Tzaban S, Massol RH, Yen E, Hamman W, Frank SR, Lapierre LA, et al. The recycling and transcytotic pathways for IgG transport by FcRn are distinct and display an inherent polarity. J Cell Biol. 2009;185(4):673-84.

59. Li Z, Palaniyandi S, Zeng R, Tuo W, Roopenian DC, Zhu X. Transfer of IgG in the female genital tract by MHC class I-related neonatal Fc receptor

(FcRn) confers protective immunity to vaginal infection. Proc Natl Acad Sci U S A. 2011;108(11):4388-93.

60. Armitage CW, O'Meara CP, Harvie MC, Timms P, Blumberg RS, Beagley KW. Divergent outcomes following transcytosis of IgG targeting intracellular and extracellular chlamydial antigens. Immunol Cell Biol. 2014;92(5):417-26.

61. Ko SY, Pegu A, Rudicell RS, Yang ZY, Joyce MG, Chen X, et al. Enhanced neonatal Fc receptor function improves protection against primate SHIV infection. Nature. 2014;514(7524):642-5.

62. Rudicell RS, Kwon YD, Ko SY, Pegu A, Louder MK, Georgiev IS, et al. Enhanced potency of a broadly neutralizing HIV-1 antibody in vitro improves protection against lentiviral infection in vivo. J Virol. 2014;88(21):12669-82.

63. Zalevsky J, Chamberlain AK, Horton HM, Karki S, Leung IW, Sproule TJ, et al. Enhanced antibody half-life improves in vivo activity. Nat Biotechnol. 2010;28(2):157-9.

64. Lowy I, Molrine DC, Leav BA, Blair BM, Baxter R, Gerding DN, et al. Treatment with monoclonal antibodies against Clostridium difficile toxins. N Engl J Med. 2010;362(3):197-205.

65. Chung RT, Gordon FD, Curry MP, Schiano TD, Emre S, Corey K, et al. Human monoclonal antibody MBL-HCV1 delays HCV viral rebound following liver transplantation: a randomized controlled study. Am J Transplant. 2013;13(4):1047-54.

66. Huang Y, Karuna ST, Janes H, Frahm N, Nason M, Edlefsen PT, et al. Use of placebos in Phase 1 preventive HIV vaccine clinical trials. Vaccine. 2015;33(6):749-52.

67. LaCasce AC, MC; Burstein,H; Meyerhardt,JA. Infusion-related reactions to therapeutic monoclonal antibodies used for cancer therapy 2017 [Available from: https://www.uptodate.com/contents/infusion-related-reactions-to-therapeutic-monoclonal-antibodies-used-for-cancer-therapy?source=search_result&search=infusion%20reaction&selectedTitle=2~150#H10.

68. Hansel TT, Kropshofer H, Singer T, Mitchell JA, George AJ. The safety and side effects of monoclonal antibodies. Nat Rev Drug Discov. 2010;9(4):325-38.

69. Bugelski PJ, Achuthanandam R, Capocasale RJ, Treacy G, Bouman-Thio E. Monoclonal antibody-induced cytokine-release syndrome. Expert Rev Clin Immunol. 2009;5(5):499-521.

70. Vogel WH. Infusion reactions: diagnosis, assessment, and management. Clin J Oncol Nurs. 2010;14(2):E10-E21.

71. Haddad E, Barnes D, Kafal A. Home therapy with subcutaneous immunoglobulins for patients with primary immunodeficiency diseases. Transfus Apher Sci. 2012;46(3):315-21.

72. Resch B. Product review on the monoclonal antibody palivizumab for prevention of respiratory syncytial virus infection. Hum Vaccin Immunother. 2017:0.

73. Frogel MP, Stewart DL, Hoopes M, Fernandes AW, Mahadevia PJ. A systematic review of compliance with palivizumab administration for RSV immunoprophylaxis. J Manag Care Pharm. 2010;16(1):46-58.

74. Agresti A, Coull BA. Approximate is better than "exact" for interval estimation of binomial proportions. Am Stat. 1998;52(2):119-26.

75. Hughes JP. Mixed effects models with censored data with application to HIV RNA levels. Biometrics. 1999;55(2):625-9.

76. Rotnitzky A, Robins J. Analysis of semi-parametric regression models with non-ignorable non-response. Stat Med. 1997;16(1-3):81-102.

77. Montefiori DC. Measuring HIV neutralization in a luciferase reporter gene assay. Methods Mol Biol. 2009;485:395-405.

78. Ozaki DA, Gao H, Todd CA, Greene KM, Montefiori DC, Sarzotti-Kelsoe M. International technology transfer of a GCLP-compliant HIV-1 neutralizing antibody assay for human clinical trials. PLoS ONE. 2012;7(1):e30963.

79. Seaman M, Janes H, Hawkins N, randpre L, Devoy C, Giri A, et al. Tiered Categorization of a Diverse Panel of HIV-1 Env Pseudoviruses for Assessment of Neutralizing Antibodies. J Virol. 2010;84(3):1439-52.

80. DeCamp A, Hraber P, Bailer RT, Seaman MS, Ochsenbauer C, Kappes J, et al. Global panel of HIV-1 Env reference strains for standardized assessments of vaccine-elicited neutralizing antibodies. J Virol. 2014;88(5):2489-507.

Appendix A Sample informed consent form

Title: A multicenter, randomized, partially blinded phase 1 clinical trial to evaluate the safety and serum concentrations of a human monoclonal antibody, VRC-HIVMAB075-00-AB (VRC07-523LS), administered in multiple doses and routes to healthy, HIV-uninfected adults<br>Protocol number: HVTN 127/HPTN 087<br>Site: [Insert site name]

Thank you for your interest in our research study. Please read this consent form or ask someone to read it to you. If you decide to join the study, we will ask you to sign or make your mark on this form. We will offer you a copy to keep. We will ask you questions to see if we have explained everything clearly. You can also ask us questions about the study.

Research is not the same as treatment or medical care. The purpose of a research study is to answer scientific questions.

About the study

The HIV Vaccine Trials Network (HVTN), the HIV Prevention Trials Network (HPTN) and [Insert site name] are doing a study to test the antibody against HIV called VRC07-523LS. HIV is the virus that causes AIDS. Antibodies are one of the ways the human body fights infection. Antibodies are natural proteins that the body can make to prevent infectious agents such as bacteria and viruses from making you sick. Researchers can also make antibodies in laboratories and give them to people intravenously (with an IV) or by injection. We will tell you more about these procedures below. Antibodies have been used successfully to prevent or treat some other health problems, such as a virus that causes respiratory infections in infants.

About 124 people will take part in this study at multiple sites. The researcher in charge of this study at this clinic is [Insert name of site PI]. The US National Institutes of Health (NIH) is paying for the study.

1. We are doing this study to answer several questions.

- Is the VRC07-523LS antibody safe to give to people?
- Are people able to take the antibody without becoming too uncomfortable?
- How much of the antibody remains in the body as time passes?
- How does the body's response to the antibody change depending on the dose given?
- Does the method of giving the antibody change the body's response?

2. The antibody cannot give you HIV.

The study antibody is not made from actual HIV. It is impossible for the antibody to give you HIV. Also, it cannot cause you to give HIV to someone else. We do not know if the antibody will decrease, increase, or not change your chance of becoming infected with HIV if you are exposed to the virus.

3. The study antibody is experimental.

The formal name of the study antibody is VRC-HIVMAB075-00-AB. From here on, we will call it VRC07-523LS or the study antibody.

VRC07-523LS is an experimental product. That means we do not know if it will be safe to use in people, or if it will work to prevent HIV infection. VRC07-523LS is only used in research studies.

VRC07-523LS was developed by Vaccine Research Center at the US National Institutes of Health (NIH).

In laboratory and animal studies, the study antibody attached to and prevented infection by many kinds of HIV viruses from around the world. We do not know if it will act the same way when given to people. It will take many studies to learn if it will be useful for prevention of HIV or treatment of HIV. This study will not answer these questions.

Risks of the VRC07-523LS antibody:

This section lists the side effects we know about. There may be others that we don’t yet know about, even serious ones. We will tell you if we learn about any new side effects.

The VRC07-523LS antibody has been tested for safety in animals. In animal studies, no safety problems were seen at doses 20 times higher than those used in this study.

As of early August 2017, VRC07-523LS has been given by subcutaneous injection (given under the skin) or by intravenous infusion (IV) to 25 people in an ongoing clinical trial at the NIH Clinical Center. So far, the study antibody has not made people too uncomfortable or caused serious health problems. This is the first clinical trial that will test giving the antibody to people by intramuscular injection (given in the muscle). We will describe these procedures in Section 10 of this form.

As of July 2017, similar antibodies called VRC01 and VRC01LS have been given to people in 14 clinical studies in the United States, Peru, Brazil, Switzerland, Thailand, and in Sub-Saharan Africa. In these studies, more than 2400 adults and 33 infants have gotten these study products.

Many of these studies are still going on and we don't know which people got the study antibody and which people got a placebo (a liquid with no antibody in it). After receiving the antibody or a placebo, many people said that they had mild pain, itching, or redness where the antibody or placebo was given to them. Of these people, some said they felt like they had the flu after getting the antibody, but that this feeling lasted a few hours at most.

In a previous study, one person who got a study antibody by injection had a rash. One person had a brief fainting spell several hours after getting a study antibody by IV infusion. To be safe, no more injections or infusions were given to these people. Some participants had mild body discomfort, muscle pain, or joint pain after getting the study antibody.

The study antibody has not been given to people by intramuscular injection before. It has been tested in rats at the same dose as in this study. No safety problems were seen in rats. Even if something looks like it is safe in animals, this may not be true for people.

General risks of antibodies:

Antibodies that are different from VRC07-523LS have been given to people for other illnesses. With those antibodies most side effects happen within the first 24 hours. Those antibodies have caused fever, stuffy nose, redness in the face, feeling weak or having low energy, chills, shaking, nausea, vomiting, pain, headache, dizziness, trouble breathing, high or low blood pressure, itchiness, rash, hives, lip, tongue, throat or face swelling, diarrhea, racing heartbeat, or chest pain.

Rarely, some antibodies have caused serious reactions that may be life-threatening. Two such serious reactions are:

- Anaphylaxis – a physical reaction that includes difficulty breathing, possibly leading to low blood oxygen, low blood pressure, hives or rash, and swelling in the mouth and face. This may occur soon after getting an antibody

- Serum Sickness – a physical reaction that includes developing hives or a rash, fever, big lymph nodes, muscle and joint pains, chest discomfort and shortness of breath. This may occur several days to 3 weeks after getting an antibody

Please tell us if you have ever experienced reactions similar to anaphylaxis or serum sickness, and the cause of the reactions if you remember.

Rarely, antibodies licensed for treatment of other diseases have been linked to a blood disorder that interferes with blood clotting, to cancer, to damage to the heart muscle, and to the body's immune system attacking healthy cells.

These rare side effects and reactions have not been seen in other studies with VRC07-523LS or similar experimental antibodies.

Antibodies given to a person usually do not last in the body more than a few months. One of the goals of this study is to see how long VRC07-523LS will stay in the body. We don't know yet how long it will last, but it may be several months.

Joining the study

4. It is completely up to you whether or not to join the study.

Take your time in deciding. If it helps, talk to people you trust, such as your doctor, friends or family. If you decide not to join this study, or if you leave it after you have joined, your other care at this clinic and the benefits or rights you would normally have will not be affected.

If you join this study, you may not be allowed to join some other kinds of HIV prevention studies now or in the future. You cannot be in this study while you are in another study where you get a study product. Also during the study, you should not donate blood or tissue.

If you choose not to join this study, you may be able to join another study.

Site: Remove item 5 if you use a separate screening consent that covers these procedures.

5. If you want to join the study, we will screen you to see if you are eligible.

Screening involves a physical exam, HIV test and health history. A physical exam may include, but is not limited to:

- Checking your weight, temperature and blood pressure
- Looking in your mouth and throat
- Listening to your heart and lungs
- Feeling your abdomen (stomach and liver)
- Checking your veins to see how easy it might be to start an IV

We will also do blood and urine tests. These tests tell us about some aspects of your health, such as how healthy your kidneys, liver, and immune system are. We will also test you for syphilis, hepatitis B, and hepatitis C. We will ask you about medications you are taking. We will ask you about behaviors that might put you at risk for getting HIV.

If you are able to become pregnant, we will test you for pregnancy. If you have had your uterus or ovaries removed (a hysterectomy or oophorectomy), verified by medical records, you are not required to have a pregnancy test.

We will review the screening results with you. The screening results may show you are not eligible to join the study, even if you want to.

Site: adapt the following section so it is applicable to the care available at your site

6. **If we find that you have a health problem during screening or during the study, we will tell you about the care that we can give here for free.**

   For the care that we cannot give, we will explain how we will help you get care elsewhere. For health problems that are unrelated to the study, we will not pay for care.

7. **If you are able to become pregnant, you must agree to use birth control to join this study.**

Site: If you want to include Appendix B, Approved birth control methods (for sample informed consent form), in this consent form, paste it below and delete paragraph below.

You should not become pregnant during the study because we do not know how the study antibody could affect the developing baby. You must agree to use effective birth control from 21 days before your first injection or IV infusion through your last scheduled clinic visit. We will talk to you about effective birth control methods. They are listed on a handout that we will give to you.

Being in the study

If you meet the study requirements and want to join, here is what will happen:

Site: Modify the number of visits and range of visit lengths to be site-specific. This is to account for variation in screening protocols and in the number of possible follow-up visits between protocol-mandated visits.

8. **You will come to the clinic for 19 scheduled visits for a little over 2 years.**

   Most of the visits will be about 8 weeks apart. We will also ask you to come to the clinic about 3 days after the first injection or infusion and then again about 6 days after to draw your blood. We will do this so that we can look at how your body responds to the study product.

   Visits can last from [#] to [#] hours.

   You may have to come for more visits if you have a lab or health issue.

   We may contact you after the main study ends (for example, to tell you about the study results).

9. We will give you [Site: Insert compensation] for each study visit you complete.

This amount is to cover the costs of [Site: Insert text]

Site: Insert any costs to participants (eg, birth control costs for female participants who could become pregnant).

US sites: Include the following paragraph:

Payments you receive for being in the study may be taxable. We may need to ask you for your Social Security number for tax reasons.

You do not have to pay anything to be in this study.

10. We will give you the study products by IV or injection.

There are 6 groups in this study. People in the different groups will get the study products either by IV or by injection. The amount of antibody is different in the different groups. In one group, some people will get a placebo.

When getting an IV, a sterile needle is used to place a small plastic tube into a vein in your arm. The tube is connected to a small bag of fluid that contains the antibody. An IV pump controls how fast the fluid drips from the bag, through the tube, and into your vein. The first IV will take about one hour. Other IVs will probably take about 15 to 30 minutes each, but could take up to an hour.

When getting a subcutaneous (SC) injection, a sterile needle is put under the skin on your arm, abdomen, or thigh. For an intramuscular (IM) injection, a sterile needle is put directly into either your gluteus (buttock) or deltoid (upper arm) muscle. You will get up to 4 injections at each injection visit. You can ask the study staff more about this.

Which group you are in and whether you get the study antibody by IV or SC or IM injection is completely random, like flipping a coin. Whether you get the study antibody or the placebo is also random. We have no say in which group you are assigned to.

If you get IM injections, you will have to wait until everyone completes their final study visits to find out whether you got the antibody or the placebo. This could be several years. If you have a serious medical problem and need to know what you got before the end of the study, we can tell you.

11. We will give the IVs and injections to all participants on the same schedule.

You will get an IV or injections five times, about once every 4 months.

IV and Injection Schedule					
Groups	First IV or injection	4 months later	8 months later	12 months later	16 months later
1 - 6	X	X	X	X	X

You will have to wait in the clinic for about an hour after the first IV or injection and for about half an hour after the other IVs or injections to see if there are any problems. Then for that night and for 3 more days, you will need to keep track of how you are feeling and if you have any symptoms. We will ask you the ways we can contact you. We will contact you about 3 days after each IV or injection to ask how you have been feeling. Contact the clinic staff if you have any issues or concerns after getting an IV or an injection. If you have a problem, we will continue to check on you until it goes away.

12. In addition to giving you the study antibody or a placebo, we will:

- Do regular HIV testing, as well as counseling on your results and on how to avoid getting HIV
- Do physical exams
- Do pregnancy tests if you are able to become pregnant
- Ask questions about your health, including medications you may be taking
- Ask questions about any personal problems or benefits you may have from being in the study
- Take urine and blood samples.

When we take blood, the amount will depend on the lab tests we need to do. It will be some amount between 34 mL and 160 mL (a little more than 2 tablespoon to 2/3 cup). Your body will make new blood to replace the blood we take out.

Site: You may want to add a sentence to the end of the previous paragraph contextualizing the blood volumes described (eg, "To compare, people who donate blood in the US can give a total of about 500 mL in an 8-week period."). Modify the example for cultural relevance and alter blood volumes as necessary.

Site: Insert Appendix D, Table of procedures (for informed consent form) in this section or distribute it as a separate sheet if it is helpful to your study participants. You are not required to do either.

We will be looking for side effects. We will review the results of these procedures and tests with you at your next visit, or sooner if necessary. If any of the results are important to your health, we will tell you.

13. We will counsel you on avoiding HIV infection.

We will ask you personal questions about your HIV risk factors such as sexual behavior, alcohol, and drug use. We will talk with you about ways to keep your risk of getting HIV low.

14. We will test your samples in this study.

We will send your samples (without your name) to labs approved by the HVTN and HPTN for this study, which are located in the United States and South Africa. In rare cases, some of your samples may be sent to labs approved by the HVTN in other countries for research related to this study.

The samples will be tested to:

- Measure how much antibody is in your blood, and

- See how your immune system responds to the study antibody.

Researchers may also do genetic testing related to this study on your samples. Your genes are passed to you from your birth parents. They affect how you look and how your body works. The differences in people’s genes can help explain why some people get a disease while others do not. The genetic testing will only involve some of your genes, not all of your genes (your genome). The researchers will study only the genes related to the immune system and HIV and those that affect how people get HIV.

If you become HIV infected, the researchers may look at all of the genes of the virus found in your samples. The researchers will use this information to learn more about HIV and the study product(s).

In some cases, researchers may take cells from your samples and grow more of them over time, so that they can continue to contribute to this study.

These tests done on your samples are for research purposes, not to check your health. The labs will not give the results to you or this clinic because their tests are not approved for use in making health care decisions. These labs are only approved to do research tests.

When your samples are no longer needed for this study, the HVTN will continue to store them.

Site: Delete next section if using separate consent for use of samples and information in other studies

15. When samples are no longer needed for this study, the study sponsors want to keep them for use in other studies by HVTN, HPTN, or other researchers.

This section gives you information so you can decide if you want your extra samples and information used in other studies. You will mark your decision at the end of the form. If you have any questions, please ask.

Do I have to agree? No. You are free to say yes or no, or to change your mind after you sign this form. At your request, we will destroy all extra samples that we have. Your decision will not affect your being in this study or have any negative consequences here.

Where are the samples stored? Extra samples are stored in a secure central place called a repository. Your samples will be stored in the HVTN repository in the United States.

How long will the samples be stored? There is no limit on how long your extra samples will be stored. *[Site: Revise the previous sentence to insert limits if your regulatory authority imposes them.]*

Will I be paid for the use of my samples? No. Also, a researcher may make a new scientific discovery or product based on the use of your samples. If this happens, there is no plan to share any money with you. The researcher is not likely to ever know who you are.

Will I benefit from allowing my samples to be used in other studies? Probably not. Results from these other studies are not given to you, this clinic, or your doctor. They are not part of your medical record. The studies are only being done for research purposes.

Will the HVTN or HPTN sell my samples and information? No, but the HVTN and HPTN may share your samples with other researchers. Once we share your samples and information, we may not be able to get them back.

How do other researchers get my samples and information? When a researcher wants to use your samples and information, their research plan must be approved by the HVTN and HPTN. Also, the researcher's institutional review board (IRB) or ethics committee (EC) will review their plan. *[Site: If review by your institution's IRB/EC/RE is also required, insert a sentence stating this.]* IRBs/ECs protect the rights and well-being of people in research. If the research plan is approved, the HVTN will send your samples to the researcher's location.

What information is shared with other researchers? The samples and information will be labeled with a code number. Your name will not be part of the information. However, some information that we share may be personal, such as your race, ethnicity, sex, health information from the study, and HIV status. We may share information about the study product you received and how your body responded to the study product.

What kind of studies might be done with my extra samples and information? The studies will be related to HIV prevention or infection, the immune system and other diseases.

Researchers may also do genetic testing on your samples.

In some cases, researchers may take cells from your samples and grow more of them over time, so that they can continue to do research with them.

If you agree, your samples could also be used for genome wide studies. In these studies, researchers will look at all of your genes (your genome). The researchers compare the genomes of many people, looking for common patterns of genes that could help them understand diseases. The researchers may put the information from the genome-wide studies into a protected database so that other researchers can access it, but your name and other personal information will not be included. Usually, no one would be able to look at your genome and link it to you as a person. However, if another database exists that also has information on your genome and your name, someone might be able to compare the databases and identify you. If others found out, it could lead to discrimination or other problems. The risk of this is very small. There may be other unknown risks.

Who will have access to my information in studies using my extra samples?

People who may see your information are:

- Researchers who use your extra samples and information for other research
- Government agencies that fund or monitor the research using your extra samples and information
- Any regulatory agency that reviews clinical trials,
- The researcher’s Institutional Review Board or Ethics Committee
- The people who work with the researcher

All of these people will do their best to protect your information. The results of any new studies that use your extra samples and information may be published. No publication will use your name or identify you personally.

16. We will do our best to protect your private information.

US sites: Check HIPAA authorization for conflicts with this section.

Your study records and samples will be kept in a secure location. We will label all of your samples and most of your records with a code number, not your name or other personal information. However, it is possible to identify you, if necessary. We will not share your name with the lab that does the tests on your samples, or with anyone else who does not need to know your name.

Clinic staff will have access to your study records. Your records may also be reviewed by groups who watch over this study to see that we are protecting your rights, keeping you safe, and following the study plan. These groups include:

- The US National Institutes of Health and its study monitors,
- The US Food and Drug Administration,
- Any regulatory agency that reviews clinical trials,
- [Insert name of local IRB/EC] ,
- [Insert name of local and/or national regulatory authority as appropriate],
- The NIH Vaccine Research Center and people who work for them,
- The HVTN, HPTN, and people who work for them,
- The HVTN Safety Monitoring Board, and
- The US Office for Human Research Protections.

All reviewers will take steps to keep your records private.

We cannot guarantee absolute privacy. At this clinic, we have to report the following information:

Site: Include any public health or legal reporting requirements. Bulleted examples should include all appropriate cases (reportable communicable disease, risk of harm to self or others, etc.).

- [Item 1]
- [Item 2]
- [Item 3]

US sites: Include the following boxed text. You can remove the box.

We have a Certificate of Confidentiality from the US government, to help protect your privacy. With the certificate, we do not have to release information about you to someone who is not connected to the study, such as the courts or police. Sometimes we can't use the certificate. Since the US government funds this research, we cannot withhold information from it. Also, you can still release information about yourself and your study participation to others.

The results of this study may be published. No publication will use your name or identify you personally.

We may share information from the study with other researchers. We will not share your name or information that can identify you.

A description of this clinical trial will be available on http://www.ClinicalTrials.gov. This website will not include information that can identify you. At most, the website will include a summary of the results. You can search this website at any time.

17. We may stop your IVs or injections or take you out of the study at any time. We may do this even if you want to stay in the study and even if you were scheduled for more injections.

This may happen if:

- you do not follow instructions,
- we think that staying in the study might harm you,
- you get HIV,
- you enroll in a different research study where you get another study product, or
- the study is stopped for any reason.

If we stop your injections, we may ask you to stay in the study to complete other study procedures.

18. We will stop your IV infusions or injections if you become pregnant.

We will encourage you to stay in the study if you choose. We will discuss your study options with you.

If you leave the study while you are still pregnant, we will contact you after your due date to ask some questions about your pregnancy and delivery.

19. If you get infected with HIV during the study, we will help you get care and support.

You will not be able to stay in this study. We will counsel you about your HIV infection and about telling your partner(s). We will tell you where you can get support and medical care. *Site: Modify the following sentence as appropriate.* We will not provide or pay for any of your HIV care directly.

Other Risks

20. There are other risks to being in this study.

This section describes the other risks and restrictions we know about. There may also be unknown risks, even serious ones. We will tell you if we learn anything new that may affect your willingness to stay in the study.

Risks of giving blood and receiving injections:

In this study, we will do some routine medical procedures. These include taking blood from you and giving you injections. These procedures can cause bruising, pain, fainting, soreness, redness, swelling, itching, a sore, bleeding, and (rarely) muscle damage or infection where you got the injection. Giving blood can cause a low blood cell count (anemia), making you feel tired.

Risks of IV infusion procedures:

Getting an IV may cause stinging, discomfort, pain, soreness, redness, bruising, itching, rash and swelling where the needle goes into the skin. Rarely, needle sticks can result in a blood clot or infection.

Personal problems/discrimination/testing HIV antibody positive:

Some people who join HVTN and HPTN studies report personal problems or discrimination because of joining an HIV prevention study. Family or friends may worry, get upset or angry, or assume that you are infected with HIV or at high risk and treat you unfairly as a result. Rarely, a person has lost a job because the study took too much time away from work, or because their employer thought they had HIV.

HIV testing

HIV antibody tests are the usual way to test for HIV infections. We do not expect you to test positive on HIV antibody tests. We have used several common HIV antibody tests to test samples of blood containing very similar antibodies and none of them detected the antibody.

To be absolutely safe we ask you to get HIV tests only at this clinic during the study. Our tests can always detect true HIV infection. They can also tell if someone is really not HIV infected. Since the antibodies do not last long in the body, we do not expect you to have any problems with HIV testing after the study ends.

Although it has not been seen so far, getting VRC07-523LS may cause common HIV antibody tests to show that someone is HIV-negative, even if they are actually infected.

Embarrassment/anxiety:

You may feel embarrassed when we ask about your HIV risks, such as having sex and using drugs. Also, waiting for your HIV test results or other health test results could make you feel anxious. You could feel worried if your test results show that you are infected with HIV. If you feel embarrassed or anxious, please tell us and we will try to help you.

Risks of disclosure of your personal information:

We will take several steps to protect your personal information. Although the risk is very low, it is possible that your personal information could be given to someone who should not have it. If that happened, you could face discrimination, stress, and embarrassment. We can tell you more about how we will protect your personal information if you would like it.

Risks of genetic testing:

It is unlikely, but the genetic tests done on your samples could show you may be at risk for certain diseases. If others found out, it could lead to discrimination or other problems. However, it is almost impossible for you or others to know your test results from the genetic testing. The results are not part of your study records and are not given to you.

U.S. Sites, include the following paragraph In the very unlikely event that your genetic information becomes linked to your name, a federal law called the Genetic Information Nondiscrimination Act (GINA) helps protect you. GINA keeps health insurance companies and employers from seeing results of genetic testing when deciding about giving you health insurance or offering you work. GINA does not help or protect you against discrimination by companies that sell life, disability or long-term care insurance.

Unknown risks:

We do not know if the study antibody will increase, decrease, or not change your risk of becoming infected with HIV if exposed. If you get infected with HIV, we do not know how the study antibody might affect your HIV infection or how long it takes to develop AIDS.

We do not know how the study antibody will affect a pregnant participant or a developing baby.

Benefits

21. The study may not benefit you.

We do not expect getting the study antibody to benefit you in any way. However, being in the study might still help you in some ways. The counseling that you get as part of the study may help you avoid getting HIV. The lab tests and physical exams that you get while in this study might detect health problems you don't yet know about.

This study may help in the search for a drug or a vaccine to prevent HIV. However, if the study antibody or a vaccine later gets approved and sold, there are no plans to share any money with you.

Your rights and responsibilities

22. If you join the study, you have rights and responsibilities.

You have many rights that we will respect. You also have responsibilities. We list these in the Participant's Bill of Rights and Responsibilities. We will give you a copy of it.

Leaving the study

23. Tell us if you decide to leave the study.

You are free to leave the study at any time and for any reason. Your care at this clinic and your legal rights will not be affected, but it is important for you to let us know.

We will ask you to come back to the clinic one last time for a physical exam, and we may ask to take some blood and urine samples. We will also ask about any personal problems or benefits you have experienced from being in the study. We believe these steps are important to protecting your health, but it is up to you whether to complete them.

Injuries

Sites: Approval from HVTN Regulatory Affairs (at vtn.core.reg@hvtn.org) is needed for any change (other than those that the instructions specifically request or those previously approved by HVTN Regulatory Affairs) to the boxed text

24. If you get sick or injured during the study, contact us immediately.

Your health is important to us. *(Sites: adjust the following 2 sentences if applicable to the care available at your site)* We will tell you about the care that we can give here. For the care that we cannot provide, we will explain how we will help you get care elsewhere.

If you become sick or injured in this study, there is a process to decide if it is related to the study antibody and/or procedures. If it is, we call it a study-related injury. There are funds to pay for treatment of study-related injuries if certain conditions are met.

The HVTN and HPTN have limited funds to pay medical costs for study-related injuries that it determines are reasonable. *(Sites: insert locale- appropriate medical insurance language in the following sentence)* If the injury is not study related, then you and your health insurance will be responsible for treatment costs.

Some injuries are not physical. For example, you might be harmed emotionally by being in an HIV prevention study. Or you might lose wages because you cannot go to work. However, there are no funds to pay for these kinds of injuries, even if they are study related.

You may disagree with the decision about whether your injury is study related. If you wish, independent experts will be asked to review the decision. You always have the right to use the court system if you are not satisfied.

Questions

25. If you have questions or problems at any time during your participation in this study, use the following important contacts.

If you have questions about this study, contact
[name or title and telephone number of the investigator or other study staff].

If you have any symptoms that you think may be related to this study, contact
[name or title and telephone number of the investigator or other study staff].

This study has been reviewed and approved by a committee called the
[name of local IRB/EC]. If you have questions about your rights as a research participant, or problems or concerns about how you are being treated in this study, contact [name or title and telephone number of person on IRB/EC] , at the committee.

Your permissions and signature

Site: Delete the following section if using a separate consent for use of samples and information in other studies

26. In Section 15 of this form, we told you about possible other uses of your extra samples and information, outside this study. Please choose only one of the options below and write your initials or make your mark in the box next to it. Whatever you choose, the HVTN and HPTN keep track of your decision about how your samples and information can be used. You can change your mind after signing this form.

☐ I allow my extra samples and information to be used for other studies related to HIV prevention, the immune system, and other diseases. This may include genetic testing and keeping my cells growing over time.

OR

☐ I agree to the option above *and* also to allow my extra samples and information to be used in genome wide studies.

OR

☐ I do not allow my extra samples to be used in any other studies. This includes not allowing genetic testing, growing more of my cells, or genome wide studies.

27. If you agree to join this study, you will need to sign or make your mark below. Before you sign or make your mark on this consent form, make sure of the following:

- You have read this consent form, or someone has read it to you.
- You feel that you understand what the study is about and what will happen to you if you join. You understand what the possible risks and benefits are.
- You have had your questions answered and know that you can ask more.
- You agree to join this study.

You will not be giving up any of your rights by signing this consent form.

Participant's name (print)	Participant's signature or mark	Date	Time
Clinic staff conducting consent discussion (print)	Clinic staff signature	Date	Time

For participants who are unable to read or write, a witness should complete the signature block below:

Witness's name (print)	Witness's signature	Date	Time

*Witness is impartial and was present for the entire discussion of this consent form.

Appendix B Approved birth control methods (for sample informed consent form)

Site: Any change to the following boxed text requires approval from HVTN Regulatory Affairs at vtn.core.reg@hvtn.org.

You should not become pregnant during the study because we do not know how the study antibody could affect the developing baby.

You must agree to use effective birth control from 21 days before your first IV infusion or injection until after your last scheduled clinic visit.

Effective birth control means using any of the following methods every time you have sex:

- Birth control drugs that prevent pregnancy—given by pills, shots, patches, vaginal rings, or inserts under the skin;
- Male or female condoms, with or without a cream or gel that kills sperm;
- Diaphragm or cervical cap with a cream or gel that kills sperm;
- Intrauterine device (IUD); or
- Any other contraceptive method approved by the researchers.

You do not have to use birth control if:

- You are only having sex with a partner or partners who have had a vasectomy. (We will ask you some questions to confirm that the vasectomy was successful.);
- You have reached menopause, with no menstrual periods for one year;
- You have had a hysterectomy (your uterus removed);
- You have had your ovaries removed;
- You have a tubal ligation (your “tubes tied”) or confirmed successful placement of a product that blocks the fallopian tubes;
- You are having sex only with a partner or partners assigned female sex at birth;
- You only have oral sex; or,
- You are sexually abstinent (no sex at all).

Remember: If you are having sex, male and female condoms are the only birth control methods that also provide protection against HIV and other sexually transmitted infections.

If you join the study, we will test you for pregnancy at some visits, including before each study injection.

Appendix C Sample consent form for use of samples and information in other studies

Title: A multicenter, randomized, partially blinded phase 1 clinical trial to evaluate the safety and serum concentrations of a human monoclonal antibody, VRC-HIVMAB075-00-AB (VRC07-523LS), administered in multiple doses and routes to healthy, HIV-uninfected adults<br>Protocol number: HVTN 127/HPTN 087<br>Site: [Insert site name]

When samples are no longer needed for this study, the study sponsors want to keep them for use in other studies by HVTN, HPTN, or other researchers. This section gives you information so you can decide if you want your extra samples and information used in other studies. You will mark your decision at the end of the form. If you have any questions, please ask.

1. Do I have to agree?

No. You are free to say yes or no, or to change your mind after you sign this form. At your request, we will destroy all extra samples that we have. Your decision will not affect your being in this study or have any negative consequences here.

2. Where are the samples stored?

Extra samples are stored in a secure central place called a repository. Your samples will be stored in the HVTN repository in the United States.

3. How long will the samples be stored?

There is no limit on how long your extra samples will be stored. *[Site: Revise the previous sentence to insert limits if your regulatory authority imposes them.]*

4. Will I be paid for the use of my samples?

No. Also, a researcher may make a new scientific discovery or product based on the use of your samples. If this happens, there is no plan to share any money with you. The researcher is not likely to ever know who you are.

5. Will I benefit from allowing my samples to be used in other studies?

Probably not. Results from these other studies are not given to you, this clinic, or your doctor. They are not part of your medical record. The studies are only being done for research purposes.

6. Will the HVTN or HPTN sell my samples and information?

No, but the HVTN and HPTN may share your samples with other researchers. Once we share your samples and information, we may not be able to get them back.

7. How do other researchers get my samples and information?

When a researcher wants to use your samples and information, their research plan must be approved by the HVTN and HPTN. Also, the researcher’s institutional review board (IRB) or ethics committee (EC) will review their plan. *[Site: If review by your institution’s IRB/EC/RE is also required, insert a sentence stating this.]* IRBs/ECs protect the rights and well-being of people in research. If the research plan is approved, the HVTN will send your samples to the researcher’s location.

8. What information is shared with other researchers?

The samples and information will be labeled with a code number. Your name will not be part of the information. However, some information that we share may be personal, such as your race, ethnicity, gender, health information from the study, and HIV status. We may share information about the study product you received and how your body responded to the study product.

9. What kind of studies might be done with my extra samples and information?

The studies will be related to HIV prevention or infection, the immune system, and other diseases.

Researchers may also do genetic testing on your samples.

In some cases, researchers may take cells from your samples and grow more of them over time, so that they can continue to do research with them.

If you agree, your samples could also be used for genome wide studies. In these studies, researchers will look at all of your genes (your genome). The researchers compare the genomes of many people, looking for common patterns of genes that could help them understand diseases. The researchers may put the information from the genome-wide studies into a protected database so that other researchers can access it, but your name and other personal information will not be included. Usually, no one would be able to look at your genome and link it to you as a person. However, if another database exists that also has information on your genome and your name, someone might be able to compare the databases and identify you. If others found out, it could lead to discrimination or other problems. The risk of this is very small. There may be other unknown risks.

10. What are the risks of genetic testing?

It is unlikely, but the genetic tests done on your samples could show you may be at risk for certain diseases. If others found out, it could lead to discrimination or other problems. However, it is almost impossible for you or others to know your test results from the genetic testing. The results are not part of your study records and are not given to you.

U.S. Sites, include the following paragraph

In the very unlikely event that your genetic information becomes linked to your name, a federal law called the Genetic Information Nondiscrimination Act (GINA) helps protect you. GINA keeps health insurance companies and employers from seeing results of genetic testing when deciding about giving you health insurance or offering you work. GINA does not help or protect you against discrimination by companies that sell life, disability or long-term care insurance.

11. Who will have access to my information in studies using my extra samples?

People who may see your information are:

- Researchers who use your extra samples and information for other research
- Government agencies that fund or monitor the research using your extra samples and information
- Any regulatory agency that reviews clinical trials,
- The researcher’s Institutional Review Board or Ethics Committee
- The people who work with the researcher

All of these people will do their best to protect your information. The results of any new studies that use your extra samples and information may be published. No publication will use your name or identify you personally.

Questions

12. If you have questions or problems about allowing your samples and information to be used in other studies, use the following important contacts.

If you have questions about the use of your samples or information or if you want to change your mind about their use, contact
[name or title and telephone number of the investigator or other study staff].

If you think you may have been harmed because of studies using your samples or information, contact
[name or title and telephone number of the investigator or other study staff].

If you have questions about your rights as a research participant, contact [name or title and telephone number of person on IRB/EC .

13. Please choose only one of the options below and write your initials or make your mark in the box next to it. Whatever you choose, the HVTN and HPTN keep track of your choice about how your samples and information can be used. You can change your mind after signing this form.

☐ I allow my extra samples and information to be used for other studies related to HIV prevention, the immune system, and other diseases. This may include genetic testing and keeping my cells growing over time.

OR

☐ I agree to the option above *and* also to allow my extra samples and information to be used in genome wide studies.

OR

☐ I do not allow my extra samples to be used in any other studies. This includes not allowing genetic testing, growing more of my cells, or genome wide studies.

Participant's name (print)	Participant's signature or mark	Date	Time

Clinic staff conducting consent discussion (print)	Clinic staff signature	Date	Time

For participants who are unable to read or write, a witness should complete the signature block below:

Witness's name (print)	Witness's signature	Date	Time

*Witness is impartial and was present for the entire discussion of this consent form

Appendix D Table of procedures (for sample informed consent form)

6 mos 1 yr 1½ yrs 2 yrs

					Weeks after first IV or injection															
Procedure	Screening visit(s)	First IV or injection visit	3 days	6 days	4	8	12	16	24	32	40	48	56	64	72	80	88	96	104	112
IV infusion or injection		√						√		√		√		√						
Medical history	√																			
Complete physical	√																			√
Brief physical		√				√		√	√	√	√	√	√	√						
Urine test	√					√									√					
Blood drawn	√	√	√	√	√	√	√	√	√	√	√	√	√	√	√	√	√	√	√	√
Pregnancy test*	√	√						√		√		√		√					√	
HIV testing and pretest counseling	√						√		√		√		√		√		√		√	√
Risk reduction counseling	√	√				√		√	√	√	√	√	√	√						√
Interview/questionnaire	√	√				√		√	√	√	√	√	√	√	√			√		√

Not shown in this table is a time after all study participants have completed their last scheduled visit when you can find out what products you received.

* For persons assigned female sex at birth and who are capable of becoming pregnant. Persons who have had a total hysterectomy (removal of the uterus) or removal of both ovaries (verified by medical records), are not required to have a pregnancy test.

Appendix E Schema graphic (for sample informed consent form)

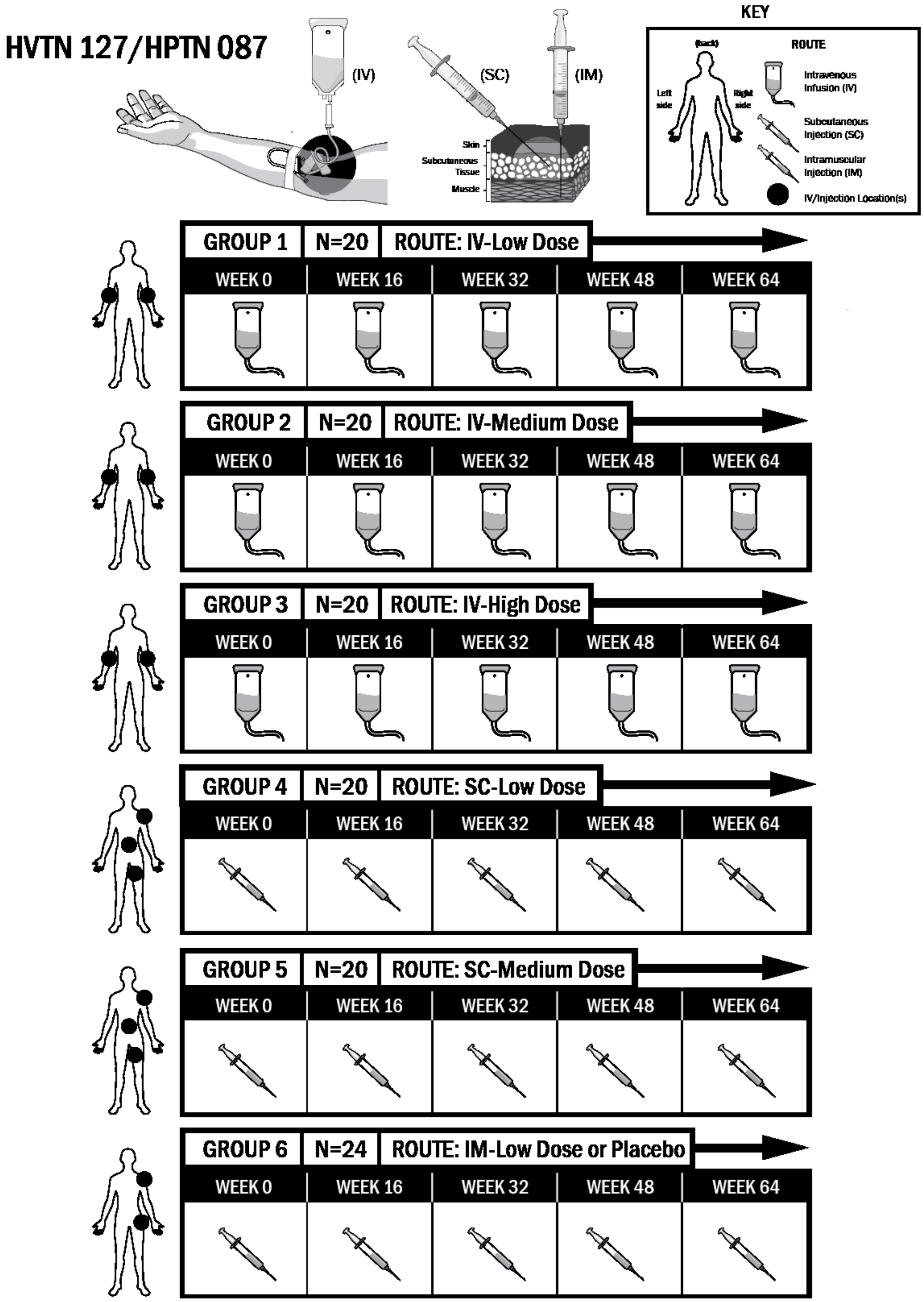

Appendix F Laboratory procedures

Procedure	Ship to[1]	Assay location[2]	Tube Type[4]	Tube size (vol. capacity)[4]	1	2	3	4	5	6	7	8	9	10	11	12	13	14	15	16	17	18	19	20	Total
				Visit:	1	2	3	4	5	6	7	8	9	10	11	12	13	14	15	16	17	18	19	20	
				Day:	Screening visit[3]	D0	D3	D6	D28	D56	D84	D112	D168	D224	D280	D336	D392	D448	D504	D560	D616	D672	D728	D784	
				Week:		W0			W4	W8	W12	W16	W24	W32	W40	W48	W56	W64	W72	W80	W88	W96	W104	W112	
						**Inf or Inj #1**						**Inf or Inj #2**		**Inf or Inj #3**		**Inf or Inj #4**		**Inf or Inj #5**							
BLOOD COLLECTION																									
Screening/Diagnostic																									
Screening HIV test	Local lab	Local lab	EDTA	5mL	5	—	—	—	—	—	—	—	—	—	—	—	—	—	—	—	—	—	—	—	**5**
HBsAg/anti-HCV	Local lab	Local lab	SST	5mL	5	—	—	—	—	—	—	—	—	—	—	—	—	—	—	—	—	—	—	—	**5**
Syphilis	Local lab	Local lab	SST	5mL	5	—	—	—	—	—	—	—	—	—	—	—	—	—	—	—	—	—	—	—	**5**
HIV diagnostics[8]	UW-VSL	UW-VSL	EDTA	10mL	—	—	—	—	—	—	10	—	10	—	10	—	10	—	10	—	10	—	10	20	**90**
Safety labs																									
CBC/ Differential	Local lab	Local lab	EDTA	5mL	5	5	—	—	—	5	—	5	5	5	5	5	5	5	5	—	—	—	5	—	**60**
Chemistry Panel[5]	Local lab	Local lab	SST	5mL	5	5	—	—	—	5	—	5	5	5	5	5	5	5	5	—	—	—	5	—	**60**
Drug levels																									
VRC07-523LS concentration	CSR	HVTN Labs / VITL	SST	8.5mL	—	8.5	8.5	8.5	8.5	8.5	8.5	8.5	8.5	8.5	8.5	8.5	8.5	8.5	8.5	8.5	8.5	8.5	8.5	8.5	**161.5**
Host genetics[6]	CSR	HVTN Labs	ACD	8.5mL	—	z	—	—	—	—	—	—	—	—	—	—	—	—	—	—	—	—	—	—	**0**
Humoral assays																									
ADA detection assay (MSD)	CSR	VITL	SST	8.5mL	—	y	—	—	—	—	—	y	—	y	—	y	—	y	—	y	y	y	y	y	**0**
ADA functional assay (NAb)	CSR	HVTN Labs	SST	8.5mL	—	y	—	—	—	—	—	y	—	y	—	y	—	y	—	y	y	y	y	y	**0**
Binding Ab assay	CSR	HVTN Labs	SST	8.5mL	—	y	—	—	—	y	—	—	y	—	y	—	y	—	y	—	y	—	—	—	**0**
Neutralizing Ab assay	CSR	HVTN Labs	SST	8.5mL	—	8.5	—	—	—	8.5	—	—	8.5	—	8.5	—	8.5	—	8.5	—	8.5	—	—	—	**59.5**
STORAGE																									
Serum	CSR	—	SST	8.5mL	—	34	25.5	25.5	25.5	17	25.5	25.5	17	25.5	17	25.5	17	25.5	17	25.5	17	25.5	25.5	25.5	**442**
PBMC	CSR	—	ACD	8.5mL	—	85	—	—	—	—	—	—	—	68	—	—	—	68	—	—	—	—	—	85	**306**
**Visit total**					**25**	**146**	**34**	**34**	**34**	**44**	**44**	**44**	**54**	**112**	**54**	**44**	**54**	**112**	**54**	**34**	**44**	**34**	**54**	**139**	**1194**
**56-Day total**					**25**	**171**	**205**	**239**	**273**	**317**	**122**	**132**	**98**	**166**	**166**	**98**	**98**	**166**	**166**	**88**	**78**	**78**	**88**	**193**	
**URINE COLLECTION**																									
Urine dipstick[9]	Local lab	Local lab			X	—	—	—	—	X	—	—	—	—	—	—	—	—	X	—	—	—	—	—	
Pregnancy Test[7]	Local lab	Local lab			X	X	—	—	—	—	—	X	—	X	—	X	—	X	—	—	—	—	X	—	

Inf = Infusion

Inj = Injection

[1] CSR = central specimen repository; UW-VSL = University of Washington Virology Specialty Laboratory (Seattle, Washington, USA).

[2] HVTN Laboratories include: Fred Hutchinson Cancer Research Center (Seattle, Washington, USA); Duke University Medical Center (Durham, North Carolina, USA). Non-HVTN laboratories: VITL = Vaccine Research Center – Immunology Testing Laboratory (Gaithersburg, Maryland, USA).

[3] Screening may occur over the course of several contacts/visits up to and including day 0 prior to study product administration.

[4] Local labs may assign appropriate alternative tube types for locally performed tests.

[5] Chemistry panel is defined in Section 9.2 (pre-enrollment).

[6] Genotyping may be performed on enrolled participants using cryopreserved PBMC collected at baseline.

[7] For a participant who was assigned female sex at birth, pregnancy test must be performed on urine or blood specimens on the day of study product infusion/injection with negative results received prior to infusion/injection. Persons who have undergone total hysterectomy or bilateral oophorectomy (verified by medical records), are not required to undergo pregnancy testing.

[8] At an early termination visit for a withdrawn or terminated participant (see Section 9.10), blood should be drawn for HIV diagnostic testing, as shown for visit 23 above.

[9] And microscopy if needed.

y = SST blood collected for neutralizing Ab assays and serum storage will also cover specimen needs for the anti-VRC07-523LS concentrations, ADA detection assay (MSD), ADA functional assay (NAb), and binding Ab assays; no separate blood draw is needed.

z = ACD blood collected for PBMC storage will also cover specimen needs for host genetics assay; no separate blood draw is needed.

Appendix G Procedures at CRS

Visit	01[1]	02[2]	03	04	05	06	07	08	09	10	11	12	13	14	15	16	17	18	19	20	Post
**Day:**		D0	D3	D6	D28	D56	D84	D112	D168	D224	D280	D336	D392	D448	D504	D560	D616	D672	D728	D924	
**Week:**		W0			W4	W8	W12	W16	W24	W32	W40	W48	W56	W64	W72	W80	W88	W96	W104	W112	
**Procedure**	Scr.	Inf/ Inj1						Inf/ Inj2		Inf/ Inj3		Inf/ Inj4		Inf/ Inj5							
**Study procedures[3]**																					
Signed screening consent (if used)	X	—	—	—	—	—	—	—	—	—	—	—	—	—	—	—	—	—	—	—	—
Assessment of understanding	X	—	—	—	—	—	—	—	—	—	—	—	—	—	—	—	—	—	—	—	—
Signed protocol consent	X	—	—	—	—	—	—	—	—	—	—	—	—	—	—	—	—	—	—	—	—
Medical history	X	—	—	—	—	—	—	—	—	—	—	—	—	—	—	—	—	—	—	—	—
Complete physical exam	X	—	—	—	—	—	—	—	—	—	—	—	—	—	—	—	—	—	—	X	—
Confirm eligibility, obtain demographics, randomize	X	—	—	—	—	—	—	—	—	—	—	—	—	—	—	—	—	—	—	—	—
Abbreviated physical exam	—	X	—	—	—	X	—	X	X	X	X	X	X	X	—	—	—	—	—	—	—
Risk reduction counseling	X	X	—	—	—	X	—	X	X	X	X	X	X	X	—	—	—	—	—	X	—
Contraception assessment[4]	X	X	—	—	—	X	—	X	X	X	X	X	X	X	—	—	—	—	—	X	—
Behavioral risk assessment questionnaire	X	—	—	—	—	—	—	—	X	—	—	X	—	—	X	—	—	—	—	X	—
Social impact assessment	—	X	—	—	—	X	—	X	X	X	X	X	X	X	X	—	—	X	—	X	—
Social impact assessment questionnaire	—	—	—	—	—	—	—	—	X	—	—	X	—	—	X	—	—	—	—	X	—
Acceptability questionnaire	—	X	—	—	—	—	—	X	—	X	—	X	—	X	—	—	—	—	—	—	—
Belief questionnaire[5]	—	—	—	—	—	—	—	—	—	—	—	—	—	—	—	—	—	—	—	X	—
Concomitant medications	X	X	—	—	—	X	—	X	X	X	X	X	X	X	—	—	—	—	—	X	—
Intercurrent illness/Unsolicited adverse experience	—	X	—	—	—	X	—	X	X	X	X	X	X	X	X	X	—	—	—	—	—
AESI	—	—	—	—	—	—	—	—	—	—	—	—	—	—	—	—	X	X	X	X	—
HIV infection assessment[6]	X	—	—	—	—	—	X	—	X	—	X	—	X	—	X	—	X	—	X	X	—
Confirm HIV test results provided to participant	—	X	—	—	—	—	—	X	—	X	—	X	—	X	—	X	—	X	—	X	X
**Study product administration**																					
Infusion/Injection[7]	—	X	—	—	—	—	—	X	—	X	—	X	—	X	—	—	—	—	—	—	—
Solicited AE assessment[8]	—	X	—	—	—	—	—	X	—	X	—	X	—	X	—	—	—	—	—	—	—
**Local lab assessments**																					
Screening HIV test	X	—	—	—	—	—	—	—	—	—	—	—	—	—	—	—	—	—	—	—	—
Hepatitis B, Hepatitis C	X	—	—	—	—	—	—	—	—	—	—	—	—	—	—	—	—	—	—	—	—
Syphilis	X	—	—	—	—	—	—	—	—	—	—	—	—	—	—	—	—	—	—	—	—
CBC, differential	X	X	—	—	—	X	—	X	X	X	X	X	X	X	X	—	—	—	—	—	—
Chemistry panel[9]	X	X	—	—	—	X	—	X	X	X	X	X	X	X	X	—	—	—	—	—	—
Urine dipstick[10]	X	—	—	—	—	X	—	—	—	—	—	—	—	—	X	—	—	—	—	—	—
Pregnancy (urine or serum HCG)[11]	X	X	—	—	—	—	—	X	—	X	—	X	—	X	—	—	—	—	—	—	—
**Poststudy**																					
Unblind participant	—	—	—	—	—	—	—	—	—	—	—	—	—	—	—	—	—	—	—	—	X

[1] Screening may occur over the course of several contacts/visits up to and including day 0 prior to study product administration.
[2] Specimens collected at Day 0 may be obtained within the 14 days prior to study product administration, except for a pregnancy test which must be performed on urine or blood specimens within 24 hours prior to study product administration with negative results received prior to study product administration.
[3] For specimen collection requirements, see Appendix F.
[4] Pregnancy prevention (contraception) assessment is required only for participants who were assigned female at birth and who are capable of becoming pregnant. For such participants, use of effective contraception is required from 21 days prior to the first study product administration until the last scheduled clinic visit.
[5] Group 6 participants only.
[6] Includes pre-test counseling and HIV testing. A subsequent follow-up contact is conducted to provide post-test counseling and to report results to participant.
[7] Blood draws required at study product administration visit must be performed prior to administration of study product; however, it is not necessary to have results prior to administration, except for results of a serum pregnancy test, if indicated. Lab tests may be drawn with the 3 days prior to study product administration.
[8] Solicited AE assessments performed daily for at least 3 days following study product administration (see Section 9.8).
[9] Chemistry panels are defined in Section 9.2.
[10] And microscopy if needed.
[11] For a participant who was assigned female sex at birth, pregnancy test must be performed on urine or blood specimens within 24 hours prior to study product infusion/injection with negative results received prior to infusion/injection. Persons who have undergone total hysterectomy or bilateral oophorectomy (verified by medical records), are not required to undergo pregnancy testing.

Appendix H Adverse events of special interest (AESI)

AEs of special interest (AESI) for this protocol include those listed below. Updates to AESI will be provided in the *HVTN 127/HPTN 087 SSP*.

- Systemic lupus erythematosus (SLE)
- Systemic scleroderma (SSD) (Systemic sclerosis [SSc]), including diffuse systemic form and CREST syndrome
- Sjogren’s syndrome (SS)
- Polymyositis/Dermatomyositis syndrome (PM/DM)
- Raynaud’s syndrome
- Antiphospholipid antibody syndrome (APS)
- Idiopathic thrombocytopenic purpura (ITP)
- Crohn’s disease
- Ulcerative colitis

Appendix I Low risk guidelines for the US and Switzerland

The following are intended as guidelines for the investigator to help identify potential vaccine trial participants at "low risk" for HIV infection. These guidelines are based on behaviors within the last 6-12 months prior to enrollment; however, it may be appropriate to consider a person's behavior over a longer period of time than specified to assess the person's likelihood of maintaining low risk behavior. Some volunteers may not be appropriate for enrollment even if they meet these guidelines. These guidelines should be supplemented and interpreted with local epidemiologic information about HIV prevalence in your area and community networks. The investigator may review the risk level of any volunteer with the site PI and/or the Protocol Safety Review Team.

A volunteer may be appropriate for inclusion if he/she meets these guidelines:

1. Sexual behaviors

In the **<u>last 12 months</u>** <u>did not</u>:

- Have oral, vaginal or anal intercourse with an HIV-infected partner, or a partner who uses injection drugs
- Give or receive money, drugs, gifts or services in exchange for oral, vaginal or anal sex

AND

In the **last 6 months** has abstained from penile/anal or penile/vaginal intercourse, OR

In the **last 6 months**:

- Had 4 or fewer partners of the opposite birth sex for vaginal and/or anal intercourse, OR

Is an MSM (person born male with partner(s) born male) who, in the **<u>last 12 months</u>**:

- Had 2 or fewer MSM partners for anal intercourse and had no unprotected anal sex with MSM, OR
- Had unprotected anal intercourse with only 1 MSM partner, within a monogamous relationship lasting at least 12 months (during which neither partner had any other partners). If the monogamous relationship ended, the volunteer may then have had protected anal intercourse with 1 other MSM partner (total 2 or fewer partners in the last 12 months).

Is a transgender person, regardless of the point on the transition spectrum, having sex with men (born male) and/or other transgender persons, who in the **<u>last 12 months</u>**:

- Had 2 or fewer partners for anal or vaginal intercourse, and had no unprotected anal or vaginal sex, OR
- Had unprotected anal or vaginal intercourse sex with 1 partner only within a monogamous relationship lasting at least 12 months (during which neither partner had any other partners). If the monogamous relationship ended, may then have had protected anal or vaginal sex with 1 other partner (total 2 or fewer partners in the last 12 months).

AND

Uses or intends to use condoms in situations which may include penile/anal or penile/vaginal intercourse with new partners of unknown HIV status, occasional partners, partners outside a primary relationship, and/or partners known to have other partners.

2. Non-sexual behaviors

In the **last 12 months** did not:

- Inject drugs or other substances without a prescription
- Use cocaine, methamphetamine, or excessive alcohol, which in the investigator's judgment, rendered the participant at greater than low risk for acquiring HIV infection. The investigator's judgment should consider local epidemiologic information about HIV prevalence in the area and community networks.

A volunteer is NOT appropriate for inclusion if he/she:

Acquired an STI (i.e. new infection) in the last 12 months:

- Syphilis
- Gonorrhea
- Non-gonococcal urethritis
- Herpes Simplex Virus type 2 (HSV2)
- Chlamydia
- Pelvic inflammatory disease (PID)
- Trichomonas
- Mucopurulent cervicitis
- Epididymitis
- Proctitis
- Lymphogranuloma venereum
- Chancroid
- Hepatitis B

Appendix J Protocol Signature Page

A multicenter, randomized, partially blinded phase 1 clinical trial to evaluate the safety and serum concentrations of a human monoclonal antibody, VRC-HIVMAB075-00-AB (VRC07-523LS), administered in multiple doses and routes to healthy, HIV-uninfected adults

I will conduct the study in accordance with the provisions of this protocol and all applicable protocol-related documents. I agree to conduct this study in compliance with United States (U.S.) Health and Human Service regulations (45 CFR 46); applicable U.S. Food and Drug Administration regulations; standards of the International Conference on Harmonization Guideline for Good Clinical Practice (E6); Institutional Review Board/Ethics Committee determinations; all applicable in-country, state, and local laws and regulations; and other applicable requirements (eg, US National Institutes of Health, Division of AIDS) and institutional policies

Investigator of Record Name (print)	Investigator of Record Signature	Date

DAIDS Protocol Number: HVTN 127/HPTN 087

DAIDS Protocol Version: Version 2.0

Protocol Date: March 8, 2018
